# Plasma GDF15 affects long-term dementia risk and alters neuroimmune signaling

**DOI:** 10.1126/sciadv.aec7614

**Published:** 2026-06-26

**Authors:** Cassandra O. Blew, Michael R. Duggan, Dimitrios Tsitsipatis, Gabriela T. Gomez, Zulema Rodriguez-Hernandez, Luke C. Pilling, Jingsha Chen, Eva Jacobsen, Heather E. Dark, Yifei Lu, Shannon M. Drouin, Cassandra M. Joynes, Minhao Yao, Murat Bilgel, Abhay Moghekar, Qu Tian, Julián Candia, Mary Kaileh, Aditi Gupta, Krystyna Mazan-Mamczarz, Myriam Gorospe, Alexey Lyashkov, Yevgeniya Lukyanenko, Mika Kivimaki, Philipp Frank, Lori L. Jennings, Valborg Gudmundsdottir, Vilmundur Gudnason, Lenore J. Launer, Naoto Kaneko, Shintaro Kato, Makio Furuichi, Masaki Shibayama, Masahisa Katsuno, Keita Hiraga, Yukiko Nishita, Rei Otsuka, James R. Pike, Mary R. Rooney, Pascal Schlosser, Yuhan Cui, Guray Erus, Christos Davatzikos, Rebecca F. Gottesman, Iwao Waga, Priya Palta, Christie Ballantyne, Michael Griswold, Zhonghua Liu, Luigi Ferrucci, Allison B. Herman, Keenan A. Walker

**Affiliations:** ^1^ Laboratory of Behavioral Neuroscience, National Institute on Aging, National Institutes of Health, Baltimore, MD, USA.; ^2^ Laboratory of Cardiovascular Science, National Institute on Aging, National Institutes of Health, Baltimore, MD, USA.; ^3^ Department of Medicine, Brigham and Women’s Hospital, Harvard Medical School, Boston, MA, USA.; ^4^ Department of Neurology, Johns Hopkins University School of Medicine, Baltimore, MD, USA.; ^5^ Institute of Epidemiology and Prevention, Faculty of Medicine and Medical Center, University of Freiburg, Freiburg, Germany.; ^6^ Department of Clinical and Biomedical Sciences, University of Exeter, Exeter, UK.; ^7^ Department of Epidemiology, Johns Hopkins Bloomberg School of Public Health, Baltimore, MD, USA.; ^8^ Icelandic Heart Association, Kopavogur, Iceland.; ^9^ Faculty of Medicine, University of Iceland, Reykjavik, Iceland.; ^10^ Department of Epidemiology, University of North Carolina at Chapel Hill, Chapel Hill, NC, USA.; ^11^ Department of Psychology, University of New Brunswick, Fredericton, New Brunswick, Canada.; ^12^ Centre for Quantitative Medicine, Duke-NUS Medical School, National University of Singapore, Singapore, Singapore.; ^13^ Translational Gerontology Branch, National Institute on Aging, National Institutes of Health, Baltimore, MD, USA.; ^14^ Laboratory of Molecular Biology and Immunology, National Institute on Aging, National Institutes of Health, Baltimore, MD, USA.; ^15^ Laboratory of Genetics and Genomics, National Institute on Aging, National Institutes of Health, Baltimore, MD, USA.; ^16^ Brain Sciences, University College London, London, England, UK.; ^17^ Novartis, Cambridge, MA, USA.; ^18^ Laboratory of Epidemiology and Population Sciences, Intramural Research Program, National Institute on Aging, Bethesda, MD, USA.; ^19^ NEC Solution Innovators Limited, Koto-ku, Tokyo, Japan.; ^20^ FonesLife Corporation, Chuo-ku, Tokyo, Japan.; ^21^ Department of Neurology, Nagoya University Graduate School of Medicine, Nagoya, Aichi, Japan.; ^22^ Department of Clinical Research Education, Nagoya University Graduate School of Medicine, Nagoya, Aichi, Japan.; ^23^ Department of Epidemiology of Aging, National Center for Geriatrics and Gerontology, Obu, Aichi, Japan.; ^24^ Optimal Aging Institute, New York University Grossman School of Medicine, New York, NY, USA.; ^25^ Welch Center for Prevention, Epidemiology, and Clinical Research, Johns Hopkins Bloomberg School of Public Health, Baltimore, MD, USA.; ^26^ Centre for Integrative Biological Signaling Studies (CIBSS), University of Freiburg, Freiburg, Germany.; ^27^ Artificial Intelligence in Biomedical Imaging Laboratory, Perelman School of Medicine, University of Pennsylvania, Philadelphia, PA, USA.; ^28^ Stroke Branch, National Institute of Neurological Disorders and Stroke, Bethesda, MD, USA.; ^29^ Well-being Design Institute for Health, Tohoku University, Aoba-ku, Sendai, Japan.; ^30^ Department of Neurology, University of North Carolina at Chapel Hill, Chapel Hill, NC, USA.; ^31^ Section of Cardiovascular Research, Department of Medicine, Baylor College of Medicine, Houston, TX, USA.; ^32^ Department of Data Science, University of Mississippi Medical Center, Jackson, MS, USA.; ^33^ Department of Biostatistics, Columbia University, New York, NY, USA.

## Abstract

Growth/differentiation factor–15 (GDF15) is a secreted cytokine strongly associated with dementia risk. However, the extent to which GDF15 represents a biomarker and driver of dementia risk remains unclear. Across multiple cohorts, we demonstrated that plasma GDF15 is associated with greater dementia risk over 15- to 25-year follow-up periods when measured in midlife, with stronger associations observed for vascular, compared to Alzheimer’s disease (AD), dementia. Two-sample Mendelian randomization supported plasma GDF15’s mechanistic role in AD and related dementias, while cohort studies linked it to cerebral small vessel disease, neurodegeneration, phosphorylated tau, and a cerebrospinal fluid proteomic signature indicative of neuroimmune activation. Exposure of cultured myeloid cells to recombinant GDF15 altered biological pathways that we subsequently demonstrated are predictive of dementia risk, including interferon/antiviral responses. These findings support circulating GDF15’s role as an early biomarker—particularly for vascular dementia and neuroinflammation—and identify the mechanisms by which it may drive dementia risk.

## Teaser

A blood protein measured in midlife may predict future dementia risk and reveal immune pathways tied to vascular brain damage.

## INTRODUCTION

Pathological changes underlying neurodegeneration and dementia can begin decades before clinical symptoms emerge. For example, amyloid-β (Aβ) accumulation begins for many individuals during midlife, preceding the onset of cognitive deficits in Alzheimer’s disease (AD) by 20 to 30 years (*1*). A recent proteome-wide association study (*2*) identified dysregulation of immune, metabolic, proteostasis, and synaptic proteins in the plasma of middle-aged adults as early as 20 years before dementia onset. Growth/differentiation factor–15 (GDF15)—also known as macrophage inhibitory cytokine–1 (MIC-1) due to its initial classification as an immunosuppressant (*3*)—showed the strongest association with dementia risk; this protein was also associated with accelerated 20-year cognitive decline and YKL-40 levels (a marker of neuroinflammation) in cerebrospinal fluid (CSF) (*2*). Together, these findings suggest that GDF15 may function as an early biomarker for dementia and cognitive decline, as well as an indicator of dysregulated immune processes.

GDF15 is a secreted peptide hormone and cytokine of the transforming growth factor-β superfamily (*4*) that is expressed primarily outside the central nervous system (CNS; e.g., kidney). Along with being a marker of biological aging (*5*), GDF15 has been implicated in a broad range of biological processes, including immune/inflammatory responses (*6*, *7*), energy homeostasis and appetite (*8*), and tumorigenesis (*9*). Elevated circulating levels of GDF15 have been reported in a variety of age-related conditions, ranging from all-cause mortality to end-stage kidney disease (*10*, *11*). Some, but not all, studies have demonstrated that higher circulating GDF15 levels are associated with long-term dementia risk as well as faster rates of cognitive decline (*12*–*14*). While these results indicate that plasma GDF15 may function as an early marker of dementia risk, further investigation is required to (i) determine its predictive value across dementia subtypes and population subgroups, (ii) identify the mechanisms that account for GDF15’s relationship with long-term dementia risk, and (iii) understand how these early molecular changes might be therapeutically targeted to provide further benefits to patients.

**Fig. 1.**
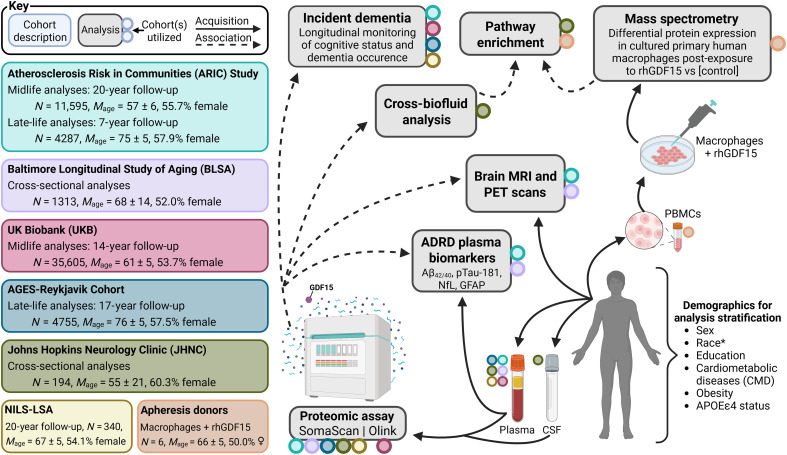
Study design. A multicohort approach was implemented to assess plasma GDF15’s predictive value for, and biological links with, dementia risk and dementia-related endophenotypes. Plasma abundance of GDF15 measured on high-throughput proteomic platforms (SomaScan, Olink) was associated with long-term incident dementia across three independent cohorts, as well as neuroimaging and plasma biomarkers across two independent cohorts. The GDF15-associated CSF proteome and the proteome of human macrophages following in vitro GDF15 experiments were used for pathway analyses. ADRD, Alzheimer’s disease and related dementias; NILS-LSA, National Institute for Longevity Sciences–Longitudinal Study of Aging; PBMCs, peripheral blood mononuclear cells; rhGDF15, recombinant-human GDF15. *Race-stratified analyses were only conducted in the ARIC study due to race heterogeneity among non-white individuals in other cohorts. Created in BioRender. C. Blew (2026) https://BioRender.com/5ecj6lc.

Using high-throughput proteomic data from six cohort studies, we examined the association of plasma GDF15 with all-cause and etiology-specific dementia risk, characterized GDF15’s relationships with dementia-related endophenotypes, and identified the biological processes through which circulating GDF15 may contribute to cognitive decline and dementia (Fig. 1). After replicating the associations of midlife and late-life plasma GDF15 abundance with increased dementia risk and detecting particularly robust associations with vascular dementia (VaD), we found support for plasma GDF15’s mechanistic role in Alzheimer’s disease and related dementias (ADRD) using two-sample Mendelian randomization (MR). Consistent with these findings, we demonstrated that plasma GDF15 was associated with greater cerebral small vessel disease, diffuse neurodegeneration, phosphorylated tau levels in both CSF and plasma, and a CSF proteomic signature indicative of neuroimmune activation. Subsequently, we exposed cultured human macrophages [differentiated from monocytes isolated from peripheral blood mononuclear cells (PBMCs)] to recombinant GDF15, where we identified dysregulated immune and metabolic protein networks that partially mediated associations with dementia risk. Our results suggest plasma GDF15 may function as an early risk factor for dementia risk by modulating metabolic pathways and the neuroimmune axis.

## RESULTS

These analyses were based on six independent cohort studies (table S1), two publicly available genomic datasets, other open-source references, and experimental observations in primary human macrophages (Table 1).

**Table 1.** Cohort and resource application summary. The present analyses involved six independent cohorts and used multiple open-source resources for exploration, identification, and annotation. ACD, all-cause dementia; ADRD, Alzheimer’s disease and related dementias; AGES-Reykjavik, Age, Gene/Environment Susceptibility-Reykjavik Study; Ann., annotation; ARIC, Atherosclerosis Risk in Communities; BLSA, Baltimore Longitudinal Study of Aging; BS, biological sample; C, cohort; EADB, European Alzheimer & Dementia Biobank; ES, etiology-specific; instr., instrument; JHNC, Johns Hopkins Neurology Clinic; NI, neuroimaging; NILS-LSA, National Institute for Longevity Sciences-Longitudinal Study of Aging; OSR, open-source resource; PMID, PubMed Identifier; UKB, UK Biobank.

Dataset	PMID(s)	Type	Proteomics		Dementia				NI	Plasma ADRD bio-markers	Genetics	Cell culture	Ann.
			Plas	CSF	Incident		Prevalent						
					ACD	ES	ACD	ES					
ARIC	2646917	C	✓		✓				✓	✓	✓		
UKB	34737426	C	✓		✓	✓					✓		
AGES-Reykjavik	17351290	C	✓		✓	✓							
BLSA	19126858	C	✓				✓		✓	✓			
NILS-LSA	10835824	C	✓		✓								
JHNC		C	✓	✓									
BioFinder	39187705	OSR		✓		✓*		✓					
deCODE genetics	34857953	OSR									✓		
EADB	35379992 23562540	OSR									✓		
FinGenn	36653562	OSR									✓		
Apheresis donors		BS										✓	
Human Protein Atlas†	32139519	OSR											✓
ONTIME‡	34239129	OSR											✓
Open Targets Genetics§	33045747	OSR											✓
Enrichr¶	27141961	OSR											✓

* The BioFinder cohort was used to analyze protein levels across a pseudotime course of AD pathology.

† Used to quantify protein cell and tissue expression; proteinatlas.org.

‡ ontime.wustl.edu [a resource for single-nucleotide polymorphism (SNP) summary statistics].

§ genetics.opentargets.org (a resource for SNP summary statistics).

¶ Public web tool used for gene set enrichment analysis; maayanlab.cloud/Enrichr.

### GDF15 is broadly expressed outside the CNS and increases over the life span

Before the implementation of analyses, we confirmed the reliability and validity of plasma GDF15 measurements across different proteomic platforms (fig. S1 and table S2); see Materials and Methods for additional details. To determine the origin of circulating GDF15 levels, we leveraged data from the Adult Genotype Tissue Expression Project (GTEx) Portal (*15*) and Human Protein Atlas (*16*). We found that GDF15 is broadly expressed across many cell and tissue types, with the highest expression in the kidney, bladder, and choroid plexus (fig. S2 and table S3). However, there was limited GDF15 expression within CNS tissue or cells.

**Fig. 2.**
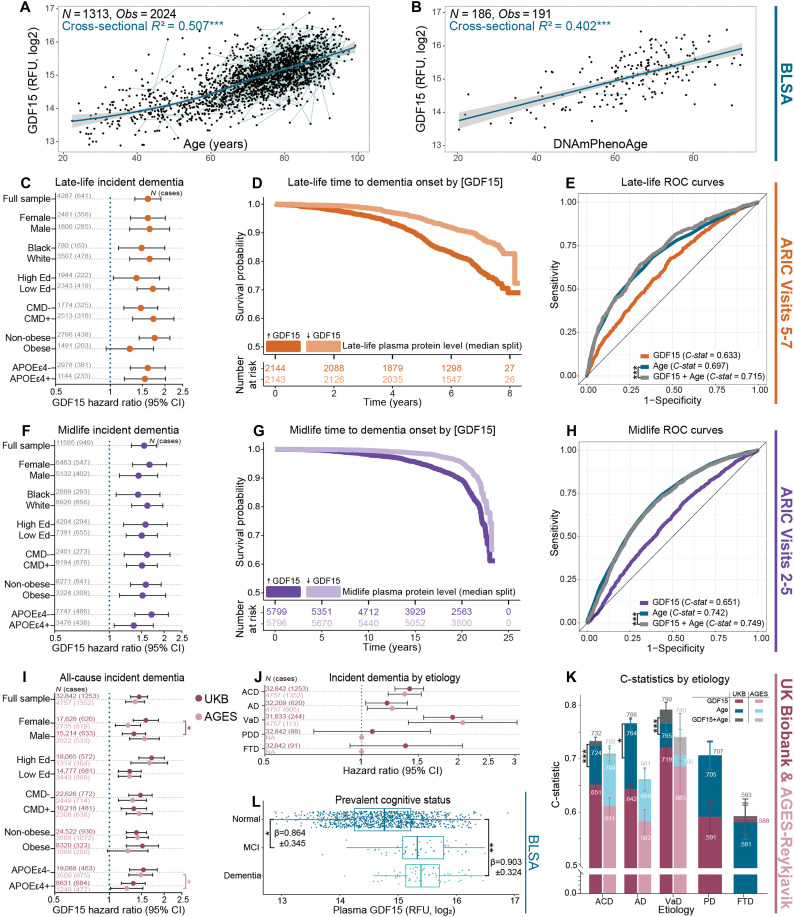
GDF15 abundance in plasma is associated with age and dementia risk across independent cohorts. (**A**) Association of plasma GDF15 abundance and chronologic age in the BLSA. (**B**) Association of plasma GDF15 and an estimation of phenotypic age based on DNA methylation [DNAmPhenoAge (*95*)]. (**C**) Associations of plasma GDF15 (measured in late-life) with 7-year all-cause dementia (ACD) risk in the full ARIC cohort and stratified by dementia risk factors, comorbidities, and demographic characteristics. (**D**) Kaplan-Meier curves showing plasma GDF15 associations (high/low; median split) with ACD-free probabilities across late-life (7 years) in ARIC. (**E**) ROC curves representing the classification of 7-year incident ACD status by late-life GDF15 levels alone, age alone, and GDF15 combined with age. (**F**) Associations of plasma GDF15 (measured in midlife) with 20-year ACD risk in the full ARIC cohort and stratified subgroups. (**G**) Kaplan-Meier curves showing plasma GDF15 associations (high/low; median split) with ACD-free probabilities across midlife (20 years) in ARIC. (**H**) ROC curves representing the classification of 20-year incident ACD status by midlife GDF15 levels alone, age alone, and GDF15 combined with age. (**I**) Associations of plasma GDF15 with ACD risk in the UKB (14-year follow-up) and AGES-Reykjavik (17-year follow-up) cohorts and stratified by dementia risk factors, comorbidities, and demographic characteristics. (**J**) Associations of plasma GDF15 with dementia etiologies in the full UKB and AGES-Reykjavik cohorts (PDD and FTD were not available in AGES). (**K**) *C*-statistics representing plasma GDF15’s predictive performance for etiology specific dementia risk in the UKB and AGES-Reykjavik cohorts. (**L**) Associations of plasma GDF15 with cognitive status in the BLSA. *P < 0.05, **P < 0.01 ***P < 0.001_._ ACD, all-cause dementia; AD, Alzheimer’s disease; ARIC, Atherosclerosis Risk in Communities; BLSA, Baltimore Longitudinal Study on Aging; CMD, cardiometabolic disease; Ed, education; FTD, frontotemporal dementia; PDD, Parkinson’s disease dementia; ROC, receiver operating characteristic; VaD, vascular dementia.

To validate GDF15’s previously reported association with older age (*5*, *17*), we used participant data from the Baltimore Longitudinal Study of Aging (BLSA; *N* = 1313), whose ages ranged from 22 to 93 years old. Here, plasma GDF15 showed a strong, positive cross-sectional association with chronological age (*R*^2^ = 0.507, *P* < 0.001; Fig. 2A). Thus, although GDF15 increases are largely tied to age, approximately half of the variation in circulating GDF15 abundance is independent of age. GDF15 levels were more strongly correlated with age among males (*R*^2^ = 0.56) than among females (*R*^2^ = 0.46; age × sex interaction *P* = 0.005), whereas other factors, such as obesity and *APOE*ε4 status, did not modify GDF15’s association with age (fig. S3). Plasma GDF15 showed a similar association with an epigenetic measure of biological age (DNAmPhenoAge; *N* = 186, *R*^2^ = 0.40, *P* < 0.001; Fig. 2B). However, in analyses restricted to older adults (age 65+), plasma GDF15 levels showed much weaker associations with age [Atherosclerosis Risk in Communities (ARIC), *R*^2^ = 0.086 (fig. S4); BLSA, *R*^2^ = 0.186 (fig. S5)]. In addition to demonstrating that GDF15 expression is not specific to the CNS, these results are consistent with the interpretation that age-related increases in circulating GDF15 may occur more prominently across the life span, rather than being confined to older adulthood.

### Higher plasma GDF15 is strongly associated with dementia risk in multiple large cohorts

To assess the relationships between plasma GDF15 and incident dementia, we analyzed proteomic data from three large cohort studies: ARIC (SomaScan v4.0), the UK Biobank (UKB; Olink Explore), and the AGES-Reykjavik study (SomaScan v4.1). To determine whether GDF15’s associations were modified by demographic characteristics, comorbidities, or established dementia risk factors, we conducted stratified analyses by sex, race, educational attainment [high/low; median split (years)], cardiometabolic disease (pos/neg), obesity [body mass index (BMI) ≥ 30], and *APOE*ε4 carrier status (pos/neg). In addition, we leveraged available data in UKB and AGES to examine etiology-specific dementia risk (e.g., AD, VaD, etc.).

In plasma collected from ARIC participants during late-life (*N* = 4287; mean age: 75.2 ± 5; 58% female; 18% Black), higher GDF15 was associated with increased all-cause dementia (ACD) risk over a 7-year follow-up period [hazard ratio (HR) = 1.61; 95% confidence interval (CI): 1.36, 1.90] after adjustment for demographic factors, physiological variables, and comorbid health conditions (Fig. 2C). In ARIC, ACD cases were clinically adjudicated with established guidelines from the National Institute on Aging (NIA), the Alzheimer’s Association (*18*), and the *Diagnostic and Statistical Manual of Mental Disorders, Fifth Edition* (*DSM-5*) (*19*, *20*). Unadjusted and minimally adjusted analyses yielded similar results (fig. S6A and table S4). In these multivariate analyses, the effect of late-life plasma GDF15 on dementia risk (β = 0.476) was equivalent to 3.75 years of additional age (age β = 0.127). In stratified analyses, the association between GDF15 and ACD risk was consistent across subgroups, except among obese participants, where effects were attenuated (HR = 1.28; 95% CI: 0.95, 1.73). Conversely, the strongest associations between GDF15 and dementia risk were observed in nonobese participants (HR = 1.75; 95% CI: 1.44, 2.13). As illustrated in Fig. 2D, ARIC participants with high plasma GDF15 levels in late-life (median split) showed a doubling of 7-year dementia risk compared to those with low GDF15 levels (18.7% versus 9.5% event rates, respectively; stratified results displayed in fig. S7). With respect to 7-year dementia prediction, plasma GDF15 offered small, but statistically significant, improvements compared to age alone (*C*-statistic of 0.697 versus 0.715; Fig. 2E and table S4). Using a plasma GDF15 cutoff value in late-life that optimizes the balance of sensitivity and specificity for dementia prediction (Youden Index: 15.53 log_2_ RFU), we observed 28.3% sensitivity and 82.0% specificity.

Given the robust associations of plasma GDF15 with late-life ACD risk in older adults, we then examined the association of GDF15 with long-term (20-year) dementia risk using plasma collected from ARIC participants during midlife (*N* = 11,595; mean age: 57.1 ± 6; 56% female, 23% Black). Consistent with our prior results from a later ARIC visit (*2*), each fold increase in midlife GDF15 was associated with a 55% increased risk of ACD risk over a 20-year follow-up (HR = 1.55; 95% CI: 1.32, 1.82; Fig. 2F, fig. S6B, and table S4). These results were maintained when analyses were restricted to participants who had GDF15 measured in early midlife (age < 55; *N* = 4365; HR = 1.57; 95% CI: 1.05, 2.34). Furthermore, midlife plasma GDF15 abundance was associated with ACD risk across all subgroups, with the strongest associations observed among *APOE*ε*4*-negative participants (HR = 1.71; 95% CI: 1.38, 2.11). In these multivariate analyses, the effect of midlife plasma GDF15 on dementia risk (β = 0.439) was equivalent to 3.16 years of additional age (age β = 0.139). ARIC participants with high plasma GDF15 levels in midlife (median split) showed a doubling of 20-year dementia risk compared to those with low GDF15 levels (7.5% versus 3.9% event rates, respectively; Fig. 2G and fig. S8). Compared to age alone, midlife GDF15 in combination with age again offered small, but statistically significant, improvements in predictive performance for 20-year ACD risk (*C*-statistic of 0.742 versus 0.749; Fig. 2H and table S4). Using a plasma GDF15 cutoff value in midlife that optimizes the balance of sensitivity and specificity for dementia prediction (Youden Index: 14.58 log_2_ RFU), we observed 38.9% sensitivity and 84.0% specificity. In a subset of ARIC participants with available ADRD biomarker data (*N* = 1294), plasma GDF15 (HR = 1.82; 95% CI: 1.74, 2.73) showed stronger associations with late-life ACD risk compared to Aβ_42/40_ (HR = 0.81; 95% CI: 0.66, 1.00) and glial fibrillary acidic protein (GFAP; HR = 1.78; 95% CI: 1.43, 2.22), but weaker associations compared to NfL (HR = 2.18; 95% CI: 1.74, 2.73) and pTau-181 (HR = 2.05; 95% CI: 1.74, 2.73; table S4).

As part of a cross-platform validation to identify plasma GDF15’s associations with etiology-specific dementia risk, we next analyzed Olink proteomic data from UKB participants (*N* = 35,673; mean age: 60.7 ± 5; 54% female; 1% non-white). In the UKB, ACD was defined with medical diagnostic codes of all dementia subtypes, including AD, VaD, Parkinson’s disease dementia (PDD), and frontotemporal dementia (FTD). Higher levels of plasma GDF15 were associated with increased ACD risk over a 14-year follow-up period (HR = 1.43; 95% CI: 1.29, 1.59; Fig. 2I and table S5). Although this relationship was maintained in all risk strata, it was significantly stronger in females, compared to males (interaction*-P* = 0.017). In these multivariate analyses, the effect of plasma GDF15 at baseline on ACD risk (β = 0.358) was equivalent to 2.56 years of additional age (age β = 0.140); for etiology-specific age effects, see table S5. With respect to dementia etiology, each log_2_ increase in plasma GDF15 abundance was associated with a 101% higher risk for VaD (HR = 2.01, 95% CI: 1.62, 2.48) and a 20% higher risk for AD (HR = 1.20, 95% CI: 1.03, 1.41) over 14 years. No significant associations were detected with incident PDD or FTD (Fig. 2J). Using a plasma GDF15 cutoff value that optimizes the balance of sensitivity and specificity for ACD prediction (Youden Index: 0.93 log_2_ NPX), we observed 72.1% sensitivity and 49.9% specificity; for etiology-specific Youden Indexes, see table S5. Models that incorporated GDF15 and age significantly outperformed an age only model for prediction of 14-year dementia risk, especially for VaD (*C*-statistic of 0.765 versus 0.790; Fig. 2K, fig. S9, and table S5). Relative to ACD and AD, these results suggest especially elevated risk of VaD among individuals with higher plasma GDF15 levels. Plasma GDF15 showed stronger associations with ACD risk compared to MAPT (HR = 1.19; 95% CI: 1.07, 1.32), but weaker associations than established biomarkers, NfL (HR = 2.21; 95% CI: 2.00, 2.24) and GFAP (HR = 2.33; 95% CI: 2.12, 2.56; table S5).

The association between serum GDF15 and long-term ACD risk (up to 17-year follow-up) was additionally replicated in a group of 4757 participants (mean age: 76 ± 5; 57.5% female) from the AGES-Reykjavik study (HR = 1.36; 95% CI: 1.21, 1.53; Fig. 2I and table S6). In the AGES-Reykjavik cohort, ACD was clinically adjudicated with *DSM-IV* criteria (*21*). In stratified analysis, this association was particularly strong in *APOE*ε4-negative compared to *APOE*ε4-positive participants (interaction*-P* = 0.043; Fig. 2I and table S6). Similarly, GDF15’s relationship with increased dementia risk was consistently stronger among *APOEε4*-negative participants in both the UKB and ARIC, although the moderating effect of *APOE* genotype did not reach statistical significance in these cohorts. Analyses of etiology-specific dementia risk in the AGES-Reykjavik study revealed that each log_2_ increase in serum GDF15 abundance was associated with a 106% higher risk for VaD (HR = 2.06, 95% CI: 1.40, 3.04) and a 24% higher risk for AD (HR = 1.24, 95% CI: 1.04, 1.49) over up to 17 years (Fig. 2J and table S6). Compared to age alone, GDF15 in combination with age was a better predictor of VaD (*C*-statistic of 0.706 versus 0.740), but not AD or ACD (Fig. 2K, fig. S10, and table S6). Meta-analyzed results indicated 44 to 47% increased risk of ACD risk associated with each log_2_ increase in plasma GDF15 (table S7).

Although robust and consistent associations of GDF15 with incident dementia were observed across European and American cohorts, this finding did not extend to a smaller Japanese cohort of 340 participants from the National Institute for Longevity Sciences-Longitudinal Study of Aging (NILS-LSA; *N* = 340; mean age: 66.7 ± 5.2, 54.1% female; HR: 1.19, 95% CI: 0.90, 1.57, *P* = 0.24). In cross-sectional analyses in the BLSA, plasma GDF15 was elevated among those with prevalent MCI (*N* = 50) and dementia (*N* = 63), compared to cognitively normal controls (*N* = 1019; mean age: 69.2 ± 16, 53% female; Fig. 2L). Notably, higher plasma GDF15 was previously associated with worse cognitive functioning across all domains, except memory, in the Generation Scotland cohort study (*22*). Together, these findings indicate that elevated plasma GDF15 levels in midlife or late-life are consistently associated with increased risk of developing dementia, particularly of a presumed vascular etiology (VaD).

### Mendelian randomization postselection inference causally implicates plasma GDF15 in dementia risk

**Fig. 3.**
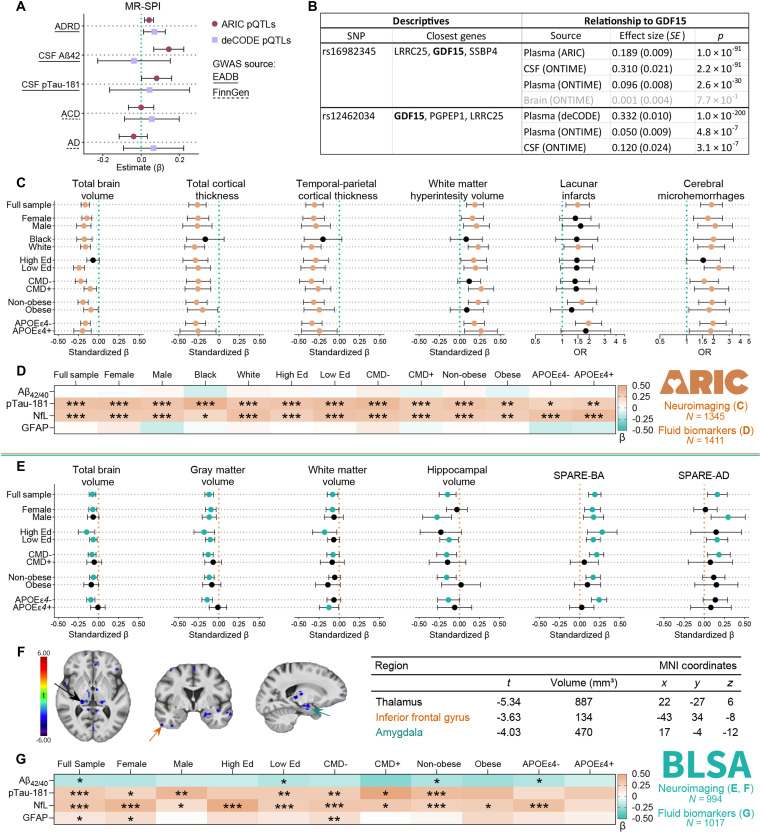
GDF15 abundance in plasma is associated with brain structure and plasma biomarkers across independent cohorts. (**A**) Causal effect estimates were obtained using Mendelian randomization postselection inference (MR-SPI) for clinically defined Alzheimer’s disease (AD) and related dementia (ADRD), all-cause dementia (ACD), cerebrospinal fluid measures of AD pathology, and stroke. (**B**) Top cis-pQTLs from the ARIC and deCODE studies and their association with plasma, CSF, and brain GDF15 abundance in other cohorts. (**C**) Associations of plasma GDF15 with 3-T MRI-derived structural brain imaging in the Atherosclerosis Risk in Communities (ARIC) study cohort and stratification by dementia risk factors, comorbidities, and demographic characteristics. (**D**) Associations of plasma GDF15 with AD biomarkers in the ARIC cohort and stratified by dementia risk factors, comorbidities, and demographic characteristics. ARIC results were obtained from linear regression models adjusted for age, race-center, sex, education, *APOE*ε*4*, eGFR, BMI, diabetes, hypertension, and smoking status; brain volume analyses also adjusted for intracranial volume. (**E**) Associations of plasma GDF15 with 3-T MRI-derived structural brain imaging in the Baltimore Longitudinal Study of Aging (BLSA) and stratification by dementia risk factors, comorbidities, and demographic characteristics. (**F**) Associations of plasma GDF15 with voxel-wise brain volumes in the BLSA. An FDR-corrected threshold of *P* < 0.05 was used to define significant clusters. (**G**) Associations of plasma GDF15 with AD biomarkers in the BLSA cohort and stratification by dementia risk factors, comorbidities, and demographic characteristics. BLSA results were obtained from linear regression models adjusted for age, sex, race, education, *APOEε4*, eGFR, and a comorbidity index; brain volume analyses were also adjusted for intracranial volume. ***P < 0.001; **P < 0.01; *P < 0.05. Aβ_42/40_, amyloid-β 42:40 ratio; CMD, cardiometabolic disease; EADB, European Alzheimer Disease Biobank; Ed, education; GFAP, glial fibrillary acidic protein; NfL, neurofilament light; pTau-181, phosphorylated tau 181.

To assess the potential causal role of GDF15 on ADRD, as well as associated endophenotypes, we used the recently developed Mendelian randomization postselection inference (MR-SPI) approach (*23*). This procedure selects protein quantitative trait loci (pQTLs) as genetic instruments and performs robust causal inference in a two-sample MR framework. For primary analysis, we used plasma GDF15 cis-pQTLs derived from the ARIC cohort (*2*) (*N* = 7597) to proxy lifetime GDF15 exposure. Outcome data were sourced from five genome-wide association study (GWAS) summary statistics: ADRD (*N* = 487,511; AD cases = 39,106; AD proxy cases = 46,828), CSF Aβ42, and CSF pTau-181 (*N* = 13,116) from the European Alzheimer & Dementia Biobank (EADB) consortium (*24*, *25*), and ACD (*N* = 211,397; cases = 7395) and AD (*N* = 111,471; cases = 3899) from the Finnish Biobank (FINBB) (*26*). Our primary analysis revealed significant positive associations of genetically proxied plasma GDF15 abundance with ADRD in the EADB consortium (but not AD in FINBB), CSF Aβ42, and CSF pTau-181 (Fig. 3A and table S8). Using a distinct set of GDF15 plasma cis-pQTLs identified in a large Icelandic study (deCODE) (*27*), we replicated the significant positive association of GDF15 with ADRD in the EADB consortium. However, the associations of GDF15 with CSF Aβ42 and CSF pTau-181 were not replicated. Notably, several of the identified GDF15 plasma pQTLs were also CSF pQTLs, suggesting a genetic coregulation of GDF15 across biofluids (Fig. 3B and table S9).

### Plasma GDF15 is associated with neurodegeneration and cerebral small vessel disease

To characterize the neurobiological links between plasma GDF15 and dementia risk, we examined its associations with structural neuroimaging, fluid biomarkers, and Aβ positron emission tomography (PET). Among ARIC participants (*N* = 1345, mean age: 76.1 ± 5.17, 40.8% female), each fold increase in plasma GDF15 was significantly associated with 0.17 SD lower total brain volume, 0.27 SD lower total cortical thickness, 0.31 SD lower temporal-parietal cortical thickness, and 0.18 SD higher white matter hyperintensity (WMH) volume, as well as 40 and 90% higher odds of at least one lacunar infarct and cerebral microhemorrhage, respectively, in models adjusted for demographic factors, physiological variables, and comorbid health conditions (Fig. 3C). We detected associations of similar magnitude for the hippocampus and a subcortical gray matter meta–region of interest (ROI; table S10).

In an independent sample of cognitively normal participants from the BLSA (*N* = 994, mean age: 66.0 ± 15, 55% female), GDF15 showed similar associations across ROIs (Fig. 3E and table S11) and a diffuse pattern of reduced gray matter volume (especially in the thalamus, amygdala, and inferior temporal gyrus) in voxel-based morphometry (Fig. 3F, fig. S11, and table S12). Each fold-change increase in plasma GDF15 corresponded to a 0.16 SD lower volume in brain regions vulnerable to AD-related atrophy [Spatial Pattern of Abnormality for Recognition of Early Alzheimer’s Disease (SPARE-AD)] and 0.19 SD lower volume in brain regions vulnerable to age-related atrophy [Spatial Pattern of Abnormality for Recognition of Early Brain Aging (SPARE-BA)], a pattern that we subsequently replicated in the UKB (*N* = 2709, mean age: 63.7 ± 8, 52% female; fig. S12 and table S13). An examination of five machine learning–derived predominant patterns of brain atrophy (*28*, *29*) found that elevated plasma GDF15 was significantly associated with a pattern of diffuse cortical atrophy and perisylvian atrophy in BLSA, and a pattern of medial temporal atrophy and perisylvian atrophy in the UKB (fig. S12). Notably, the pattern of perisylvian volume loss associated with GDF15 in both cohorts has been linked to MCI as well as dementia conversion, neuropsychiatric conditions, MS, PD, and an array of other age-related chronic diseases (e.g., reparatory, renal, metabolism, cardiovascular, etc.) (*30*). These results show that higher GDF15 levels in circulation accompany a pattern of enhanced neurodegeneration and cerebral small vessel disease even before the onset of cognitive impairment.

With respect to fluid biomarkers (*31*), higher plasma GDF15 was associated with plasma biomarker evidence of greater tau phosphorylation (pTau-181; standardized β = 0.32, SE = 0.05, uncorrected *P* < 0.001) and neuronal injury (NfL; standardized β = 0.29, SE = 0.04, uncorrected *P* < 0.001), but not soluble amyloid (Aβ_42/40_; standardized β = 0.005, SE = 0.06, uncorrected *P* = 0.927) or reactive astrogliosis (GFAP; standardized β = 0.02, SE = 0.05, uncorrected *P* = 0.704) in adjusted models (ARIC, *N* = 1411; Fig. 3D and table S14). Similar associations of plasma GDF15 with pTau-181 (standardized β = 0.28, SE = 0.08, uncorrected *P* < 0.001) and NfL (standardized β = 0.30, SE = 0.05, uncorrected *P* < 0.001) were observed in cognitively normal BLSA participants (*N* = 690 and 1017, respectively; Fig. 3G and table S15), a finding that extended to CSF pTau-181 (β = 0.43, SE = 0.16, *P* = 0.006) in a sample of 193 participants from the Johns Hopkins Neurology Clinic (JHNC; mean age: 55.3 ± 21, 60.3% female). In contrast, plasma GDF15 was not associated with elevated cortical amyloid, as defined by PET imaging in ARIC (OR = 0.90, 95% CI: 0.50, 1.63; uncorrected *P* = 0.724) or the BLSA (OR = 1.22, 95% CI: 0.48, 3.07; uncorrected *P* = 0.673; table S16).

### Elevated plasma GDF15 is associated with neuroimmune activation in CSF

**Fig. 4.**
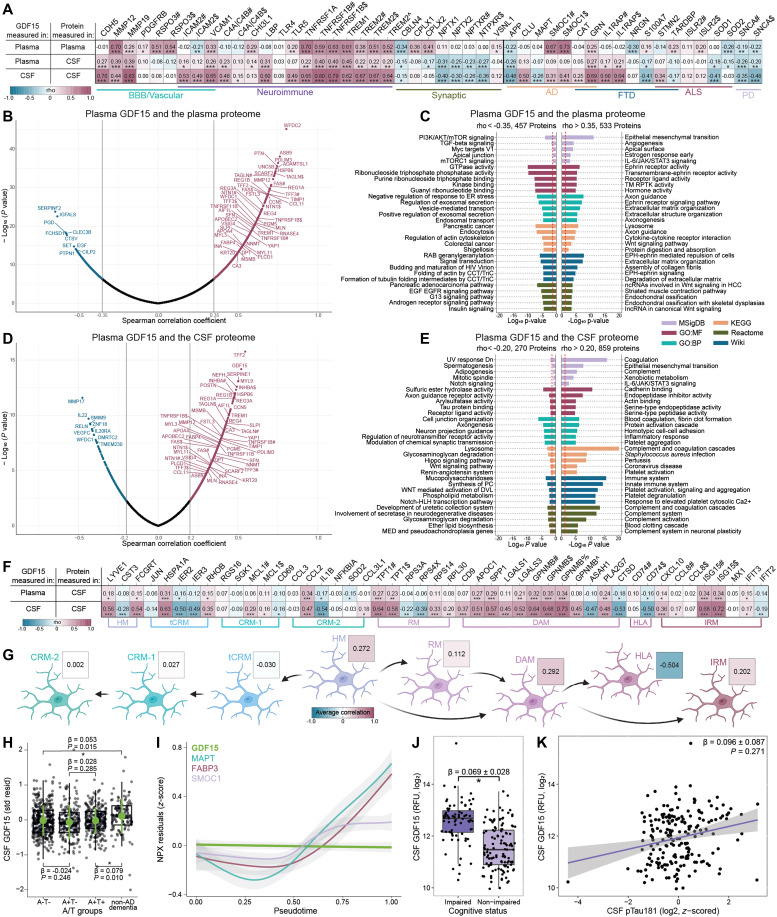
GDF15 relationships across and between biofluids (plasma and CSF). (**A**) Heatmap depicting Spearman correlations between GDF15 and proteins associated with neurodegeneration and related processes (*N* = 193). (**B**) Volcano plot of correlations between plasma GDF15 and the plasma proteome (*N* = 193). (**C**) Enrichment of plasma proteins that showed the strongest negative (left) and positive (right) correlations with plasma GDF15. (**D**) Volcano plot of correlations between plasma GDF15 and the CSF proteome (*N* = 193). (**E**) Enrichment of CSF proteins that showed the strongest negative (left) and positive (right) correlations with plasma GDF15. (**F**) Correlations of plasma and CSF GDF15 with CSF proteins whose cognate genes are differentially expressed in human microglial phenotypes (*N* = 193). (**G**) Diagram depicting average correlations of CSF GDF15 with proteins whose cognate genes were differentially expressed in distinct microglial phenotypes and the direction of phenotypic stage transitions; for duplicate protein SOMAmers, the highest-magnitude correlation was included in averaging. This panel was partly created in BioRender. C. Blew (2026) https://BioRender.com/5ecj6lc. (**H**) Boxplot depicting standardized residuals of CSF GDF15 levels among AD-pathology subtypes (A + |−/T + |−) and non-AD dementia (BioFINDER, *N* = 877). (**I**) Line plot depicting CSF protein level residuals across pseudotime representing the course of AD (BioFINDER, *N* = 877). (**J**) Boxplot depicting levels of CSF GDF15 in cognitively impaired and unimpaired individuals (*N* = 193); results were obtained from logistic regression adjusted for age and sex. (**K**) Scatterplot depicting CSF levels of pTau181 and GDF15 (*N* = 193); results were obtained from linear regression adjusted for age and sex. Unless otherwise specified, analyses were performed in the JHNC cohort (*N* = 193). AD, Alzheimer’s disease; ALS, amyotrophic lateral sclerosis; BBB, blood-brain barrier; CRM, cytokine response microglia; CSF, cerebrospinal fluid; DAM, damage-associated microglia; FTD, frontotemporal dementia; HLA, antigen-presenting response; HM, homeostatic microglia; JHNC; Johns Hopkins Neurology Clinic; IRM, interferon response microglia; PD, Parkinson’s disease; RM, ribosomal microglia; tCRM, transitioning CRM.

Using plasma and CSF proteomic data (SomaScan v4.1) from the JHNC, we found that GDF15 abundance in plasma was strongly correlated with GDF15 abundance in CSF (ρ = 0.52, uncorrected *P* = 6.76 × 10^−5^; fig. S13A). This correspondence between plasma and CSF GDF15 was reduced in the context of dementia, but there was no change in correlation strength with respect to age (fig. S13, B and C). The high plasma-CSF GDF15 correlation is therefore unlikely to be driven by blood-brain barrier breakdown that can accompany increasing age and cognitive impairment. In this same cohort, plasma GDF15 correlated positively with select CSF measures of vascular dysfunction/inflammation [CDH5 (ρ = 0.27), MMP12 (ρ = 0.39), and VCAM1 (ρ = 0.26)] and neuroimmune activation [TNFRSF1B (ρ = 0.40), TREM1 (ρ = 0.45), TREM2 (ρ = 0.22), and LBP (ρ = 0.31); all uncorrected *P* < 0.05; Fig. 4A and table S17]. CSF GDF15 showed a similar pattern of significant associations, albeit at a magnitude that was twice that of the plasma-to-CSF associations [e.g., VCAM1 (ρ = 0.65), TNFRSF1B (ρ = 0.80), TREM2 (ρ = 0.67); all uncorrected *P* < 0.05]. In a proteome-wide analysis of approximately 7000 proteins (SomaScan v4.1; uncorrected *P* < 0.05), higher plasma GDF15 was associated with an altered abundance of plasma proteins involved in immune processes (e.g., IL-6/JAK/STAT3 signaling), ephrin receptor signaling, and extracellular matrix organization (Fig. 4, B and C, and table S18), as well as an altered abundance of CSF proteins involved in complement activation and coagulation, IL-6/JAK/STAT3 signaling, *Staphylococcus aureus*, pertussis, and coronavirus infections, in addition to an attenuation in neuronal viability pathways (Fig. 4, D and E, and table S18).

Given our results linking elevations in plasma GDF15 to immune pathways implicated in neuroinflammation (e.g., STAT3 and complement), we then asked whether GDF15’s CSF proteomic signature aligned with expression patterns obtained from single-cell sequencing of different human microglia populations (*32*). Specifically, we assessed the extent to which plasma and CSF GDF15 abundance correlated with CSF markers of homeostatic (HM), cytokine response (tCRM, CRM-1, and CRM-2), ribosomal (RM), disease-associated (DAM), human leukocyte antigen (HLA), and interferon response (IRM) microglia subtypes (Fig. 4F and table S18). Plasma and CSF GDF15 showed strong correlations (all uncorrected *P* < 0.05) with several CSF proteins primarily expressed by CRM (HSPA1A and CCL2), DAM (APOC1, SPP1, and GPNMB), and IRM (ISG15). Compared to plasma GDF15, intrathecal GDF15 was more strongly associated with these CSF markers of microglia subtypes, presumably due to the CNS specificity of genes that differentiate microglia populations. By averaging GDF15 correlations within specific microglial subpopulations, we found that CSF GDF15 was most strongly related to CSF proteins implicated in the transition of microglia from a homeostatic to damage-associated and interferon-responsive state (HM-DAM-IRM pathway; Fig. 4G).

### CSF GDF15 is elevated in the context of non-AD dementia and cerebral small vessel disease

To shed additional light on the role of CSF GDF15 in ADRD, we determined the extent to which CSF GDF15 is associated with biomarker-defined AD dementia, non-AD dementia, and cerebral small vessel disease using published data from the BioFINDER-2 cohort (*24*) and additional data from the JHNC. After adjusting for age, sex, and mean overall protein level, CSF GDF15 was not elevated among Aβ-positive/tau-negative (A+T−) participants compared to those without AD pathology (A−T−), or in Aβ-positive/tau-positive (A+T+) participants compared to Aβ-positive/tau-negative (A+T−) participants. However, CSF GDF15 was elevated among participants with non-AD dementia when compared to (i) participants with AD pathology (A+T+) and (ii) cognitively normal participants without AD pathology (A−T−; Fig. 4H). In a pseudotime analysis that used CSF Aβ and tau to assign each BioFINDER participant along a continuum on the hypothesized AD course (*24*), CSF GDF15 levels remained relatively constant, particularly in comparison to proteins that are known to increase with Aβ (A+/T−; SMOC1, MAPT) and tau (A+/T+; MAPT, FABP3; Fig. 4I). Consistent with these results, CSF GDF15 was elevated in cognitively impaired (MCI or dementia), compared to cognitively unimpaired JHNC participants (β = 0.07; SE = 0.03; uncorrected *P* = 0.02; Fig. 4J), but was not associated with a CSF marker of phosphorylated tau (pTau-181; β = 0.10, SE = 0.09; uncorrected *P* = 0.27; Fig. 4K). CSF GDF15 has also been strongly associated with magnetic resonance imaging (MRI) indicators of cerebral small vessel disease, including higher WMH volume (β = 0.09; SE = 0.02; uncorrected *P* = 2.93 × 10^−7^) and lobar microbleeds (β = 0.04; SE = 0.02; uncorrected *P* = 0.02), in published results from the BioFINDER-2 cohort (*APO*ε4 unadjusted) (*33*). These associations of CSF GDF15 with cerebral small vessel disease but not AD pathology align with our results, suggesting that GDF15 is more sensitive to VaD than to AD.

### GDF15 modulates innate immune function and alters the human macrophage proteome in vitro

While the preceding analyses highlight strong associations between GDF15 and neuroimmune activity within the CNS, GDF15 itself is not enriched in CNS tissues and is primarily secreted into circulation. This raises the possibility that its influence on CNS pathology occurs indirectly—perhaps via effects on peripheral immune cells. To test this, we leveraged genetic and experimental approaches to assess whether GDF15 modulates innate immune function in the periphery.

**Fig. 5.**
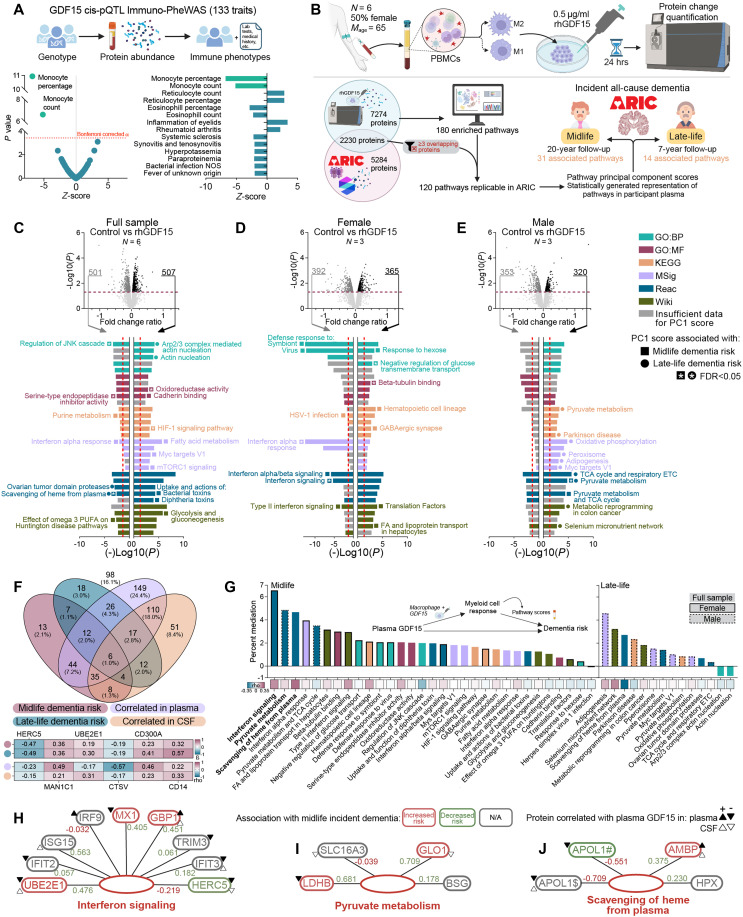
Probing GDF15 biology in primary human macrophages and translating results to clinical outcomes. (**A**) IPheWAS examining the relationship between genetically imputed GDF15 and 133 immune-related phenotypes. (**B**) Top schematic depicts the experimental process of exposing PBMC-derived macrophages to rhGDF15. Bottom schematic depicts the analytic process of relating protein mass spectrometry results postexposure to rhGDF15 to dementia risk in ARIC participants via pathway PC1 score representations. (**C**) Volcano plot of protein changes in PBMC-derived macrophages exposed to rhGDF15 (compared to controls); enrichment analyses using significantly altered proteins (*P* < 0.05) are reflected in the corresponding bar charts. Analyses were repeated in (**D**) females only and (**E**) males only. Labeled pathways were associated with incident dementia in ARIC (midlife, 20-year follow-up; late-life, 7-year follow-up) using adjusted Cox proportional hazards. (**F**) Venn diagram depicting the number of proteins associated with rhGDF15 exposure in at least one of the prior analysis groups (A to C) and how they related to dementia risk (ARIC) and correlated with plasma GDF15 when measured in plasma and CSF (JHNC). The middle six proteins were significantly related to mid- and late-life dementia risk and correlated with plasma GDF15 when measured in both plasma and CSF; their association with each outcome is displayed in the heatmap below. (**G**) Bar chart depicting the extent to which pathway component scores were estimated to mediate the association between plasma GDF15 and incident dementia in ARIC. (**H** to **J**). Schematics depicting how proteins within the interferon signaling, pyruvate metabolism, and scavenging of heme from plasma pathways (respectively) related to midlife dementia risk and load onto PC1 scores (ARIC). ARIC, Arthrosclerosis Risk in Communities; IPheWAS, immuno-phenome-wide association study; PBMCs, peripheral blood mononuclear cells; PC1, first principal component; rhGDF15, recombinant human GDF15. Schematics within (A) and (B) and (G) to (J) were created in BioRender. C. Blew (2026) https://BioRender.com/5ecj6lc.

Using GDF15 cis-pQTLs identified in ARIC and a two-sample MR framework (*34*, *35*), we first examined relationships between genetically determined plasma GDF15 levels and 133 immune traits (table S19). This immuno-phenome-wide association study (IPheWAS) supported the causal relationship between elevated GDF15 and lower monocyte count (*Z* = −6.78; uncorrected *P* = 1.14 × 10^−11^) and lower monocyte percentage (*Z* = −5.17; uncorrected *P* = 2.37 × 10^−7^), after Bonferroni correction (Fig. 5A). These findings provided genetic support for GDF15’s inhibitory effect on innate immune processes.

Next, we determined how the manipulation of extracellular GDF15 protein levels (akin to altered GDF15 abundance in circulation) affects the proteomic profiles and biological processes of cultured human immune cells. Using macrophages differentiated from monocytes that were isolated from human PBMCs from six donors (mean age: 66.3 ± 5; 50% female) in conjunction with mass spectrometry-based proteomics applied to cell lysates, we identified differentially abundant proteins following exposure to recombinant human GDF15 (rhGDF15; Fig. 5B and table S20). Full sample and sex-stratified analyses were conducted because of the unique proteomic variance between male and female donors (fig. S14). A total of 610 proteins were significantly (uncorrected *P* < 0.05) influenced by treatment with rhGDF15 in the full sample, females only, and/or males only. We conducted enrichment analyses on these differentially abundant proteins, identifying an elevation of proteins associated with pathogen exposure (e.g., bacterial toxins) and metabolic pathways (e.g., glycolysis and glucogenesis, mTORC1 signaling) and a reduction of proteins linked to interferon response and iron transport pathways (Fig. 5C, fig. S15A, and table S21). In sex-stratified enrichment, we saw further attenuation of immune pathways—particularly interferon signaling—in females (Fig. 5D, fig. S15B, and table S21) and additional amplification of metabolic processes in males (Fig. 5E, fig. S15C, and table S21). Of the proteins modulated by rhGDF15 exposure, 29 were associated with future dementia risk when measured during midlife and during late-life in ARIC, including six (HERC5, MAN1C1, UBE2E1, CTSV, CD300A, and CD14) that showed significant correlations with plasma GDF15 when measured in both CSF and plasma in the JHNC (Fig. 5F). Our analyses revealed that HERC5, an interferon-induced E3 protein ligase (*26*), accounted for 4% of the observed statistical association between midlife GDF15 and 20-year dementia risk, whereas CD300A, an inhibitory immune receptor found on both myeloid and lymphoid cells (*36*), accounted for 6% of the relationship between late-life GDF15 and 7-year dementia risk (table S22). Given their consistent relationships with dementia risk and GDF15 levels across biofluids, these two proteins may play particularly important roles in mediating the biological mechanisms by which GDF15 influences dementia risk.

### Translating observed cell culture responses to dementia risk

To determine how plasma GDF15’s interactions with innate immune cells may influence dementia risk, we derived biologically informed composite (principal component) scores using proteins annotated in rhGDF15-macrophage–enriched pathways. Then, we determined the extent to which each pathway was associated with incident dementia over a 20- and 7-year follow-up (ARIC; *N* = 11,595 and *N* = 4287, respectively; Fig. 5B and table S23). Out of 180 rhGDF15-macrophage–enriched pathways identified in our preceding experiments in culture (i.e., top five up- and down-regulated pathways identified with six public databases from the full sample, females only, and males only), we were able to recapitulate 120 in ARIC using available plasma proteins measured by the SomaScan assay. Of these, 31 and 14 were associated with dementia risk when measured during midlife and late-life, respectively, in covariate-adjusted analyses (unadjusted *P* < 0.05; Fig. 5, C to E). Dementia-associated, rhGDF15-macrophage–enriched pathways included a diverse set of immune (particularly interferon and viral/antiviral) and metabolic processes, the former being more prominent in female participants, and the latter in male participants.

**Fig. 6.**
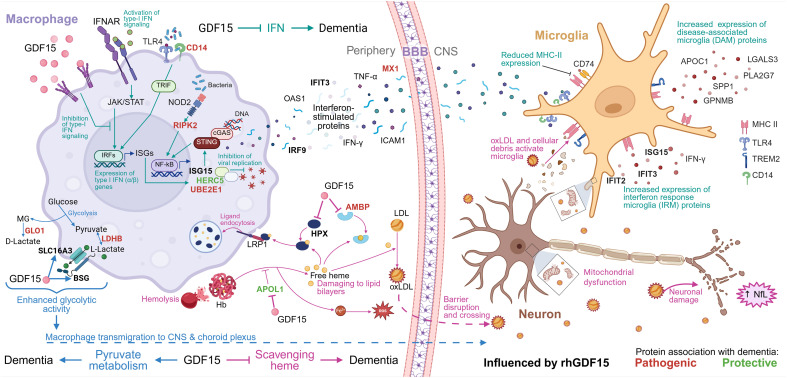
Proposed mechanisms linking GDF15 to dementia risk. Proposed mechanisms of how pathways influenced by GDF15 exposure in macrophages [interferon response (teal), pyruvate metabolism (blue), and scavenging of heme from plasma (pink)] may promote or protect against dementia risk. Created in BioRender. C. Blew (2026) https://BioRender.com/5ecj6lc.

Of particular interest were three rhGDF15-enriched pathways associated with higher 20-year dementia risk when assessed at midlife [false discovery rate (FDR) < 0.05]. These pathways—interferon signaling, pyruvate metabolism, and scavenging of heme from plasma—were identified as the strongest biological mediators of GDF15’s relationship with dementia risk (6.6, 4.9, and 4.7 mediation, respectively; Fig. 5G and table S24). The interferon response pathway was down-regulated in macrophages following exposure to rhGDF15 and positively associated with dementia risk when measured during midlife, as were three of the nine proteins annotated in this pathway (Fig. 5H). These findings are consistent with an immunosuppressive role for GDF15, particularly in relation to antiviral signaling, and suggest that plasma GDF15’s association with dementia risk may involve its influence on myeloid cell interferon signaling. On the other hand, proteins involved in pyruvate metabolism were strongly up-regulated in macrophages following exposure to rhGDF15 (particularly among male participants) and associated with elevated dementia risk when measured during midlife and late-life (Fig. 5I). Proteins in this metabolic pathway—including glyoxalase I and lactate dehydrogenase B—modulate lactate/pyruvate levels and energy production, an excess of which can promote macrophage infiltration into the perivascular regions of the CNS (e.g., choroid plexus), where they can then respond to pathogens and other damage-associated molecular patterns (*37*). Proteins involved in the scavenging free heme (a cytotoxic by-product of hemolysis) from plasma were down-regulated in macrophages exposed to rhGDF15 and associated with dementia risk when measured during midlife and late-life (Fig. 5J). By suppressing a network of heme scavenging proteins, such as APOL1 and AMBP, circulating GDF15 may promote extracellular increases in free heme, which can catalyze the formation of reactive molecules that cause neuronal damage, such as reactive oxygen species (*38*, *39*) and oxidized low-density lipoproteins (LDLs) (*40*) (Fig. 6).

## DISCUSSION

Leveraging data from six independent cohorts, we found that elevated GDF15 in plasma—measured in both midlife and late-life—is strongly associated with future dementia risk. This association was particularly pronounced for VaD, where effect sizes were approximately two to five times greater than for AD, suggesting that GDF15 may serve as a marker of vascular cognitive impairment. These findings were genetically supported, as MR analyses suggested a causal link between greater plasma GDF15 abundance and increased ADRD risk. Plasma GDF15 was also associated with neuroimaging indicators of diffuse neurodegeneration and cerebral small vessel disease, as well as increased phosphorylated tau (pTau-181, in both plasma and CSF) and neurofilament light—even among cognitively unimpaired individuals. In contrast, GDF15 was not associated with amyloid pathology (as measured by PET cortical amyloid or plasma Aβ_42/40_) and did not increase in CSF with progressive AD pathological staging. Individuals with higher plasma GDF15 exhibited proteomic profiles in both plasma and CSF indicative of immune and metabolic perturbations. These molecular changes were consistent with results from our cell culture experiments, where rhGDF15 exposure altered expression of proteins involved in interferon signaling, pyruvate metabolism, and heme scavenging in primary human macrophages. By linking these pathways to dementia risk, our findings suggest potential biological processes through which GDF15 may influence dementia pathogenesis.

Although the association of plasma GDF15 with dementia risk has been reported previously (*2*, *12*, *17*), its utility as a biomarker and potential mechanistic role in brain aging and neurodegenerative disease remain poorly understood. Our findings extend existing evidence by demonstrating that elevated GDF15 levels are detectable in midlife—before age 55—in individuals who later develop dementia. This observation, which is built upon prior work by our group (*2*), was replicated here using an earlier (i.e., younger) wave of the ARIC cohort. GDF15 was associated with dementia risk in nearly all demographic and clinical subgroups, with a tendency for stronger associations among *APOE*ε*4* noncarriers compared to *APOEε4* carriers. This pattern is consistent with plasma GDF15’s (i) stronger association with VaD than with AD, (ii) robust links to small vessel disease on MRI, and (iii) lack of association with core amyloid biomarkers. However, although we formally tested for an interaction between age and *APOE*ε*4* status on GDF15 levels and found no evidence of effect modification, we did not conduct analyses stratified by discrete age categories. Therefore, we cannot exclude the possibility that the association between GDF15 and age differs by *APOE*ε*4* in advanced age groups (e.g., >85 years).

Notably, the association between GDF15 and ACD risk was robust in American and European cohorts but was not observed in the Japanese cohort. Although ancestry-specific biological differences in GDF15 signaling or its relationship to age-related outcomes have not been well characterized, prior studies report substantially lower circulating GDF15 concentrations among individuals of East Asian ancestry compared with white and Black individuals (*41*, *42*). These differences—and the possibility of ancestry-specific genetic or environmental modifiers—underscore the need for future multiancestry studies to better understand population differences in GDF15 biology and its utility as a biomarker. While MR analyses replicated a putative causal link between GDF15 and dementia risk, the overall evidence suggests that this relationship is unlikely to be driven by Aβ pathology. Rather, our results point toward immune-mediated mechanisms—potentially shared across dementia subtypes—as more plausible mediators. The strong positive association between plasma GDF15 and soluble tau (pTau-181) in both plasma and in CSF raises the possibility that GDF15 may contribute to early tau pathology through downstream immune mechanisms. Unfortunately, pTau-217 was not measured in these cohorts, and we acknowledge that its inclusion could have provided additional specificity for AD pathology (*43*).

By conducting an in-depth characterization of GDF15’s CSF proteomic signature, the current study sheds light on potential CNS mechanisms linking it to neurodegeneration. We observed a strong correlation between plasma and CSF GDF15 levels, which is consistent with the possibility that circulating GDF15 crosses into the CNS. Potential routes for this transfer include the choroid plexus—where GDF15 expression is relatively high—or through alternative mechanisms like passive diffusion or active transport across the blood-brain barrier. Elevated plasma GDF15 was associated not only with CSF GDF15 but also with molecular signatures of neuroimmune activation in CSF. Differentially expressed proteins in the CSF of individuals with increased plasma GDF15 were enriched for pathways related to complement and coagulation systems, IL6/JAK/STAT3 signaling, and infection responses. Further support for this link between plasma GDF15 and neuroimmune activation comes from our results, which showed that GDF15 was associated with CSF levels of TREM2 and other proteins highly expressed by disease-associated (e.g., APOC1, SPP1, and GPNMB) and interferon response (e.g., ISG15) microglial populations. CSF GDF15, elevated in non-AD dementia, was more strongly associated with proteins expressed by DAM and IRM compared to plasma GDF15. GDF15’s strong correlation with TREM2 suggests that GDF15 is associated with microglia-mediated immune response in the context of neurodegenerative disease. Although TREM2 has been identified as a risk gene for AD, the TREM2 receptor responds to a variety of pathology and lipids (damage-associated molecular patterns) not specific to AD, including myelin debris (*44*, *45*). *APOE*-dependent activation of TREM2 microglial signaling appears to be necessary for effective clearance of such pathology, and this response has been shown to be attenuated among *APOE*ε*4* carriers (*44*, *46*). Although speculative, our results that show stronger GDF15-dementia associations among *APOE*ε*4* noncarriers may reflect a stronger link between GDF15 and TREM2-mediated DAM activation among *APOE*ε*4* noncarriers (*47*). GDF15 also showed a notable inverse association with C74, primarily expressed by antigen-presenting (HLA positive) microglia. These findings suggest that elevated CSF GDF15 co-occurs with specific microglial activation states responsive to damage and pathogens, potentially through mechanisms independent of GDF15’s canonical CNS receptor, GFRAL. While these findings suggest coordinated systemic and CNS immune cross-talk, further studies are needed to definitively determine how plasma GDF15 and damage-responsive CSF immune signatures are related despite the protein’s minimal expression in brain tissue and cell types.

Circulating GDF15 abundance correlates with molecular changes that can be protective in some contexts—such as suppressing cancer cell proliferation (*48*, *49*); however, our cohort analyses and two-sample MR support the idea that, in dementia, GDF15’s immunosuppressive effects may instead contribute to a pathogenic process. Thus, despite its immunosuppressive role, GDF15 appears to increase dementia risk rather than mitigate it. Using primary human macrophages, we identified multiple biologically relevant protein networks and pathways that may mediate the dementia promoting effects of elevated plasma GDF15. On the basis of the magnitude of these mediation effects, we prioritized investigation of interferon signaling, pyruvate metabolism, and heme scavenging pathways. Our genetic and experimental evidence supports an immunosuppressive role for GDF15, leading us to hypothesize that GDF15 levels increase in response to interferon activity as a feedback mechanism to attenuate or resolve the innate antiviral response. In this framework, either peripheral interferon-driven immune activation is protective—wherein GDF15-mediated immunosuppression inadvertently increases dementia risk—or peripheral interferon signaling is pathogenic and GDF15 is a by-product that influences dementia through alternative mechanisms. One such potential mechanism involves the scavenging of heme from plasma, which was down-regulated by rhGDF15 and associated with increased dementia risk in our analyses. This effect appears partly driven by the decrease in APOL1, a protein that helps prevent the creation of free heme following hemolysis, upon exposure to GDF15. Excess free heme is damaging to lipid bilayers and promotes the oxidization of LDLs. While intact LDLs typically do not cross the blood-brain barrier, oxidized LDLs may do so, partly by contributing to barrier disruption (*50*), giving rise to neuronal injury (*51*). Disruption in heme scavenging could contribute to neurovascular damage and oxidative stress, both of which are implicated in vascular contributions to cognitive impairment and dementia.

This study has several notable strengths, including the integration of data from multiple independent cohorts with up to 20 years of dementia follow-up, the application of high-throughput proteomic data in both plasma and CSF, the incorporation of multimodal neuroimaging, and the use of primary human immune cell cultures to experimentally probe GDF15’s effect. However, several limitations warrant consideration. First, because of the limited availability of CSF measurements across large cohorts with long-term follow-up, we could not assess if CSF GDF15 levels might serve as a stronger predictor of dementia risk than plasma levels. Second, while our use of primary human macrophages provided important insights into GDF15’s modulation of the innate immune response, we did not directly examine microglia—the brain’s resident immune cells—and thus cannot fully extrapolate these findings to neuroimmune mechanisms within the CNS. In addition, primary human macrophages were collected from anonymous volunteers who did not report genetic information; because *APOE*ε*4* can modulate immunological and metabolic responses (*52*), it is possible that the in vitro proteomic signatures we derived may be confounded by this AD risk variant. Third, differences between proteomic platforms—mass spectrometry for cell culture samples and SomaScan aptamer-based assays for plasma—may have introduced measurement discordance, limiting direct comparability between in vitro and cohort data. Last, although we evaluated *APOE*ε*4* status as a potential effect modifier, we did not assess whether associations between GDF15 and dementia-related outcomes vary jointly by age and *APOE* genotype. We acknowledge, however, that age-dependent effects of *APOE* on brain structure and dementia risk have been demonstrated (*53*, *54*) and that these two variables may interact with other putative risk factors, such as GDF15, to affect brain health and dementia risk. Despite these limitations, our findings advance understanding of GDF15 as a promising biomarker for cognitive decline and shed light on biological pathways through which GDF15 may contribute to dementia risk.

## MATERIALS AND METHODS

### Study design

We investigated how GDF15 protein levels in plasma relate to incident dementia and other endophenotypes of neurodegeneration via proteomic, neuroimaging, and genetic techniques. A multicohort study design (cohorts detailed below) was implemented to identify relationships of circulating GDF15 with incident dementia risk (ARIC, UKB, AGES-Reykjavik study, and NILS-LSA), neuroimaging outcomes (ARIC and BLSA), plasma biomarkers (ARIC and BLSA), and the plasma and CSF proteome (JHNC). We also examined whether demographic characteristics (e.g., self-reported sex) and other risk factors (e.g., presence of cardiometabolic disease) moderated these associations, applied genetic inference techniques, and used cell culture conditions to determine the effects of manipulating GDF15 abundance. FDR was used for multiple comparison correction. A detailed study design and flowchart is provided in Fig. 1.

### Validation of plasma GDF15 measurements

We first confirmed the reliability of plasma GDF15 measurements on the SomaScan platform using previously reported results from independent cohorts. In a subsample of participants from the ARIC study, SomaScan GDF15 measurements were strongly correlated with GDF15 measurements obtained from a proximity extension assay (Olink: *r* = 0.79, *P* < 0.0001, *N* = 427) and two targeted immunoassays (Roche: *r* = 0.92, *P* < 0.0001, *N* = 110; Luminex: *r* = 0.83, *P* < 0.0001, *N* = 110) (*55*) (fig. S1 and table S2). These findings are consistent with other large cohort studies that have confirmed the reliability of SomaScan GDF15 measurements using another immunoassay (R&D Systems) (*56*) and proximity extension assays (*57*–*59*) (table S2).

### The ARIC study

#### Population

ARIC is an ongoing, community-based study that initially enrolled 15,792 mostly white and Black participants from four communities within the United States (Jackson, MS; northwestern suburbs of Minneapolis, MN; Forsyth County, NC; and Washington County, MD) between 1987 and 1989 (*60*). Until visit 4 (1996 to 1998), participants were evaluated every 3 years at in-person study visits. Visit 5 was then conducted in-person 15 years later (2011 to 2013), visit 6 took place about 5 years after (2016 to 2017), and visit 7 occurred about 2 years later (2018 to 2019). Data collection for subsequent ARIC study visits is ongoing. Non-white or non-Black participants were excluded, along with Black participants from Minneapolis and Washington Counties due to low sample sizes. Participants missing essential covariates, SomaScan protein measurements, or with a dementia diagnosis on or before the study’s baseline visit (visit 2 for midlife analyses and visit 5 for late-life analyses) were also excluded. Blood was drawn for proteomic analysis at visits 2 and 5. Midlife dementia risk was assessed between visits 2 and 5, and late-life dementia risk was assessed between visits 5 and 7. The neuroimaging and plasma biomarker data used in the present study were collected at visit 5. Institutional review boards (IRB) approved the study protocols at each participating center: Johns Hopkins University, Baltimore, MD; University of Mississippi Medical Center, Jackson, MS; University of North Carolina at Chapel Hill, NC; and Wake Forest University, Winston-Salem, NC (IRB00311861). All participants gave written informed consent at each study visit, and proxies provided consent for participants who were judged to lack capacity.

#### Plasma proteomics

Protein measurements were derived from blood samples collected at ARIC visits 2 and 5 using the SOMAmer-based array method (SomaScan v4.0 platform), as described previously (*61*). Plasma was collected using standardized protocols and frozen at −80°C until analysis. Using blind duplicates, the intra-assay coefficient of variation (CV) for GDF15 was 8.9% for visit 2 (*N* = 520) and 10.7% for visit 5 (*N* = 204). Because of skewed distributions among some protein measurements, all protein values were log_2_ transformed and those beyond 5 SDs were winsorized. Plasma collected at visit 5 was also used to validate SomaScan GDF15 quantification using an enzyme-linked immunosorbent assay, as described previously (*55*).

#### Targeted plasma biomarkers

Aβ_40_, Aβ_42_, GFAP, and NfL concentrations were measured using the Single Molecule Array (Simoa) Neurology 4-Plex E (N4PE). pTau-181 was measured using Simoa HD-X instrument (Quanterix) assays. Using 90 blind duplicates, CVs were 8.5, 7.3, 11.3, 9.8 and 9.7% for Aβ_40_, Aβ_42_, GFAP, NfL, and pTau-181, respectively. Values for GFAP, NfL, and pTau-181 were log_2_ transformed to correct for skewness. Aβ_42/40_ ratio and standardized values were used in analyses, and those beyond 5 SDs were winsorized. Biomarkers used in the present study were acquired at visit 5.

#### Dementia assessment

*Late-life analysis (visits 5 to 7).* At visits 5, 6, and 7, participants underwent comprehensive cognitive and functional in-person assessments to quantify memory, language, processing speed, and executive function, as described previously (*20*). Between these visits, a phone surveillance approach was implemented, where participants were administered the Six Item Screener (SIS), a brief cognitive assessment, annually (*62*). If the participant received a low score on the SIS (or was not able to participate in the screening via phone), the Ascertain Dementia 8 (*63*) was administered to the participant’s informant. For participants who attended visits 6 and 7, these measures were used to estimate the date of dementia onset. For participants who did not attend visits 6 and 7 (due to death or nonattendance), these measures along with hospital discharge codes and death certificate codes were used to define dementia diagnoses and date of dementia onset (*64*). An algorithmic dementia diagnosis was initially defined when the following criteria were met: a score >5 on the Functional Activities Questionnaire (FAQ) or a Clinical Dementia Rating scale sum of boxes >3; two or more cognitive domain scores >1.5 × SD below the normative mean; and previous evidence of decline on the cognitive battery of >0.055 × SD per year, which approximates the rate of cognitive decline in cognitively healthy older adults (*65*, *66*). All dementia diagnoses identified using the algorithm were confirmed by an expert committee of physicians and neuropsychologists based on diagnostic criteria from the NIA, the Alzheimer’s Association (*18*), and the *DSM-5* (*19*, *20*).

*Mid-life analysis (visits 2 to 5).* Dementia surveillance methodology for ARIC visits 1 through 5 has been detailed previously (*20*, *67*). At visit 2, the baseline visit for the present analysis, and visit 4, participants were administered three neurocognitive tests (delayed word recall, digit symbol substitution, and word fluency test). After visit 2, telephone follow-ups were conducted annually. For a subset of participants suspected of having dementia, modified versions of the CDR and the FAQ were administered to informants. For participants who attended visit 5, these measures were used to estimate the date of dementia onset. For participants who did not attend visit 5 (due to death or nonattendance), the TICSm, CDR, FAQ, hospital discharge codes, and death certificate codes were used to define dementia diagnosis and date of dementia onset.

#### Brain MRI

Structural brain images were acquired using 3-T MRI scanners [Siemens Verio (Maryland study center), Siemens Skyra (North Carolina study center), Siemens Trio (Minnesota study center), and Siemens Skyra (Mississippi study center)]. T1-MPRAGE (magnetization-prepared rapid gradient echo) and T2-FLAIR (T2-weighted-fluid-attenuated inversion recovery) scans were acquired for all participants. Details of MRI acquisition have been described previously (*68*). T1-weighted scans were used to estimate brain volumes; ROI volumes were quantified using FreeSurfer software (*69*, *70*). In this study, the following neuroimaging measures were examined: total brain volume, total and temporal-parietal meta-ROI cortical thickness, subcortical gray matter meta-ROI volume, hippocampal volume, WMH volume, lacunar infarcts, and total, lobar, and subcortical cerebral microhemorrhages. WMH volume and lacunar infarcts were quantified from T2 FLAIR images using a computer-aided segmentation program (FLAIR-histoseg) (*71*, *72*); WMH volumes were log transformed. Cerebral microhemorrhages were identified from T2* Gradient Echo images.

#### Amyloid PET imaging

^18^F-florbetapir PET (20 min) scans were acquired as described previously (*73*). Scans were conducted (Siemens) 50 to 70 min after an intravenous bolus injection of the radiotracer. Standardized uptake value ratios (SUVRs) were calculated using cerebellar gray matter region as a reference. Mean cortical Aβ reflected average SUVRs of the orbitofrontal, prefrontal, and superior frontal cortices, lateral temporal, parietal, and occipital lobes, precuneus, and anterior and posterior cingulate ROIs. Aβ PET status (+/−) was defined based on a median of 1.20 mean cortical SUVR.

#### Covariates

Participant education (less than high school/high school; general education diploma or vocational school/at least some college), race (Black/white), and sex (male/female) were recorded at enrollment. Because race and study center are highly confounded, a race-study center variable was used (white-Washington County/white-Forsyth County/Black-Forsyth County/white-Minneapolis/Black-Jackson). *APOE* (coded as 0 *APOE*ε*4* alleles/≥1 *APOEε4* alleles/missing) was genotyped using the TaqMan assay (Applied Biosystems). All other covariates [estimated glomerular filtration rate (eGFR, CDK-EPI) (*74*), hypertension, diabetes, BMI, and smoking status] were assessed at visits concurrent with plasma proteomic measurements used in the current analyses.

#### Statistical analyses

We used Cox proportional hazards regression models to examine the associations of plasma GDF15 abundance with incident dementia risk adjusted for demographic variables (age, sex, race-study center, and education), *APOEε4* status, eGFR, and cardiovascular risk factors (BMI, diabetes, hypertension, and smoking status). The proportional hazards assumption was tested by plotting Kaplan-Meier survival curves and by computing and plotting Schoenfeld residuals (fig. S16). Covariates that did not meet the proportional hazards assumption were incorporated in sensitivity analyses as stratified variables or with a time interaction (covariate × time) to determine whether results differed from that derived in the primary analyses. Multiple linear regression models, adjusted for the covariates listed above, were used to relate GDF15 levels to plasma biomarkers. The same multiple linear regression models were implemented to relate GDF15 levels with cross-sectional neuroimaging measures, which additionally adjusted for intracranial volume (ICV). Analyses were conducted using R v4.4.2 and Stata, version 17.

### UK Biobank

#### Population

We analyzed data from a subset of participants from the UKB study, a population-based cohort of more than half a million individuals aged 37 to 73 years at study entry. Blood samples used for plasma proteomics were collected at participants’ first visit between 2006 and 2010. Participants with missing plasma proteomics or covariate data, those younger than 65 at the date of censoring, and those who developed dementia before study entry were excluded from analyses. The collection and use of UKB data are approved by the Northwest Multi-Center Research Ethics Committee (Research Ethics Committee reference 11/NW/0382). All participants provided informed consent to use their data, health records, and biological materials for research purposes. The present study was conducted under the UKB application number 83534.

#### Plasma proteomics

Plasma proteins were measured using Olink Explore 3072, as described previously (*75*). Data preprocessing followed standard UKB quality control (QC) procedures; normalized protein expression values below the lower limits of detection were preserved, and log_2_-transformed values were used in analyses. The median intra-assay CV for GDF15 was 6.5%.

#### Dementia diagnosis

Dementia diagnoses were ascertained from hospital inpatient records (Hospital Episode Statistics) for all participants, with a subset of participants (45%) additionally having primary care (General Practice) data. Diagnoses were determined from ICD-9 or ICD-10 (International Classification of Diseases, 9th Revision or 10th Revision) codes in the whole cohort as well as Read v2 or Clinical Terms Version 3 for primary care data. Specific diagnostic codes for dementia were identified from the UK NHS National Institute for Health and Care Excellence Quality and Outcomes Framework Business Rules, version 37.0. Read v2 and CTV3 codes were converted to ICD-9 and ICD-10 codes using UKB Resource 592 (clinical coding classification systems and maps). Further details on diagnostic codes and ascertainment are available in our recent publication (*76*). We examined ACD as well as AD, VaD, PDD, and FTD. For those who developed dementia, time to event was the interval in years from study entry to the date of diagnosis. For those who were free of dementia, time to event was the interval in years from study entry to 31 October 2022 for those who did not have a death record, and date of death for those who were deceased.

#### Brain MRI

T1-weighted MPRAGE scans were acquired on a 3-T Siemens Skyra as previously described (*77*). Machine learning–derived neuroimaging measures of age- and AD-related brain atrophy were generated using the same methodology as described in the BLSA (see below).

#### Covariates

Information on sex (male/female), race (self-reported ethnic background as white/non-white), study site, and educational attainment (highest education attained) was collected via participant self-reports. *APOE* genotypes were inferred from variants rs7412 and rs429358 on the Affymetrix Axiom/BiLEVE microarray platform (*78*). All other covariates [eGFR-creatinine (CDK-EPI) (*74*), diabetes, BMI, and high cholesterol] were ascertained concurrently with plasma proteomic measurements used in the current analyses.

#### Statistical analyses

Cox proportional hazards regression models were used to estimate the association between GDF15 and risk of incident dementia diagnosis adjusted for age, sex, education, study site, BMI, eGFR, diabetes, and high cholesterol. Multiple linear regression models adjusting for age, sex, BMI, eGFR, hypertension, diabetes, smoking, *APOE*ε*4* status, and total household income were used to examine associations of GDF15 with brain atrophy measures. Race was not adjusted for in UKB analyses given that 94.6% of UKB participants are white (*57*). R (4.3.1) was used for statistical analyses.

### AGES-Reykjavik study

#### Population

A detailed description of the AGES-Reykjavik study (*79*) has been provided previously. Briefly, the AGES-Reykjavik study is a prospective longitudinal cohort study of older Icelandic adults who were initially enrolled in the Reykjavik study that was established in 1967. From 2002 to 2006, 5764 participants previously enrolled in the Reykjavik study were reexamined for the first wave of the AGES-Reykjavik study. This baseline assessment was completed over three visits within a 4- to 6-week window. Participants underwent a comprehensive assessment that included a clinical examination, questionnaires, a battery of cognitive measures, an MRI scan, and a blood draw. The AGES-Reykjavik study was approved by the Icelandic Nation Bioethics Committee (approval number VSN: 00-063), the Icelandic Data Protection Authority, Iceland, and the IRB for the NIA. Written informed consent was obtained from all participants.

#### Serum proteomics

Blood samples used for the current analyses were collected at baseline visit. Serum was prepared using a standardized protocol and stored at −80°C until analysis. The SomaScan v4.1 platform was used to measure proteins. SOMAmers evaluated in the current study passed QC. Values were log_2_ transformed.

#### Dementia adjudication

Dementia classification at the AGES-Reykjavik baseline and 5-year follow-up visit was conducted using a three-step procedure, as described previously (*79*). All participants were administered the Mini-Mental State Examination and the Digit Symbol Substitution Test. Participants who received a low score on either measure were administered a more comprehensive battery of cognitive measures. Participants who received a low score on the Trails A and B measures or the Rey Auditory Verbal Learning Test received an additional assessment, which included a neurologic examination and a proxy interview. Dementia diagnoses (including AD and VaD) at the AGES visits were adjudicated based on consensus during a conference that included a neurologist, a geriatrician, a neuropsychologist, and a neuroradiologist. *DSM-IV* criteria were used to diagnose dementia (*21*). All AGES-Reykjavik participants were followed up for incident dementia through nursing home reports (Resident Assessment Instrument) and death certificates. The follow-up time was up to 16.9 years (until December 2019). Diagnoses for ACD and AD from nursing home reports were based on intake examinations upon entry or standardized procedures carried out in all Icelandic nursing homes. Diagnosis of AD was established according to National Institute of Neurological and Communicative Diseases and Stroke–Alzheimer’s Disease and Related Disorders Association criteria or according to ICD-10 code F00 criteria. Individuals with incident non-AD diagnosis from nursing home reports who also had any VaD ICD-10 code (F01) in hospital records were considered as incident VAD cases in addition to those diagnosed at the AGES follow-up visit.

#### Covariates

Age, sex, education, and lifestyle variables were assessed using questionnaires at baseline. Education was categorized as primary, secondary, college, or university degree. Smoking was categorized as current, former, or never smoker. *APOE* genotyping was assessed using microplate array diagonal gel electrophoresis. BMI and hypertension were assessed at baseline. BMI was calculated as weight (kg) divided by height squared (m^2^), and hypertension was defined as antihypertensive treatment or blood pressure > 140/90 mmHg. Type 2 diabetes was defined from self-reported diabetes, diabetes medication use, or fasting plasma glucose ≥ 7 mM. Serum creatinine was measured using a Roche Hitachi 912 instrument, and eGFR was derived with the four-variable modification of diet in renal disease study equation (*80*).

#### Statistical analyses

Cox proportional hazards regression models were used to examine the association between the serum GDF15 and incident dementia risk occurring between the baseline visit and December 2019. Models were adjusted for baseline age, sex, education, *APOE*ε*4*, eGFR-creatinine, BMI, diabetes, hypertension, and smoking status. R (4.4.3) was used for statistical analyses.

### Baltimore Longitudinal Study of Aging

#### Population

The BLSA is an ongoing community-based longitudinal study of physiological and psychological aging, which has been described in detail previously (*28*, *81*). Recruitment and enrollment for the BLSA have been previously described (*82*, *83*). In these analyses, we included 3-T MRI scans acquired in 2008 to 2010, at which point blood was drawn for proteomic and AD and related dementia plasma biomarker analyses. The BLSA protocol was approved by the IRB of the National Institute of Environmental Health Science, National Institutes of Health (NIH) (IRB03AG0325), and all participants provided written informed consent before participation. Participants were eligible for inclusion if they had available proteomic and MRI or plasma biomarker data and were excluded based on missing data and baseline cognitive impairment. Participants with cognitive impairment were retained in analyses examining the associations of GDF15 with cognitive status.

#### Plasma proteomics

Proteins in plasma were measured using the SomaScan v4.1 platform, as described previously (*84*). QC was assessed using blind technical replicates, and analytes that failed reproducibility thresholds (CV > 50%) were removed (*N* = 20); GDF15 demonstrated low technical variability (CV = 4.5%). To reduce skewness and limit the influence of extreme values, protein distributions were log_2_ transformed and winsorized at ±5 SD.

#### Other plasma biomarkers

Plasma Aβ_40_, Aβ_42_, GFAP, NfL, and pTau-181 were measured using the Simoa N4PE and pTau-181 (V2) assays. Assay performance was monitored using duplicate samples, demonstrating low analytical variability across biomarkers (CVs ≤ 5%). Duplicate values were averaged. The Aβ_42/40_ ratio was used in analyses, while GFAP, NfL, and pTau-181 were log_2_ transformed; all biomarkers were standardized and winsorized at ±5 SD to minimize the influence of extreme values.

#### Cognitive status adjudication

Cognitive status was determined through a structured consensus process incorporating longitudinal clinical, cognitive, and functional data, following established BLSA protocols (*83*). Participants meeting predefined screening threshold were reviewed at multidisciplinary case conferences, where diagnoses of MCI and dementia were assigned using Petersen criteria and standard diagnostic frameworks for neurodegenerative disease [i.e., the *Diagnostic and Statistical Manual of Mental Disorders* (*Third Edition Revised*) and the National Institute of Neurological and Communicative Disorders and Stroke-Alzheimer’s Disease and Related Disorders Association criteria].

#### Brain MRI

Structural MRI data were obtained using T1-weighted MPRAGE sequences collected on a 3-T Philips Achieva scanner. All images were processed using established BLSA neuroimaging pipelines. Brain regions were delineated and anatomically labeled using the Multi-atlas Region Segmentation Utilizing Ensembles (MUSE) approach, which applies ensemble-based atlas fusion to achieve robust and reproducible parcellation of brain anatomy across longitudinal T1-weighted MRI data (*85*). Complementary voxel-level representations of brain tissue volumes were derived using the Regional Analysis of Volumes Examined in Normalized Space framework (*86*). These maps quantify local volumetric variation across gray matter, white matter, and CSF compartments following spatial normalization. Neuroimaging outcomes examined in the present study included total brain volume, total gray matter volume, hippocampal volume, and total white matter volume. To characterize multivariate patterns of neurodegeneration, machine learning–derived summary measures were computed from regional brain volumes. The SPARE-AD score was calculated using a supervised classification framework trained to differentiate cognitively unimpaired individuals from those with AD based on structural brain features. This metric has been extensively validated and captures distributed neuroanatomical changes associated with AD and related neurodegenerative conditions (*28*, *87*–*92*). A related metric, the SPARE-BA, was also generated to estimate deviations from normative age-related brain structure. SPARE-BA was trained exclusively on scans from cognitively healthy individuals to model age-associated structural variation in the absence of clinical neurodegenerative disease (*93*). A set of continuous neuroanatomical indices (R1 to R5) was derived using a semisupervised deep representation learning framework based on a generative adversarial network architecture (Surreal-GAN). MUSE-derived regional brain volumes were used as model inputs. The learning framework was designed to identify coordinated patterns of structural variation across the brain by contrasting volumetric profiles observed in younger adults (<50 years) with those observed in older adults (≥50 years). This approach yields multiple low-dimensional scores that quantify the extent to which an individual expresses distinct patterns of brain atrophy, while accommodating heterogeneity in both spatial distribution and temporal expression of neurodegenerative change (*28*). These scores have been trained and validated in a diverse cohort across 11 studies (>49,000 participants), where they predicted age-related clinical traits and disease diagnoses (*30*).

#### Amyloid PET imaging

^11^C-Pittsburgh compound-B distribution volume ratios (DVR; 70 min) were collected on a GE Advance or Siemens High Resolution Research Tomograph scanner immediately following an intravenous bolus injection of approximately 555 MBq of the radiotracer. DVRs were computed using cerebellar gray matter as a reference. Mean cortical Aβ reflected the average DVR values across the cingulate, frontal, parietal (including precuneus), lateral temporal, and lateral occipital regions, excluding the pre- and postcentral gyri. Mean cortical DVR values were harmonized between the two scanners by leveraging longitudinal data available on both scanners for 79 participants. Aβ PET status (+/−) was defined based on a Gaussian mixture model threshold of 1.064 mean cortical DVR (*94*).

#### DNA methylation

DNA methylation was assayed using DNA extracted from blood samples collected at visits with concurrent proteomic data. CpG methylation status was determined using the Illumina Infinium HumanMethylation450 BeadChip (Illumina Inc., San Diego, CA), per the manufacturer’s protocol. Background correction, normalization, and QC of data were conducted using the minfi package. DNA methylation PhenoAge (DNAmPhenoAge) was calculated using blood-based clinical phenotypes and chronological age (*95*). For further details, see Kuo *et al.* (*96*).

#### Covariates

Sex (male/female), race (white/non-white), and education level (years) were defined based on participant self-reports. *APOE*ε*4* carrier status (0 *ε4* alleles/≥1 *ε4* alleles/missing) was defined via PCR with restriction isotyping using the Type IIP enzyme HhaI or the Taqman method. eGFR-creatinine was defined at the time of blood sample collection using the CKD-EPI criteria (*97*). Comorbid diseases that represent potential confounders were defined using a comorbidity index calculated as the sum (score range: 0 to 8; converted to a percentage to account for missing data) of eight conditions: obesity, hypertension, diabetes, cancer, ischemic heart disease, chronic heart failure, chronic kidney disease, and chronic obstructive pulmonary disease (*28*, *98*).

#### Statistical analyses

Logistic regression models adjusted for age, sex, race, education, eGFR, *APOEε4* status, and a comorbidity index were used to examine associations of plasma GDF15 with prevalent cognitive status. Linear regression models adjusting for the aforementioned covariates were used to examine associations of GDF15 with brain volumes and plasma biomarkers. Brain volume analyses were additionally adjusted for ICV (defined at age 70). For VBM analyses, an FDR-corrected threshold of *P* < 0.05 was used to define significant clusters (clustering threshold = 50 voxels). SPARE-AD, SPARE-BA, and R-index analyses were adjusted for the aforementioned covariates except for ICV because ICV-residualized values were used in initial calculations. Analyses were performed using R (4.2.3).

### JHNC cohort

#### Plasma and CSF proteomics

CSF and plasma samples from the same participants were used for proteomic analyses using the SomaScan v4.1 assay. Participants referred to the JHNC cohort consented to banking of residual CSF after clinical testing under an IRB-approved protocol (IRB number: CR00050658/NA_00029413). Samples that did not pass SomaScan QC criteria (CSF *N* = 0; plasma *N* = 2) or were identified as outliers by SomaScan or by principal components analyses (CSF *N* = 3; plasma *N* = 1) were excluded. CVs were calculated using QC pooled, matrix-matched sample replicates provided by SomaLogic to monitor overall assay performance (*28*). Aptamers with CVs > 50% were excluded (CSF *N* = 15; plasma *N* = 18). The median CV for GDF15 was 3.0% for CSF and 3.5% for plasma. Values were log_2_ transformed and winsorized at ±5 SDs.

#### Statistical analyses

To relate levels of GDF15 to the full proteome as measured by SomaScan across biofluids, Spearman correlation analyses were implemented. The R package “pspearman” was used to calculate correlations and provide a two-tailed *P* value estimation. Analyses were performed using R (4.3.0). We conducted overrepresentation analyses using the set of the proteins most strongly correlated with plasma GDF15 (absolute value rho = 0.35 for plasma, 0.2 for CSF) using Enrichr, a public, web-based tool that performs gene set enrichment analysis on user input (*99*–*101*). To make a directionality distinction, separate enrichment analyses were ran on up- and down-regulated proteins. The top five most significant pathways from six public databases [Gene Ontology: Biological Process (GO:BP), Gene Ontology: Molecular Function (GO:MF), Kyoto Encyclopedia of Genes and Genomes (KEGG), MSig Database (MSigDB), Reactome, and Wiki Pathways] were recorded (i.e., 30 pathways per direction-separated analysis). A background gene set of all proteins measured by SomaScan v4.0 (concatenated and repeated terms removed) was used for all analyses.

### Exposing differentiated macrophages to rhGDF15

#### Cohort demographics

Apheresis packs were collected from normal donors (*N* = 6) aged 60 to 72 years old. Normal donors are volunteers and have donated apheresis packs through the Cytapheresis of Volunteer Donors protocol (NIA protocol no. 03-AG-N316). Donors provided informed consent for their donations, and the protocol was approved by the IRB of the National Institute of Environmental Health Science, NIH.

#### Isolation of PBMCs

PBMCs were isolated from the cytapheresis packs by density gradient centrifugation using Ficoll-Paque Plus (GE Healthcare). After isolation, PBMCs were cryopreserved in freezing media consisting of 15% dimethyl sulfoxide (Sigma-Aldrich) in fetal bovine serum (FBS, Life Technologies) at a concentration of 50 million PBMCs/ml. Cryopreserved PBMCs were stored in a liquid nitrogen freezer.

#### Cell culture

Cryovials of human PBMCs were taken out of the liquid nitrogen freezer and immersed at a 37°C water bath with stirring until the medium was partially thawed. Cell suspension was then transferred to 9 ml of cold RPMI 1640 media supplemented with 10% FBS and centrifuged at 456*g* for 10 min. Cells were resuspended in warm RPMI media at a concentration of 2 million PBMC/ml and incubated at 37°C for 1 hour in a humified atmosphere. After incubation, cells were centrifuged, counted, and resuspended at a concentration of 50 million PBMCs/ml in Robosep buffer according to the manufacturer’s instructions. Monocytes were enriched from the PBMCs using an immunomagnetic negative selection cell EasySep human monocytes isolation kit (STEMCELL Technologies) using the automated cell separator RoboSep according to the manufacturer’s instructions (STEMCELL Technologies). After enrichment, 1.5 × 10^6^ monocytes were seeded in 2 ml of ImmunoCult-SF Macrophage Medium (STEMCELL Technologies) and differentiated into macrophages using human granulocyte-macrophage colony-stimulating factor (50 ng/ml; R&D Systems) at a 37°C humidifier. Seven days after seeding, cells were washed once with macrophage medium and then treated with rhGDF15 (0.5 μg/ml; R&D Systems) for 24 hours; after the 24-hour treatment, protein lysates were collected for downstream analysis.

#### Protein isolation

Cells were lysed using a denaturing buffer (50 mM Hepes, 2% SDS) supplemented with protease and phosphatase inhibitors (Thermo Fisher Scientific). After boiling and sonication, samples were stored at −80°C. Protein concentration for each cell extract was measured using commercially available 2-D quant kit (Cytiva); protein extraction efficiency and sample quality were confirmed using SyproRuby stained NuPAGE gels (Thermo Fisher Scientific). Lipids and detergents from protein extracts were removed using the methanol/chloroform extraction protocol (*102*), and purified proteins were resuspended in 30 μl of urea buffer [8 M urea, 2 M thiourea, and 150 mM NaCl (Sigma-Aldrich)], reduced by 50 mM dithiothreitol at 36°C for 1 hour, and alkylated with iodoacetamide (100 mM) at 36°C in the dark for 1 hour. Urea buffer with samples was diluted 12 times using 50 mM ammonium bicarbonate buffer. Proteins were then digested for 18 hours at 36°C using a trypsin/LysC mixture (Promega) in 1:50 (w/w) enzyme-to-protein ratio, and the resulting peptides were desalted using C18 cartridge (Restek), dried, and stored at −80°C until further processing.

#### Sample preparation

TMT16plex (Thermo Fisher Scientific) was used to perform semiquantitative proteomics. Briefly, 100 μg of peptides from 12 cultured macrophage lysates was spiked with 200 fM bacterial beta-galactosidase digest (SCIEX) and treated with TMT reagent as recommended by the manufacturer. All labeled TMT samples were merged and fractionated using the standard basic pH fractionation method.

High-pH RPLC fractionation was performed using the Agilent 1260 bio-inert HPLC system [3.9 mm by 5 mm XBridge BEH Shield RP18 XP VanGuard cartridge and 2.1 mm by 250 mm XBridge Peptide BEH C18 column (Waters)]. Mobile-phase composition was as follows: 10 mM ammonium formate (pH 10) as buffer A and 10 mM ammonium formate and 90% ACN (pH 10) as buffer B (*103*). TMT-labeled peptides were resolved using a linear organic gradient (5 to 50% B in 100 min). Initially, 80 fractions were collected every minute during each LC run. Later, five individual fractions were merged into 15 combined fractions (fractions 1, 16, 31, 46, and 61 = combined as fraction one, fractions 2, 17, 32, 47, and 62 = combined fraction two, and so on). Combined fractions were speed vacuum dried, desalted, and stored at −80°C until final liquid chromatography–tandem mass spectrometry analysis.

#### Mass spectrometry

Purified peptide fractions were analyzed using the Vanquish Neo UHPLC System coupled to an Orbitrap Ascend Tribrid mass spectrometer (Thermo Fisher Scientific). Each fraction was loaded using 2% acetonitrile in water with 0.1% formic acid (flow rate, 10 μl/min) for 5 min onto a trap column (Acclaim PepMap 100 HPLC Column, 75 μm ID, 2 cm long, 3 μm C18, Thermo Fisher Scientific) and then separated using EASY-Spray PepMap Neo UHPLC Column (75 μm, 75 cm long, 2 μm C18, Thermo Fisher Scientific) at 250 nl/min flow rate. The linear organic gradient went from 2 to 35% B in 145 min with a total LC method duration of 180 min. Mobile phases A and B consisted of 0.1% formic acid in water and 0.1% formic acid in acetonitrile, respectively. Tandem mass spectra were obtained using the Orbitrap Ascend Tribrid mass spectrometer with a heated capillary temperature of +320°C and spray voltage set to 2.3 kV. Full MS1 spectra were acquired from 375 to 1500 mass/charge ratio (*m*/*z*) at 240,000 resolution and maximum injection time set to auto mode with automatic gain control (AGC) set to 1 × 10^6^. Dd-MS2 spectra were acquired using a 0.7 *m*/*z* isolation window with normal *m*/*z* range and fixed first mass of 110 *m*/*z*. MS/MS spectra were resolved to 45,000 with maximum injection time set to auto and standard AGC target set to 5 × 10^4^. Over 3 s of total cycle time, most abundant ions were selected for fragmentation with 34% normalized high collision energy. A dynamic exclusion time was set to 60 s to discriminate against previously analyzed ions. The raw data generated from each sample fraction were converted to mascot generic format using MSConvert software (ProteoWizard 3.0.20026) and then searched with Mascot 2.4.1 and X!Tandem CYCLONE (2010.12.01.1) using the SwissProt Human sequences from Uniprot (Version Year 2023, 20,300 sequences) database. Quantitative values were expressed as fold change relative to control samples.

#### Bioinformatic analysis

Analysis of the identified proteins was carried out following the standard workflow of the R package DEP, version 1.26.0 (*104*). Only proteins detected in at least two out of three replicates in at least one experimental condition were included for analysis. Data were normalized using the variance stabilization normalization (VSN) method with the function “normalize_vsn”, and missing values were imputed using random draws from a Gaussian distribution centered around a minimal value with the function “MinProb”. The differential protein expression analysis was performed based on linear models and empirical Bayes statistics using limma via function “test_diff” in the DEP package, with adjustment for variability associated with the donor.

### Deriving biologically informed composite scores

Enrichment of differentially expressed proteins in primary macrophages following treatment with rhGDF15 was conducted using Enrichr as described above. The top five most significant pathways from six public databases (GO:BP, GO:MF, KEGG, MSigDB, Reactome, and Wiki) were recorded (i.e., 30 pathways per direction-separated analysis). Of 180 experimentally determined rhGDF15-enriched pathways [from three samples (full, female only, and male only); 60 pathways per sample (30 up, 30 down)], 120 were able to be reproduced using ARIC plasma proteomic data (SomaScan). At least three proteins in a pathway had to have been measured by both mass spectrometry and SomaScan to be considered reproducible; UniProt IDs were used to determine which proteins were measured by each methodology. Using the set of ARIC SomaScan proteins assigned to each reproducible rhGDF15-enriched pathway, we ran a principal components analysis and used the first principal component (PC) of each pathway to create individualized pathway-specific PC scores using each participant’s measured protein levels. Once PC scores were generated, they were then used as predictor variables in Cox proportional hazards regression models to examine their associations with incident dementia risk over 7- and 20-year follow-up periods (adjusted for all ARIC covariates discussed previously).

### Two-sample MR

To assess the potential causal effects of GDF15 on ADRD and associated endophenotypes, we used the recently developed MR-SPI approach. MR-SPI is a two-sample MR method that first selects valid genetic instruments that satisfy core instrumental variable (IV) assumptions (IV relevance, IV independence, and exclusion restriction assumptions), and then performs postselection inference for reliable causal findings. Specifically, under Leo Tolstoy’s Anna Karenina principle, valid genetic instruments will form the largest group and provide similar ratio estimates. Applying the voting procedure, MR-SPI selects the largest group where genetic instruments mutually vote for each other to be valid. This voting procedure is particularly advantageous in pQTL studies with limited candidate genetic instruments, as it does not rely on any additional distributional assumptions on the genetic effects. Subsequently, MR-SPI uses a searching-and-sampling method to construct a robust CI to mitigate the potential finite-sample IV selection error. By integrating selection of valid genetic instruments with the construction of robust CI, MR-SPI strengthens the reliability of causal inference in MR analysis. The MR-SPI package is available at https://zenodo.org/records/14036275. Here, we performed linkage disequilibrium (LD) clumping with a 10,000-kb window and *R*^2^ = 0.1 using PLINK software (*105*). After removing highly correlated pQTLs for primary (ARIC) and secondary (deCODE) MR analyses, 10 and 37 cis*-*pQTLs were retained as candidate genetic instruments, respectively. By leveraging the LD matrix extracted from a reference panel for potentially weakly correlated pQTLs, joint association estimates were computed using GWAS summary statistics (*106*). Incorporating these joint association estimates into MR-SPI allows for the inclusion of additional pQTLs, thereby enhancing statistical power to select valid IVs and detect causal effects.

### Immuno-phenome-wide association study

To assess the potential causal effects of GDF15 on immune traits, we performed an IPheWAS. IPheWAS was conducted using the FUSION pipeline based on the previously defined cis-pQTL predictions models from individuals of European ancestry in the ARIC study (*34*) against immuno-related GWAS summary statistics datasets from the UKB (*107*, *108*) covering a total of 133 immune traits. To control for multiple testing, a Bonferroni correction was applied based on the number of aptamers modeled across the number of UKB traits (*P* < 3.76 × 10^−4^ = 0.05/133 immune traits).

## References

[1] E. C. B. Johnson, S. Bian, R. U. Haque, E. K. Carter, C. M. Watson, B. A. Gordon, L. Ping, D. M. Duong, M. P. Epstein, E. McDade, N. R. Barthelemy, C. M. Karch, C. Xiong, C. Cruchaga, R. J. Perrin, A. P. Wingo, T. S. Wingo, J. P. Chhatwal, G. S. Day, J. M. Noble, S. B. Berman, R. Martins, N. R. Graff-Radford, P. R. Schofield, T. Ikeuchi, H. Mori, J. Levin, M. Farlow, J. J. Lah, C. Haass, M. Jucker, J. C. Morris, T. L. S. Benzinger, B. R. Roberts, R. J. Bateman, A. M. Fagan, N. T. Seyfried, A. I. Levey, The dominantly inherited Alzheimer, cerebrospinal fluid proteomics define the natural history of autosomal dominant Alzheimer’s disease. Nat. Med. 29, 1979–1988 (2023). 37550416 10.1038/s41591-023-02476-4 PMC10427428

[2] K. A. Walker, J. Chen, L. Shi, Y. Yang, M. Fornage, L. Zhou, P. Schlosser, A. Surapaneni, M. E. Grams, M. R. Duggan, Z. Peng, G. T. Gomez, A. Tin, R. C. Hoogeveen, K. J. Sullivan, P. Ganz, J. V. Lindbohm, M. Kivimaki, A. J. Nevado-Holgado, N. Buckley, R. F. Gottesman, T. H. Mosley, E. Boerwinkle, C. M. Ballantyne, J. Coresh, Proteomics analysis of plasma from middle-aged adults identifies protein markers of dementia risk in later life. Sci. Transl. Med. 15, eadf5681 (2023). 37467317 10.1126/scitranslmed.adf5681 PMC10665113

[3] M. R. Bootcov, A. R. Bauskin, S. M. Valenzuela, A. G. Moore, M. Bansal, X. Y. He, H. P. Zhang, M. Donnellan, S. Mahler, K. Pryor, B. J. Walsh, R. C. Nicholson, W. D. Fairlie, S. B. Por, J. M. Robbins, S. N. Breit, MIC-1, a novel macrophage inhibitory cytokine, is a divergent member of the TGF-beta superfamily. Proc. Natl. Acad. Sci. U.S.A. 94, 11514–11519 (1997). 9326641 10.1073/pnas.94.21.11514 PMC23523

[4] A. Assadi, A. Zahabi, R. A. Hart, GDF15, an update of the physiological and pathological roles it plays: A review. Pflugers Arch. 472, 1535–1546 (2020). 32936319 10.1007/s00424-020-02459-1

[5] M. Conte, C. Giuliani, A. Chiariello, V. Iannuzzi, C. Franceschi, S. Salvioli, GDF15, an emerging key player in human aging. Ageing Res. Rev. 75, 101569 (2022). 35051643 10.1016/j.arr.2022.101569

[6] H. H. Luan, A. Wang, B. K. Hilliard, F. Carvalho, C. E. Rosen, A. M. Ahasic, E. L. Herzog, I. Kang, M. A. Pisani, S. Yu, C. Zhang, A. M. Ring, L. H. Young, R. Medzhitov, GDF15 is an inflammation-induced central mediator of tissue tolerance. Cell 178, 1231–1244.e11 (2019). 31402172 10.1016/j.cell.2019.07.033 PMC6863354

[7] J. Wischhusen, I. Melero, W. H. Fridman, Growth/differentiation factor-15 (GDF-15): From biomarker to novel targetable immune checkpoint. Front. Immunol. 11, 951 (2020). 32508832 10.3389/fimmu.2020.00951 PMC7248355

[8] V. W. W. Tsai, Y. Husaini, A. Sainsbury, D. A. Brown, S. N. Breit, The MIC-1/GDF15-GFRAL pathway in energy homeostasis: Implications for obesity, cachexia, and other associated diseases. Cell Metab. 28, 353–368 (2018). 30184485 10.1016/j.cmet.2018.07.018

[9] L. Rochette, A. Meloux, M. Zeller, Y. Cottin, C. Vergely, Functional roles of GDF15 in modulating microenvironment to promote carcinogenesis. Biochim. Biophys. Acta Mol. Basis Dis. 1866, 165798 (2020). 32304740 10.1016/j.bbadis.2020.165798

[10] S. Lemmela, E. M. Wigmore, C. Benner, A. S. Havulinna, R. M. Y. Ong, T. Kempf, K. C. Wollert, S. Blankenberg, T. Zeller, J. E. Peters, V. Salomaa, M. Fritsch, R. March, A. Palotie, M. Daly, A. S. Butterworth, M. Kinnunen, D. S. Paul, A. Matakidou, Integrated analyses of growth differentiation factor-15 concentration and cardiometabolic diseases in humans. eLife 11, e76272 (2022). 35916366 10.7554/eLife.76272 PMC9391041

[11] V. Nair, C. Robinson-Cohen, M. R. Smith, K. A. Bellovich, Z. Y. Bhat, M. Bobadilla, F. Brosius, I. H. de Boer, L. Essioux, I. Formentini, C. A. Gadegbeku, D. Gipson, J. Hawkins, J. Himmelfarb, B. Kestenbaum, M. Kretzler, M. C. Magnone, K. Perumal, S. Steigerwalt, W. Ju, N. Bansal, Growth differentiation factor-15 and risk of CKD progression. J. Am. Soc. Nephrol. 28, 2233–2240 (2017). 28159780 10.1681/ASN.2016080919 PMC5491285

[12] M. Conte, J. Sabbatinelli, A. Chiariello, M. Martucci, A. Santoro, D. Monti, M. Arcaro, D. Galimberti, E. Scarpini, A. R. Bonfigli, A. Giuliani, F. Olivieri, C. Franceschi, S. Salvioli, Disease-specific plasma levels of mitokines FGF21, GDF15, and humanin in type II diabetes and Alzheimer's disease in comparison with healthy aging. Geroscience 43, 985–1001 (2021). 33131010 10.1007/s11357-020-00287-w PMC8110619

[13] T. Fuchs, J. N. Trollor, J. Crawford, D. A. Brown, B. T. Baune, K. Samaras, L. Campbell, S. N. Breit, H. Brodaty, P. Sachdev, E. Smith, Macrophage inhibitory cytokine-1 is associated with cognitive impairment and predicts cognitive decline—The Sydney Memory and Aging Study. Aging Cell 12, 882–889 (2013). 23758647 10.1111/acel.12116

[14] E. R. McGrath, J. J. Himali, D. Levy, S. C. Conner, C. DeCarli, M. P. Pase, T. Ninomiya, T. Ohara, P. Courchesne, C. L. Satizabal, R. S. Vasan, A. S. Beiser, S. Seshadri, Growth differentiation factor 15 and NT-proBNP as blood-based markers of vascular brain injury and dementia. J. Am. Heart Assoc. 9, e014659 (2020). 32921207 10.1161/JAHA.119.014659 PMC7792414

[15] The GTEx Consortium, The Genotype-Tissue Expression (GTEx) project. Nat. Genet. 45, 580–585 (2013). 23715323 10.1038/ng.2653 PMC4010069

[16] M. Uhlen, L. Fagerberg, B. M. Hallstrom, C. Lindskog, P. Oksvold, A. Mardinoglu, A. Sivertsson, C. Kampf, E. Sjostedt, A. Asplund, I. Olsson, K. Edlund, E. Lundberg, S. Navani, C. A. Szigyarto, J. Odeberg, D. Djureinovic, J. O. Takanen, S. Hober, T. Alm, P. H. Edqvist, H. Berling, H. Tegel, J. Mulder, J. Rockberg, P. Nilsson, J. M. Schwenk, M. Hamsten, K. von Feilitzen, M. Forsberg, L. Persson, F. Johansson, M. Zwahlen, G. von Heijne, J. Nielsen, F. Ponten, Proteomics. Tissue-based map of the human proteome. Science 347, 1260419 (2015). 25613900 10.1126/science.1260419

[17] A. Chiariello, S. Valente, G. Pasquinelli, A. Baracca, G. Sgarbi, G. Solaini, V. Medici, V. Fantini, T. E. Poloni, M. Tognocchi, M. Arcaro, D. Galimberti, C. Franceschi, M. Capri, S. Salvioli, M. Conte, The expression pattern of GDF15 in human brain changes during aging and in Alzheimer’s disease. Front. Aging Neurosci. 14, 1058665 (2022). 36698863 10.3389/fnagi.2022.1058665 PMC9869280

[18] G. M. McKhann, D. S. Knopman, H. Chertkow, B. T. Hyman, C. R. Jack Jr., C. H. Kawas, W. E. Klunk, W. J. Koroshetz, J. J. Manly, R. Mayeux, R. C. Mohs, J. C. Morris, M. N. Rossor, P. Scheltens, M. C. Carrillo, B. Thies, S. Weintraub, C. H. Phelps, The diagnosis of dementia due to Alzheimer’s disease: Recommendations from the National Institute on Aging-Alzheimer’s Association workgroups on diagnostic guidelines for Alzheimer’s disease. Alzheimers Dement. 7, 263–269 (2011). 21514250 10.1016/j.jalz.2011.03.005 PMC3312024

[19] American Psychiatric Association, *Diagnostic and Statistical Manual of Mental Disorders* (American Psychiatric Association, ed. 5, 2013).

[20] D. S. Knopman, R. F. Gottesman, A. R. Sharrett, L. M. Wruck, B. G. Windham, L. Coker, A. L. Schneider, S. Hengrui, A. Alonso, J. Coresh, M. S. Albert, T. H. Mosley Jr., Mild cognitive impairment and dementia prevalence: The Atherosclerosis Risk in Communities Neurocognitive Study (ARIC-NCS). Alzheimers Dement. 2, 1–11 (2016). 10.1016/j.dadm.2015.12.002 PMC4772876 26949733

[21] APA, *The Curated Reference Collection in Neuroscience and Biobehavioral Psychology* (American Psychiatric Association, 1994).

[22] D. A. Gadd, R. F. Hillary, D. L. McCartney, L. Shi, A. Stolicyn, N. A. Robertson, R. M. Walker, R. I. McGeachan, A. Campbell, S. Xueyi, M. C. Barbu, C. Green, S. W. Morris, M. A. Harris, E. V. Backhouse, J. M. Wardlaw, J. D. Steele, D. A. Oyarzun, G. Muniz-Terrera, C. Ritchie, A. Nevado-Holgado, T. Chandra, C. Hayward, K. L. Evans, D. J. Porteous, S. R. Cox, H. C. Whalley, A. M. McIntosh, R. E. Marioni, Integrated methylome and phenome study of the circulating proteome reveals markers pertinent to brain health. Nat. Commun. 13, 4670 (2022). 35945220 10.1038/s41467-022-32319-8 PMC9363452

[23] M. Yao, G. W. Miller, B. N. Vardarajan, A. A. Baccarelli, Z. Guo, Z. Liu, Deciphering proteins in Alzheimer’s disease: A new Mendelian randomization method integrated with AlphaFold3 for 3D structure prediction. Cell Genom. 4, 100700 (2024). 39637861 10.1016/j.xgen.2024.100700 PMC11701259

[24] I. E. Jansen, S. J. van der Lee, D. Gomez-Fonseca, I. de Rojas, M. C. Dalmasso, B. Grenier-Boley, A. Zettergren, A. Mishra, M. Ali, V. Andrade, C. Bellenguez, L. Kleineidam, F. Küçükali, Y. J. Sung, N. Tesí, E. M. Vromen, D. P. Wightman, D. Alcolea, M. Alegret, I. Alvarez, P. Amouyel, L. Athanasiu, S. Bahrami, H. Bailly, O. Belbin, S. Bergh, L. Bertram, G. J. Biessels, K. Blennow, R. Blesa, M. Boada, A. Boland, K. Buerger, Á. Carracedo, L. Cervera-Carles, G. Chene, J. A. H. R. Claassen, S. Debette, J.-F. Deleuze, P. P. de Deyn, J. Diehl-Schmid, S. Djurovic, O. Dols-Icardo, C. Dufouil, E. Duron, E. Düzel, T. Fladby, J. Fortea, L. Frölich, P. García-González, M. Garcia-Martinez, I. Giegling, O. Goldhardt, J. Gobom, T. Grimmer, A. Haapasalo, H. Hampel, O. Hanon, L. Hausner, S. Heilmann-Heimbach, S. Helisalmi, M. T. Heneka, I. Hernández, S.-K. Herukka, H. Holstege, J. Jarholm, S. Kern, A.-B. Knapskog, A. M. Koivisto, J. Kornhuber, T. Kuulasmaa, C. Lage, C. Laske, V. Leinonen, P. Lewczuk, A. Lleó, A. L. de Munain, S. Lopez-Garcia, W. Maier, M. Marquié, M. O. Mol, L. Montrreal, F. Moreno, S. Moreno-Grau, G. Nicolas, M. M. Nöthen, A. Orellana, L. Pålhaugen, J. M. Papma, F. Pasquier, R. Perneczky, O. Peters, Y. A. L. Pijnenburg, J. Popp, D. Posthuma, A. Pozueta, J. Priller, R. Puerta, I. Quintela, I. Ramakers, E. Rodriguez-Rodriguez, D. Rujescu, I. Saltvedt, P. Sanchez-Juan, P. Scheltens, N. Scherbaum, M. Schmid, A. Schneider, G. Selbæk, P. Selnes, A. Shadrin, I. Skoog, H. Soininen, L. Tárraga, S. Teipel, B. Tijms, M. Tsolaki, C. Van Broeckhoven, J. Van Dongen, J. C. van Swieten, R. Vandenberghe, J.-S. Vidal, P. J. Visser, J. Vogelgsang, M. Waern, M. Wagner, J. Wiltfang, M. M. J. Wittens, H. Zetterberg, M. Zulaica, C. M. van Duijn, M. Bjerke, S. Engelborghs, F. Jessen, C. E. Teunissen, P. Pastor, M. Hiltunen, M. Ingelsson, O. A. Andreassen, J. Clarimón, K. Sleegers, A. Ruiz, A. Ramirez, C. Cruchaga, J.-C. Lambert, W. van der Flier, Genome-wide meta-analysis for Alzheimer’s disease cerebrospinal fluid biomarkers. Acta Neuropathol. 144, 821–842 (2022). 36066633 10.1007/s00401-022-02454-z PMC9547780

[25] C. Bellenguez, F. Kucukali, I. E. Jansen, L. Kleineidam, S. Moreno-Grau, N. Amin, A. C. Naj, R. Campos-Martin, B. Grenier-Boley, V. Andrade, P. A. Holmans, A. Boland, V. Damotte, S. J. van der Lee, M. R. Costa, T. Kuulasmaa, Q. Yang, I. de Rojas, J. C. Bis, A. Yaqub, I. Prokic, J. Chapuis, S. Ahmad, V. Giedraitis, D. Aarsland, P. Garcia-Gonzalez, C. Abdelnour, E. Alarcon-Martin, D. Alcolea, M. Alegret, I. Alvarez, V. Alvarez, N. J. Armstrong, A. Tsolaki, C. Antunez, I. Appollonio, M. Arcaro, S. Archetti, A. A. Pastor, B. Arosio, L. Athanasiu, H. Bailly, N. Banaj, M. Baquero, S. Barral, A. Beiser, A. B. Pastor, J. E. Below, P. Benchek, L. Benussi, C. Berr, C. Besse, V. Bessi, G. Binetti, A. Bizarro, R. Blesa, M. Boada, E. Boerwinkle, B. Borroni, S. Boschi, P. Bossu, G. Brathen, J. Bressler, C. Bresner, H. Brodaty, K. J. Brookes, L. I. Brusco, D. Buiza-Rueda, K. Burger, V. Burholt, W. S. Bush, M. Calero, L. B. Cantwell, G. Chene, J. Chung, M. L. Cuccaro, A. Carracedo, R. Cecchetti, L. Cervera-Carles, C. Charbonnier, H. H. Chen, C. Chillotti, S. Ciccone, J. Claassen, C. Clark, E. Conti, A. Corma-Gomez, E. Costantini, C. Custodero, D. Daian, M. C. Dalmasso, A. Daniele, E. Dardiotis, J. F. Dartigues, P. P. de Deyn, K. de Paiva Lopes, L. D. de Witte, S. Debette, J. Deckert, T. Del Ser, N. Denning, A. DeStefano, M. Dichgans, J. Diehl-Schmid, M. Diez-Fairen, P. D. Rossi, S. Djurovic, E. Duron, E. Duzel, C. Dufouil, G. Eiriksdottir, S. Engelborghs, V. Escott-Price, A. Espinosa, M. Ewers, K. M. Faber, T. Fabrizio, S. F. Nielsen, D. W. Fardo, L. Farotti, C. Fenoglio, M. Fernandez-Fuertes, R. Ferrari, C. B. Ferreira, E. Ferri, B. Fin, P. Fischer, T. Fladby, K. Fliessbach, B. Fongang, M. Fornage, J. Fortea, T. M. Foroud, S. Fostinelli, N. C. Fox, E. Franco-Macias, M. J. Bullido, A. Frank-Garcia, L. Froelich, B. Fulton-Howard, D. Galimberti, J. M. Garcia-Alberca, P. Garcia-Gonzalez, S. Garcia-Madrona, G. Garcia-Ribas, R. Ghidoni, I. Giegling, G. Giorgio, A. M. Goate, O. Goldhardt, D. Gomez-Fonseca, A. Gonzalez-Perez, C. Graff, G. Grande, E. Green, T. Grimmer, E. Grunblatt, M. Grunin, V. Gudnason, T. Guetta-Baranes, A. Haapasalo, G. Hadjigeorgiou, J. L. Haines, K. L. Hamilton-Nelson, H. Hampel, O. Hanon, J. Hardy, A. M. Hartmann, L. Hausner, J. Harwood, S. Heilmann-Heimbach, S. Helisalmi, M. T. Heneka, I. Hernandez, M. J. Herrmann, P. Hoffmann, C. Holmes, H. Holstege, R. H. Vilas, M. Hulsman, J. Humphrey, G. J. Biessels, X. Jian, C. Johansson, G. R. Jun, Y. Kastumata, J. Kauwe, P. G. Kehoe, L. Kilander, A. K. Stahlbom, M. Kivipelto, A. Koivisto, J. Kornhuber, M. H. Kosmidis, W. A. Kukull, P. P. Kuksa, B. W. Kunkle, A. B. Kuzma, C. Lage, E. J. Laukka, L. Launer, A. Lauria, C. Y. Lee, J. Lehtisalo, O. Lerch, A. Lleo, W. Longstreth Jr., O. Lopez, A. L. de Munain, S. Love, M. Lowemark, L. Luckcuck, K. L. Lunetta, Y. Ma, J. Macias, C. A. MacLeod, W. Maier, F. Mangialasche, M. Spallazzi, M. Marquie, R. Marshall, E. R. Martin, A. M. Montes, C. M. Rodriguez, C. Masullo, R. Mayeux, S. Mead, P. Mecocci, M. Medina, A. Meggy, S. Mehrabian, S. Mendoza, M. Menendez-Gonzalez, P. Mir, S. Moebus, M. Mol, L. Molina-Porcel, L. Montrreal, L. Morelli, F. Moreno, K. Morgan, T. Mosley, M. M. Nothen, C. Muchnik, S. Mukherjee, B. Nacmias, T. Ngandu, G. Nicolas, B. G. Nordestgaard, R. Olaso, A. Orellana, M. Orsini, G. Ortega, A. Padovani, C. Paolo, G. Papenberg, L. Parnetti, F. Pasquier, P. Pastor, G. Peloso, A. Perez-Cordon, J. Perez-Tur, P. Pericard, O. Peters, Y. A. L. Pijnenburg, J. A. Pineda, G. Pinol-Ripoll, C. Pisanu, T. Polak, J. Popp, D. Posthuma, J. Priller, R. Puerta, O. Quenez, I. Quintela, J. Q. Thomassen, A. Rabano, I. Rainero, F. Rajabli, I. Ramakers, L. M. Real, M. J. T. Reinders, C. Reitz, D. Reyes-Dumeyer, P. Ridge, S. Riedel-Heller, P. Riederer, N. Roberto, E. Rodriguez-Rodriguez, A. Rongve, I. R. Allende, M. Rosende-Roca, J. L. Royo, E. Rubino, D. Rujescu, M. E. Saez, P. Sakka, I. Saltvedt, A. Sanabria, M. B. Sanchez-Arjona, F. Sanchez-Garcia, P. S. Juan, R. Sanchez-Valle, S. B. Sando, C. Sarnowski, C. L. Satizabal, M. Scamosci, N. Scarmeas, E. Scarpini, P. Scheltens, N. Scherbaum, M. Scherer, M. Schmid, A. Schneider, J. M. Schott, G. Selbaek, D. Seripa, M. Serrano, J. Sha, A. A. Shadrin, O. Skrobot, S. Slifer, G. J. L. Snijders, H. Soininen, V. Solfrizzi, A. Solomon, Y. Song, S. Sorbi, O. Sotolongo-Grau, G. Spalletta, A. Spottke, A. Squassina, E. Stordal, J. P. Tartan, L. Tarraga, N. Tesi, A. Thalamuthu, T. Thomas, G. Tosto, L. Traykov, L. Tremolizzo, A. Tybjaerg-Hansen, A. Uitterlinden, A. Ullgren, I. Ulstein, S. Valero, O. Valladares, C. V. Broeckhoven, J. Vance, B. N. Vardarajan, A. van der Lugt, J. V. Dongen, J. van Rooij, J. van Swieten, R. Vandenberghe, F. Verhey, J. S. Vidal, J. Vogelgsang, M. Vyhnalek, M. Wagner, D. Wallon, L. S. Wang, R. Wang, L. Weinhold, J. Wiltfang, G. Windle, B. Woods, M. Yannakoulia, H. Zare, Y. Zhao, X. Zhang, C. Zhu, M. Zulaica, Eadb, Gr@Ace, Degesco, Eadi, Gerad, Demgene, FinnGen, Adgc, Charge, L. A. Farrer, B. M. Psaty, M. Ghanbari, T. Raj, P. Sachdev, K. Mather, F. Jessen, M. A. Ikram, A. de Mendonca, J. Hort, M. Tsolaki, M. A. Pericak-Vance, P. Amouyel, J. Williams, R. Frikke-Schmidt, J. Clarimon, J. F. Deleuze, G. Rossi, S. Seshadri, O. A. Andreassen, M. Ingelsson, M. Hiltunen, K. Sleegers, G. D. Schellenberg, C. M. van Duijn, R. Sims, W. M. van der Flier, A. Ruiz, A. Ramirez, J. C. Lambert, New insights into the genetic etiology of Alzheimer’s disease and related dementias. Nat. Genet. 54, 412–436 (2022). 35379992 10.1038/s41588-022-01024-z PMC9005347

[26] M. I. Kurki, J. Karjalainen, P. Palta, T. P. Sipila, K. Kristiansson, K. M. Donner, M. P. Reeve, H. Laivuori, M. Aavikko, M. A. Kaunisto, A. Loukola, E. Lahtela, H. Mattsson, P. Laiho, P. D. B. Parolo, A. A. Lehisto, M. Kanai, N. Mars, J. Ramo, T. Kiiskinen, H. O. Heyne, K. Veerapen, S. Rueger, S. Lemmela, W. Zhou, S. Ruotsalainen, K. Parn, T. Hiekkalinna, S. Koskelainen, T. Paajanen, V. Llorens, J. Gracia-Tabuenca, H. Siirtola, K. Reis, A. G. Elnahas, B. Sun, C. N. Foley, K. Aalto-Setala, K. Alasoo, M. Arvas, K. Auro, S. Biswas, A. Bizaki-Vallaskangas, O. Carpen, C. Y. Chen, O. A. Dada, Z. Ding, M. G. Ehm, K. Eklund, M. Farkkila, H. Finucane, A. Ganna, A. Ghazal, R. R. Graham, E. M. Green, A. Hakanen, M. Hautalahti, A. K. Hedman, M. Hiltunen, R. Hinttala, I. Hovatta, X. Hu, A. Huertas-Vazquez, L. Huilaja, J. Hunkapiller, H. Jacob, J. N. Jensen, H. Joensuu, S. John, V. Julkunen, M. Jung, J. Junttila, K. Kaarniranta, M. Kahonen, R. Kajanne, L. Kallio, R. Kalviainen, J. Kaprio, N. K. FinnGen, J. Kettunen, E. Kilpelainen, T. Kilpi, K. Klinger, V. M. Kosma, T. Kuopio, V. Kurra, T. Laisk, J. Laukkanen, N. Lawless, A. Liu, S. Longerich, R. Magi, J. Makela, A. Makitie, A. Malarstig, A. Mannermaa, J. Maranville, A. Matakidou, T. Meretoja, S. V. Mozaffari, M. E. K. Niemi, M. Niemi, T. Niiranen, O. D. CJ, M. E. Obeidat, G. Okafo, H. M. Ollila, A. Palomaki, T. Palotie, J. Partanen, D. S. Paul, M. Pelkonen, R. K. Pendergrass, S. Petrovski, A. Pitkaranta, A. Platt, D. Pulford, E. Punkka, P. Pussinen, N. Raghavan, F. Rahimov, D. Rajpal, N. A. Renaud, B. Riley-Gillis, R. Rodosthenous, E. Saarentaus, A. Salminen, E. Salminen, V. Salomaa, J. Schleutker, R. Serpi, H. Y. Shen, R. Siegel, K. Silander, S. Siltanen, S. Soini, H. Soininen, J. H. Sul, I. Tachmazidou, K. Tasanen, P. Tienari, S. Toppila-Salmi, T. Tukiainen, T. Tuomi, J. A. Turunen, J. C. Ulirsch, F. Vaura, P. Virolainen, J. Waring, D. Waterworth, R. Yang, M. Nelis, A. Reigo, A. Metspalu, L. Milani, T. Esko, C. Fox, A. S. Havulinna, M. Perola, S. Ripatti, A. Jalanko, T. Laitinen, T. P. Makela, R. Plenge, M. McCarthy, H. Runz, M. J. Daly, A. Palotie, FinnGen provides genetic insights from a well-phenotyped isolated population. Nature 613, 508–518 (2023). 36653562 10.1038/s41586-022-05473-8 PMC9849126

[27] E. Ferkingstad, P. Sulem, B. A. Atlason, G. Sveinbjornsson, M. I. Magnusson, E. L. Styrmisdottir, K. Gunnarsdottir, A. Helgason, A. Oddsson, B. V. Halldorsson, B. O. Jensson, F. Zink, G. H. Halldorsson, G. Masson, G. A. Arnadottir, H. Katrinardottir, K. Juliusson, M. K. Magnusson, O. T. Magnusson, R. Fridriksdottir, S. Saevarsdottir, S. A. Gudjonsson, S. N. Stacey, S. Rognvaldsson, T. Eiriksdottir, T. A. Olafsdottir, V. Steinthorsdottir, V. Tragante, M. O. Ulfarsson, H. Stefansson, I. Jonsdottir, H. Holm, T. Rafnar, P. Melsted, J. Saemundsdottir, G. L. Norddahl, S. H. Lund, D. F. Gudbjartsson, U. Thorsteinsdottir, K. Stefansson, Large-scale integration of the plasma proteome with genetics and disease. Nat. Genet. 53, 1712–1721 (2021). 34857953 10.1038/s41588-021-00978-w

[28] M. R. Duggan, Z. Yang, Y. Cui, H. E. Dark, J. Wen, G. Erus, T. J. Hohman, J. Chen, A. Lewis, A. Moghekar, J. Coresh, S. M. Resnick, C. Davatzikos, K. A. Walker, Proteomic analyses reveal plasma EFEMP1 and CXCL12 as biomarkers and determinants of neurodegeneration. Alzheimers Dement. 20, 6486–6505 (2024). 39129354 10.1002/alz.14142 PMC11497673

[29] Z. Yang, J. Wen, G. Erus, S. T. Govindarajan, R. Melhem, E. Mamourian, Y. Cui, D. Srinivasan, A. Abdulkadir, P. Parmpi, K. Wittfeld, H. J. Grabe, R. Bulow, S. Frenzel, D. Tosun, M. Bilgel, Y. An, D. Yi, D. S. Marcus, P. LaMontagne, T. L. S. Benzinger, S. R. Heckbert, T. R. Austin, S. R. Waldstein, M. K. Evans, A. B. Zonderman, L. J. Launer, A. Sotiras, M. A. Espeland, C. L. Masters, P. Maruff, J. Fripp, A. Toga, S. O’Bryant, M. M. Chakravarty, S. Villeneuve, S. C. Johnson, J. C. Morris, M. S. Albert, K. Yaffe, H. Volzke, L. Ferrucci, N. R. Bryan, R. T. Shinohara, Y. Fan, M. Habes, P. A. Lalousis, N. Koutsouleris, D. A. Wolk, S. M. Resnick, H. Shou, I. M. Nasrallah, C. Davatzikos, Five dominant dimensions of brain aging are identified via deep learning: Associations with clinical, lifestyle, and genetic measures. medRxiv 23300642 [Preprint] (2023). 10.1101/2023.12.29.23300642.

[30] Z. Yang, J. Wen, G. Erus, S. T. Govindarajan, R. Melhem, E. Mamourian, Y. Cui, D. Srinivasan, A. Abdulkadir, P. Parmpi, K. Wittfeld, H. J. Grabe, R. Bulow, S. Frenzel, D. Tosun, M. Bilgel, Y. An, D. Yi, D. S. Marcus, P. LaMontagne, T. L. S. Benzinger, S. R. Heckbert, T. R. Austin, S. R. Waldstein, M. K. Evans, A. B. Zonderman, L. J. Launer, A. Sotiras, M. A. Espeland, C. L. Masters, P. Maruff, J. Fripp, A. W. Toga, S. O’Bryant, M. M. Chakravarty, S. Villeneuve, S. C. Johnson, J. C. Morris, M. S. Albert, K. Yaffe, H. Volzke, L. Ferrucci, R. Nick Bryan, R. T. Shinohara, Y. Fan, M. Habes, P. A. Lalousis, N. Koutsouleris, D. A. Wolk, S. M. Resnick, H. Shou, I. M. Nasrallah, C. Davatzikos, Brain aging patterns in a large and diverse cohort of 49,482 individuals. Nat. Med. 30, 3015–3026 (2024). 39147830 10.1038/s41591-024-03144-x PMC11483219

[31] H. E. Dark, M. R. Duggan, K. A. Walker, Plasma biomarkers for Alzheimer’s and related dementias: A review and outlook for clinical neuropsychology. Arch. Clin. Neuropsychol. 39, 313–324 (2024). 38520383 10.1093/arclin/acae019 PMC11484593

[32] R. Mancuso, N. Fattorelli, A. Martinez-Muriana, E. Davis, L. Wolfs, J. Van Den Daele, I. Geric, J. Premereur, P. Polanco, B. Bijnens, P. Preman, L. Serneels, S. Poovathingal, S. Balusu, C. Verfaillie, M. Fiers, B. De Strooper, Xenografted human microglia display diverse transcriptomic states in response to Alzheimer’s disease-related amyloid-beta pathology. Nat. Neurosci. 27, 886–900 (2024). 38539015 10.1038/s41593-024-01600-y PMC11089003

[33] I. Hristovska, A. P. Binette, A. Kumar, C. Gaiteri, L. Karlsson, O. Strandberg, S. Janelidze, D. van Westen, E. Stomrud, S. Palmqvist, R. Ossenkoppele, N. Mattsson-Carlgren, J. W. Vogel, O. Hansson, Identification of distinct and shared biomarker panels in different manifestations of cerebral small vessel disease through proteomic profiling. Nat. Aging 6, 703–721 (2024). 10.1038/s43587-026-01081-7 PMC13004690 41735646

[34] J. Zhang, D. Dutta, A. Kottgen, A. Tin, P. Schlosser, M. E. Grams, B. Harvey, CKDGen Consortium, B. Yu, E. Boerwinkle, J. Coresh, N. Chatterjee, Plasma proteome analyses in individuals of European and African ancestry identify cis-pQTLs and models for proteome-wide association studies. Nat. Genet. 54, 593–602 (2022). 35501419 10.1038/s41588-022-01051-w PMC9236177

[35] H. Zhu, X. Zhou, Transcriptome-wide association studies: A view from Mendelian randomization. Quant. Biol. 9, 107–121 (2021). 35433074 10.1007/s40484-020-0207-4 PMC9009829

[36] O. Zenarruzabeitia, J. Vitalle, C. Eguizabal, V. R. Simhadri, F. Borrego, The biology and disease relevance of CD300a, an inhibitory receptor for phosphatidylserine and phosphatidylethanolamine. J. Immunol. 194, 5053–5060 (2015). 25980030 10.4049/jimmunol.1500304

[37] D. K. Kaushik, A. Bhattacharya, R. Mirzaei, K. S. Rawji, Y. Ahn, J. M. Rho, V. W. Yong, Enhanced glycolytic metabolism supports transmigration of brain-infiltrating macrophages in multiple sclerosis. J. Clin. Invest. 129, 3277–3292 (2019). 31112527 10.1172/JCI124012 PMC6668690

[38] N. K. Tangudu, B. Alan, F. Vinchi, K. Worle, D. Lai, S. Vettorazzi, K. Leopold, M. Vujic Spasic, Scavenging reactive oxygen species production normalizes ferroportin expression and ameliorates cellular and systemic iron disbalances in hemolytic mouse model. Antioxid. Redox Signal. 29, 484–499 (2018). 29212341 10.1089/ars.2017.7089 PMC6034398

[39] M. R. Duggan, T. Mohseni Ahooyi, V. Parikh, K. Khalili, Neuromodulation of BAG co-chaperones by HIV-1 viral proteins and H_2_O_2_: Implications for HIV-associated neurological disorders. Cell Death Discov. 7, 60 (2021). 33771978 10.1038/s41420-021-00424-0 PMC7997901

[40] V. Jeney, J. Balla, A. Yachie, Z. Varga, G. M. Vercellotti, J. W. Eaton, G. Balla, Pro-oxidant and cytotoxic effects of circulating heme. Blood 100, 879–887 (2002). 12130498 10.1182/blood.v100.3.879

[41] S. Bathina, V. F. Lopez, M. Prado, E. Ballato, G. Colleluori, M. Tetlay, D. T. Villareal, S. Mediwala, R. Chen, C. Qualls, R. Armamento-Villareal, Health implications of racial differences in serum growth differentiation factor levels among men with obesity. Physiol. Rep. 12, e70124 (2024). 39668628 10.14814/phy2.70124 PMC11638490

[42] R. D. Semba, M. Gonzalez-Freire, T. Tanaka, A. Biancotto, P. Zhang, M. Shardell, R. Moaddel, CHI Consortium, L. Ferrucci, Elevated plasma growth and differentiation factor 15 is associated with slower gait speed and lower physical performance in healthy community-dwelling adults. J. Gerontol. A Biol. Sci. Med. Sci. 75, 175–180 (2020). 30874790 10.1093/gerona/glz071 PMC6909888

[43] S. Janelidze, E. Stomrud, R. Smith, S. Palmqvist, N. Mattsson, D. C. Airey, N. K. Proctor, X. Chai, S. Shcherbinin, J. R. Sims, G. Triana-Baltzer, C. Theunis, R. Slemmon, M. Mercken, H. Kolb, J. L. Dage, O. Hansson, Cerebrospinal fluid p-tau217 performs better than p-tau181 as a biomarker of Alzheimer's disease. Nat. Commun. 11, 1683 (2020). 32246036 10.1038/s41467-020-15436-0 PMC7125218

[44] M. Colonna, The biology of TREM receptors. Nat. Rev. Immunol. 23, 580–594 (2023). 36750615 10.1038/s41577-023-00837-1 PMC9904274

[45] M. M. Painter, Y. Atagi, C. C. Liu, R. Rademakers, H. Xu, J. D. Fryer, G. Bu, TREM2 in CNS homeostasis and neurodegenerative disease. Mol. Neurodegener. 10, 43 (2015). 26337043 10.1186/s13024-015-0040-9 PMC4560063

[46] Y. Shi, D. M. Holtzman, Interplay between innate immunity and Alzheimer disease: APOE and TREM2 in the spotlight. Nat. Rev. Immunol. 18, 759–772 (2018). 30140051 10.1038/s41577-018-0051-1 PMC6425488

[47] N. F. Fitz, K. N. Nam, C. M. Wolfe, F. Letronne, B. E. Playso, B. E. Iordanova, T. D. Y. Kozai, R. J. Biedrzycki, V. E. Kagan, Y. Y. Tyurina, X. Han, I. Lefterov, R. Koldamova, Phospholipids of APOE lipoproteins activate microglia in an isoform-specific manner in preclinical models of Alzheimer's disease. Nat. Commun. 12, 3416 (2021). 34099706 10.1038/s41467-021-23762-0 PMC8184801

[48] L. Duan, H. L. Pang, W. J. Chen, W. W. Shen, P. P. Cao, S. M. Wang, L. L. Liu, H. L. Zhang, The role of GDF15 in bone metastasis of lung adenocarcinoma cells. Oncol. Rep. 41, 2379–2388 (2019). 30816507 10.3892/or.2019.7024

[49] Y. Lu, J. Ma, Y. Li, J. Huang, S. Zhang, Z. Yin, J. Ren, K. Huang, G. Wu, K. Yang, S. Xu, CDP138 silencing inhibits TGF-beta/Smad signaling to impair radioresistance and metastasis via GDF15 in lung cancer. Cell Death Dis. 8, e3036 (2017). 28880265 10.1038/cddis.2017.434 PMC5636979

[50] M. P. Schreurs, M. J. Cipolla, Cerebrovascular dysfunction and blood-brain barrier permeability induced by oxidized LDL are prevented by apocynin and magnesium sulfate in female rats. J. Cardiovasc. Pharmacol. 63, 33–39 (2014). 24084218 10.1097/FJC.0000000000000021 PMC3909873

[51] D. Chiabrando, F. Vinchi, V. Fiorito, S. Mercurio, E. Tolosano, Heme in pathophysiology: A matter of scavenging, metabolism and trafficking across cell membranes. Front. Pharmacol. 5, 61 (2014). 24782769 10.3389/fphar.2014.00061 PMC3986552

[52] S. Lee, N. A. Devanney, L. R. Golden, C. T. Smith, J. L. Schwartz, A. E. Walsh, H. A. Clarke, D. S. Goulding, E. J. Allenger, G. Morillo-Segovia, C. M. Friday, A. A. Gorman, T. R. Hawkinson, S. M. MacLean, H. C. Williams, R. C. Sun, J. M. Morganti, L. A. Johnson, APOE modulates microglial immunometabolism in response to age, amyloid pathology, and inflammatory challenge. Cell Rep. 42, 112196 (2023). 36871219 10.1016/j.celrep.2023.112196 PMC10117631

[53] H. J. Chen, X. Dong, Y. Wang, K. Wang, G. Feng, T. Bai, M. Zhang, K. Gan, J. J. Peng, W. Huang, Z. Zhang, N. Shu, G. Ma, Polygenic risk for Alzheimer's disease in healthy aging: Age-related and APOE-driven effects on brain structures and cognition. Genome Med. 17, 126 (2025). 41121378 10.1186/s13073-025-01548-z PMC12539216

[54] E. M. Reiman, J. F. Arboleda-Velasquez, Y. T. Quiroz, M. J. Huentelman, T. G. Beach, R. J. Caselli, Y. Chen, Y. Su, A. J. Myers, J. Hardy, J. Paul Vonsattel, S. G. Younkin, D. A. Bennett, P. L. De Jager, E. B. Larson, P. K. Crane, C. D. Keene, M. I. Kamboh, J. K. Kofler, L. Duque, J. R. Gilbert, H. E. Gwirtsman, J. D. Buxbaum, D. W. Dickson, M. P. Frosch, B. F. Ghetti, K. L. Lunetta, L. S. Wang, B. T. Hyman, W. A. Kukull, T. Foroud, J. L. Haines, R. P. Mayeux, M. A. Pericak-Vance, J. A. Schneider, J. Q. Trojanowski, L. A. Farrer, G. D. Schellenberg, G. W. Beecham, T. J. Montine, G. R. Jun, Alzheimer’s Disease Genetics Consortium, Exceptionally low likelihood of Alzheimer’s dementia in APOE2 homozygotes from a 5,000-person neuropathological study. Nat. Commun. 11, 667 (2020). 32015339 10.1038/s41467-019-14279-8 PMC6997393

[55] M. R. Rooney, J. Chen, C. M. Ballantyne, R. C. Hoogeveen, O. Tang, M. E. Grams, A. Tin, C. E. Ndumele, F. Zannad, D. J. Couper, W. Tang, E. Selvin, J. Coresh, Comparison of proteomic measurements across platforms in the Atherosclerosis Risk in Communities (ARIC) Study. Clin. Chem. 69, 68–79 (2023). 36508319 10.1093/clinchem/hvac186 PMC9812856

[56] T. Tanaka, A. Biancotto, R. Moaddel, A. Z. Moore, M. Gonzalez-Freire, M. A. Aon, J. Candia, P. Zhang, F. Cheung, G. Fantoni, CHI consortium, R. D. Semba, L. Ferrucci, Plasma proteomic signature of age in healthy humans. Aging Cell 17, e12799 (2018). 29992704 10.1111/acel.12799 PMC6156492

[57] G. H. Eldjarn, E. Ferkingstad, S. H. Lund, H. Helgason, O. T. Magnusson, K. Gunnarsdottir, T. A. Olafsdottir, B. V. Halldorsson, P. I. Olason, F. Zink, S. A. Gudjonsson, G. Sveinbjornsson, M. I. Magnusson, A. Helgason, A. Oddsson, G. H. Halldorsson, M. K. Magnusson, S. Saevarsdottir, T. Eiriksdottir, G. Masson, H. Stefansson, I. Jonsdottir, H. Holm, T. Rafnar, P. Melsted, J. Saemundsdottir, G. L. Norddahl, G. Thorleifsson, M. O. Ulfarsson, D. F. Gudbjartsson, U. Thorsteinsdottir, P. Sulem, K. Stefansson, Large-scale plasma proteomics comparisons through genetics and disease associations. Nature 622, 348–358 (2023). 37794188 10.1038/s41586-023-06563-x PMC10567571

[58] D. H. Katz, J. M. Robbins, S. Deng, U. A. Tahir, A. G. Bick, A. Pampana, Z. Yu, D. Ngo, M. D. Benson, Z. Z. Chen, D. E. Cruz, D. Shen, Y. Gao, C. Bouchard, M. A. Sarzynski, A. Correa, P. Natarajan, J. G. Wilson, R. E. Gerszten, Proteomic profiling platforms head to head: Leveraging genetics and clinical traits to compare aptamer- and antibody-based methods. Sci. Adv. 8, eabm5164 (2022). 35984888 10.1126/sciadv.abm5164 PMC9390994

[59] M. Pietzner, E. Wheeler, J. Carrasco-Zanini, N. D. Kerrison, E. Oerton, M. Koprulu, J. Luan, A. D. Hingorani, S. A. Williams, N. J. Wareham, C. Langenberg, Synergistic insights into human health from aptamer- and antibody-based proteomic profiling. Nat. Commun. 12, 6822 (2021). 34819519 10.1038/s41467-021-27164-0 PMC8613205

[60] J. D. Wright, A. R. Folsom, J. Coresh, A. R. Sharrett, D. Couper, L. E. Wagenknecht, T. H. Mosley Jr., C. M. Ballantyne, E. A. Boerwinkle, W. D. Rosamond, G. Heiss, The ARIC (Atherosclerosis Risk In Communities) Study: JACC Focus Seminar 3/8. J. Am. Coll. Cardiol. 77, 2939–2959 (2021). 34112321 10.1016/j.jacc.2021.04.035 PMC8667593

[61] A. Tin, B. Yu, J. Ma, K. Masushita, N. Daya, R. C. Hoogeveen, C. M. Ballantyne, D. Couper, C. M. Rebholz, M. E. Grams, A. Alonso, T. Mosley, G. Heiss, P. Ganz, E. Selvin, E. Boerwinkle, J. Coresh, Reproducibility and variability of protein analytes measured using a multiplexed modified aptamer assay. J. Appl. Lab. Med. 4, 30–39 (2019). 31639705 10.1373/jalm.2018.027086 PMC6814271

[62] C. R. Carpenter, B. DesPain, T. N. Keeling, M. Shah, M. Rothenberger, The Six-Item Screener and AD8 for the detection of cognitive impairment in geriatric emergency department patients. Ann. Emerg. Med. 57, 653–661 (2011). 20855129 10.1016/j.annemergmed.2010.06.560 PMC3213856

[63] J. E. Galvin, C. M. Roe, C. Xiong, J. C. Morris, Validity and reliability of the AD8 informat interview in dementia. Neurology 67, 1942–1948 (2006). 17159098 10.1212/01.wnl.0000247042.15547.eb

[64] K. A. Walker, A. R. Sharrett, A. Wu, A. L. C. Schneider, M. Albert, P. L. Lutsey, K. Bandeen-Roche, J. Coresh, A. L. Gross, B. G. Windham, D. S. Knopman, M. C. Power, A. M. Rawlings, T. H. Mosley, R. F. Gottesman, Association of midlife to late-life blood pressure patterns with incident dementia. JAMA 322, 535–545 (2019). 31408138 10.1001/jama.2019.10575 PMC6692677

[65] K. M. Hayden, B. R. Reed, J. J. Manly, D. Tommet, R. H. Pietrzak, G. J. Chelune, F. M. Yang, A. J. Revell, D. A. Bennett, R. N. Jones, Cognitive decline in the elderly: An analysis of population heterogeneity. Age Ageing 40, 684–689 (2011). 21890481 10.1093/ageing/afr101 PMC3199215

[66] R. S. Wilson, Y. Li, J. L. Bienias, D. A. Bennett, Cognitive decline in old age: Separating retest effects from the effects of growing older. Psychol. Aging 21, 774–789 (2006). 17201497 10.1037/0882-7974.21.4.774

[67] R. F. Gottesman, M. S. Albert, A. Alonso, L. H. Coker, J. Coresh, S. M. Davis, J. A. Deal, G. M. McKhann, T. H. Mosley, A. R. Sharrett, A. L. C. Schneider, B. G. Windham, L. M. Wruck, D. S. Knopman, Associations between midlife vascular risk factors and 25-year incident dementia in the Atherosclerosis Risk in Communities (ARIC) cohort. JAMA Neurol. 74, 1246–1254 (2017). 28783817 10.1001/jamaneurol.2017.1658 PMC5710244

[68] A. L. C. Schneider, E. Selvin, A. R. Sharrett, M. Griswold, J. Coresh, C. R. Jack Jr., D. Knopman, T. Mosley, R. F. Gottesman, Diabetes, prediabetes, and brain volumes and subclinical cerebrovascular disease on MRI: The Atherosclerosis Risk in Communities Neurocognitive Study (ARIC-NCS). Diabetes Care 40, 1514–1521 (2017). 28916531 10.2337/dc17-1185 PMC5652590

[69] B. Fischl, A. M. Dale, Measuring the thickness of the human cerebral cortex from magnetic resonance images. Proc. Natl. Acad. Sci. U.S.A. 97, 11050–11055 (2000). 10984517 10.1073/pnas.200033797 PMC27146

[70] B. Fischl, D. H. Salat, E. Busa, M. Albert, M. Dieterich, C. Haselgrove, A. van der Kouwe, R. Killiany, D. Kennedy, S. Klaveness, A. Montillo, N. Makris, B. Rosen, A. M. Dale, Whole brain segmentation: Automated labeling of neuroanatomical structures in the human brain. Neuron 33, 341–355 (2002). 11832223 10.1016/s0896-6273(02)00569-x

[71] C. R. Jack Jr., P. C. O’Brien, D. W. Rettman, M. M. Shiung, Y. Xu, R. Muthupillai, A. Manduca, R. Avula, B. J. Erickson, FLAIR histogram segmentation for measurement of leukoaraiosis volume. J. Magn. Reson. Imaging 14, 668–676 (2001). 11747022 10.1002/jmri.10011 PMC2755497

[72] L. Raz, M. Jayachandran, N. Tosakulwong, T. G. Lesnick, S. M. Wille, M. C. Murphy, M. L. Senjem, J. L. Gunter, P. Vemuri, C. R. Jack Jr., V. M. Miller, K. Kantarci, Thrombogenic microvesicles and white matter hyperintensities in postmenopausal women. Neurology 80, 911–918 (2013). 23408873 10.1212/WNL.0b013e3182840c9f PMC3653211

[73] R. F. Gottesman, A. L. Schneider, Y. Zhou, X. Chen, E. Green, N. Gupta, D. S. Knopman, A. Mintz, A. Rahmim, A. R. Sharrett, L. E. Wagenknecht, D. F. Wong, T. H. Mosley Jr., The ARIC-PET amyloid imaging study: Brain amyloid differences by age, race, sex, and APOE. Neurology 87, 473–480 (2016). 27371485 10.1212/WNL.0000000000002914 PMC4970663

[74] A. S. Levey, L. A. Stevens, C. H. Schmid, Y. L. Zhang, A. F. Castro III, H. I. Feldman, J. W. Kusek, P. Eggers, F. Van Lente, T. Greene, J. Coresh, CKD-EPI (Chronic Kidney Disease Epidemiology Collaboration), A new equation to estimate glomerular filtration rate. Ann. Intern. Med. 150, 604–612 (2009). 19414839 10.7326/0003-4819-150-9-200905050-00006 PMC2763564

[75] B. B. Sun, J. Chiou, M. Traylor, C. Benner, Y. H. Hsu, T. G. Richardson, P. Surendran, A. Mahajan, C. Robins, S. G. Vasquez-Grinnell, L. Hou, E. M. Kvikstad, O. S. Burren, J. Davitte, K. L. Ferber, C. E. Gillies, A. K. Hedman, S. Hu, T. Lin, R. Mikkilineni, R. K. Pendergrass, C. Pickering, B. Prins, D. Baird, C. Y. Chen, L. D. Ward, A. M. Deaton, S. Welsh, C. M. Willis, N. Lehner, M. Arnold, M. A. Worheide, K. Suhre, G. Kastenmuller, A. Sethi, M. Cule, A. Raj, Alnylam Human Genetics, AstraZeneca Genomics Initiative, Biogen Biobank Team, Bristol Myers Squibb, Genentech Human Genetics, GlaxoSmithKline Genomic Sciences, Pfizer Integrative Biology, Population Analytics of Janssen Data Sciences, Regeneron Genetics Center, L. Burkitt-Gray, E. Melamud, M. H. Black, E. B. Fauman, J. M. M. Howson, H. M. Kang, M. I. McCarthy, P. Nioi, S. Petrovski, R. A. Scott, E. N. Smith, S. Szalma, D. M. Waterworth, L. J. Mitnaul, J. D. Szustakowski, B. W. Gibson, M. R. Miller, C. D. Whelan, Plasma proteomic associations with genetics and health in the UK Biobank. Nature 622, 329–338 (2023). 37794186 10.1038/s41586-023-06592-6 PMC10567551

[76] F. Casanova, Q. Tian, D. S. Williamson, Y. Qian, D. Zweibaum, J. Ding, J. L. Atkins, D. Melzer, L. Ferrucci, L. C. Pilling, MRI-derived brain iron, grey matter volume, and risk of dementia and Parkinson’s disease: Observational and genetic analysis in the UK Biobank cohort. Neurobiol. Dis. 197, 106539 (2024). 38789058 10.1016/j.nbd.2024.106539 PMC12048010

[77] F. Alfaro-Almagro, M. Jenkinson, N. K. Bangerter, J. L. R. Andersson, L. Griffanti, G. Douaud, S. N. Sotiropoulos, S. Jbabdi, M. Hernandez-Fernandez, E. Vallee, D. Vidaurre, M. Webster, P. McCarthy, C. Rorden, A. Daducci, D. C. Alexander, H. Zhang, I. Dragonu, P. M. Matthews, K. L. Miller, S. M. Smith, Image processing and quality control for the first 10,000 brain imaging datasets from UK Biobank. Neuroimage 166, 400–424 (2018). 29079522 10.1016/j.neuroimage.2017.10.034 PMC5770339

[78] C. N. Spracklen, P. Chen, Y. J. Kim, X. Wang, H. Cai, S. Li, J. Long, Y. Wu, Y. X. Wang, F. Takeuchi, J. Y. Wu, K. J. Jung, C. Hu, K. Akiyama, Y. Zhang, S. Moon, T. A. Johnson, H. Li, R. Dorajoo, M. He, M. E. Cannon, T. S. Roman, E. Salfati, K. H. Lin, X. Guo, W. H. H. Sheu, D. Absher, L. S. Adair, T. L. Assimes, T. Aung, Q. Cai, L. C. Chang, C. H. Chen, L. H. Chien, L. M. Chuang, S. C. Chuang, S. Du, Q. Fan, C. S. J. Fann, A. B. Feranil, Y. Friedlander, P. Gordon-Larsen, D. Gu, L. Gui, Z. Guo, C. K. Heng, J. Hixson, X. Hou, C. A. Hsiung, Y. Hu, M. Y. Hwang, C. M. Hwu, M. Isono, J. J. Juang, C. C. Khor, Y. K. Kim, W. P. Koh, M. Kubo, I. T. Lee, S. J. Lee, W. J. Lee, K. W. Liang, B. Lim, S. H. Lim, J. Liu, T. Nabika, W. H. Pan, H. Peng, T. Quertermous, C. Sabanayagam, K. Sandow, J. Shi, L. Sun, P. C. Tan, S. P. Tan, K. D. Taylor, Y. Y. Teo, S. A. Toh, T. Tsunoda, R. M. van Dam, A. Wang, F. Wang, J. Wang, W. B. Wei, Y. B. Xiang, J. Yao, J. M. Yuan, R. Zhang, W. Zhao, Y. I. Chen, S. S. Rich, J. I. Rotter, T. D. Wang, T. Wu, X. Lin, B. G. Han, T. Tanaka, Y. S. Cho, T. Katsuya, W. Jia, S. H. Jee, Y. T. Chen, N. Kato, J. B. Jonas, C. Y. Cheng, X. O. Shu, J. He, W. Zheng, T. Y. Wong, W. Huang, B. J. Kim, E. S. Tai, K. L. Mohlke, X. Sim, Association analyses of East Asian individuals and trans-ancestry analyses with European individuals reveal new loci associated with cholesterol and triglyceride levels. Hum. Mol. Genet. 26, 1770–1784 (2017). 28334899 10.1093/hmg/ddx062 PMC6075203

[79] T. B. Harris, L. J. Launer, G. Eiriksdottir, O. Kjartansson, P. V. Jonsson, G. Sigurdsson, G. Thorgeirsson, T. Aspelund, M. E. Garcia, M. F. Cotch, H. J. Hoffman, V. Gudnason, Age, Gene/Environment Susceptibility-Reykjavik Study: Multidisciplinary applied phenomics. Am. J. Epidemiol. 165, 1076–1087 (2007). 17351290 10.1093/aje/kwk115 PMC2723948

[80] A. S. Levey, J. Coresh, T. Greene, L. A. Stevens, Y. L. Zhang, S. Hendriksen, J. W. Kusek, F. Van Lente, Chronic Kidney Disease Epidemiology Collaboration, Using standardized serum creatinine values in the modification of diet in renal disease study equation for estimating glomerular filtration rate. Ann. Intern. Med. 145, 247–254 (2006). 16908915 10.7326/0003-4819-145-4-200608150-00004

[81] N. W. Shock, R. C. Greulich, R. Andres, D. Arenberg, J. P. T. Costa, E. G. Lakatta, J. D. Tobin, Normal human aging: The Baltimore Longitudinal Study of Aging. J. Gerontol. 40, 767 (1984).

[82] L. Ferrucci, The Baltimore Longitudinal Study of Aging (BLSA): A 50-year-long journey and plans for the future. J. Gerontology. A Biol. Med. Sci. 63, 1416–1419 (2008). 10.1093/gerona/63.12.1416 PMC5004590 19126858

[83] S. M. Resnick, D. L. Pham, M. A. Kraut, A. B. Zonderman, C. Davatzikos, Longitudinal magnetic resonance imaging studies of older adults: A shrinking brain. J. Neurosci. 23, 3295–3301 (2003). 12716936 10.1523/JNEUROSCI.23-08-03295.2003 PMC6742337

[84] J. Candia, G. N. Daya, T. Tanaka, L. Ferrucci, K. A. Walker, Assessment of variability in the plasma 7k SomaScan proteomics assay. Sci. Rep. 12, 17147 (2022). 36229504 10.1038/s41598-022-22116-0 PMC9561184

[85] J. Doshi, G. Erus, Y. Ou, S. M. Resnick, R. C. Gur, R. E. Gur, T. D. Satterthwaite, S. Furth, C. Davatzikos, I. Alzheimer’s Neuroimaging, MUSE: MUlti-atlas region Segmentation utilizing Ensembles of registration algorithms and parameters, and locally optimal atlas selection. Neuroimage 127, 186–195 (2016). 26679328 10.1016/j.neuroimage.2015.11.073 PMC4806537

[86] C. Davatzikos, A. Genc, D. Xu, S. M. Resnick, Voxel-based morphometry using the RAVENS maps: Methods and validation using simulated longitudinal atrophy. Neuroimage 14, 1361–1369 (2001). 11707092 10.1006/nimg.2001.0937

[87] X. Da, J. B. Toledo, J. Zee, D. A. Wolk, S. X. Xie, Y. Ou, A. Shacklett, P. Parmpi, L. Shaw, J. Q. Trojanowski, C. Davatzikos, I. Alzheimer’s Neuroimaging, Integration and relative value of biomarkers for prediction of MCI to AD progression: Spatial patterns of brain atrophy, cognitive scores, APOE genotype and CSF biomarkers. Neuroimage Clin. 4, 164–173 (2014). 24371799 10.1016/j.nicl.2013.11.010 PMC3871290

[88] C. Davatzikos, P. Bhatt, L. M. Shaw, K. N. Batmanghelich, J. Q. Trojanowski, Prediction of MCI to AD conversion, via MRI, CSF biomarkers, and pattern classification. Neurobiol. Aging 32, 2322 2322.e19–2322.e27 (2011). 10.1016/j.neurobiolaging.2010.05.023 PMC2951483 20594615

[89] C. Davatzikos, S. M. Resnick, X. Wu, P. Parmpi, C. M. Clark, Individual patient diagnosis of AD and FTD via high-dimensional pattern classification of MRI. Neuroimage 41, 1220–1227 (2008). 18474436 10.1016/j.neuroimage.2008.03.050 PMC2528893

[90] C. Davatzikos, F. Xu, Y. An, Y. Fan, S. M. Resnick, Longitudinal progression of Alzheimer’s-like patterns of atrophy in normal older adults: The SPARE-AD index. Brain 132, 2026–2035 (2009). 19416949 10.1093/brain/awp091 PMC2714059

[91] Y. Fan, N. Batmanghelich, C. M. Clark, C. Davatzikos, Alzheimer’s Disease Neuroimaging Initiative, Spatial patterns of brain atrophy in MCI patients, identified via high-dimensional pattern classification, predict subsequent cognitive decline. Neuroimage 39, 1731–1743 (2008). 18053747 10.1016/j.neuroimage.2007.10.031 PMC2861339

[92] J. B. Toledo, M. W. Weiner, D. A. Wolk, X. Da, K. Chen, S. E. Arnold, W. Jagust, C. Jack, E. M. Reiman, C. Davatzikos, L. M. Shaw, J. Q. Trojanowski, Alzheimer’s Disease Neuroimaging Initiative, Neuronal injury biomarkers and prognosis in ADNI subjects with normal cognition. Acta Neuropathol. Commun. 2, 26 (2014). 24602322 10.1186/2051-5960-2-26 PMC4008258

[93] M. Habes, G. Erus, J. B. Toledo, T. Zhang, N. Bryan, L. J. Launer, Y. Rosseel, D. Janowitz, J. Doshi, S. Van der Auwera, B. von Sarnowski, K. Hegenscheid, N. Hosten, G. Homuth, H. Volzke, U. Schminke, W. Hoffmann, H. J. Grabe, C. Davatzikos, White matter hyperintensities and imaging patterns of brain ageing in the general population. Brain 139, 1164–1179 (2016). 26912649 10.1093/brain/aww008 PMC5006227

[94] M. Bilgel, Y. An, K. A. Walker, A. R. Moghekar, N. J. Ashton, P. R. Kac, T. K. Karikari, K. Blennow, H. Zetterberg, B. M. Jedynak, M. Thambisetty, L. Ferrucci, S. M. Resnick, Longitudinal changes in Alzheimer’s-related plasma biomarkers and brain amyloid. Alzheimers Dement. 19, 4335–4345 (2023). 37216632 10.1002/alz.13157 PMC10592628

[95] M. E. Levine, A. T. Lu, A. Quach, B. H. Chen, T. L. Assimes, S. Bandinelli, L. Hou, A. A. Baccarelli, J. D. Stewart, Y. Li, E. A. Whitsel, J. G. Wilson, A. P. Reiner, A. Aviv, K. Lohman, Y. Liu, L. Ferrucci, S. Horvath, An epigenetic biomarker of aging for lifespan and healthspan. Aging 10, 573–591 (2018). 29676998 10.18632/aging.101414 PMC5940111

[96] P. L. Kuo, A. Z. Moore, F. R. Lin, L. Ferrucci, Epigenetic age acceleration and hearing: Observations from the Baltimore Longitudinal Study of Aging. Front. Aging Neurosci. 13, 790926 (2021). 34975461 10.3389/fnagi.2021.790926 PMC8714776

[97] L. A. Inker, N. D. Eneanya, J. Coresh, H. Tighiouart, D. Wang, Y. Sang, D. C. Crews, A. Doria, M. M. Estrella, M. Froissart, M. E. Grams, T. Greene, A. Grubb, V. Gudnason, O. M. Gutierrez, R. Kalil, A. B. Karger, M. Mauer, G. Navis, R. G. Nelson, E. D. Poggio, R. Rodby, P. Rossing, A. D. Rule, E. Selvin, J. C. Seegmiller, M. G. Shlipak, V. E. Torres, W. Yang, S. H. Ballew, S. J. Couture, N. R. Powe, A. S. Levey, Chronic Kidney Disease Epidemiology Collaboration, New creatinine- and cystatin C-based equations to estimate GFR without race. N. Engl. J. Med. 385, 1737–1749 (2021). 34554658 10.1056/NEJMoa2102953 PMC8822996

[98] M. R. Duggan, Z. Peng, Y. An, M. H. Kitner-Triolo, A. T. Shafer, C. Davatzikos, G. Erus, A. Karikkineth, A. Lewis, A. Moghekar, K. A. Walker, Herpes viruses in the Baltimore Longitudinal Study of Aging: Associations with brain volumes, cognitive performance, and plasma biomarkers. Neurology 99, e2014–e2024 (2022). 35985823 10.1212/WNL.0000000000201036 PMC9651463

[99] E. Y. Chen, C. M. Tan, Y. Kou, Q. Duan, Z. Wang, G. V. Meirelles, N. R. Clark, A. Ma’ayan, Enrichr: Interactive and collaborative HTML5 gene list enrichment analysis tool. BMC Bioinformatics 14, 128 (2013). 23586463 10.1186/1471-2105-14-128 PMC3637064

[100] M. V. Kuleshov, M. R. Jones, A. D. Rouillard, N. F. Fernandez, Q. Duan, Z. Wang, S. Koplev, S. L. Jenkins, K. M. Jagodnik, A. Lachmann, M. G. McDermott, C. D. Monteiro, G. W. Gundersen, A. Ma’ayan, Enrichr: A comprehensive gene set enrichment analysis web server 2016 update. Nucleic Acids Res. 44, W90–W97 (2016). 27141961 10.1093/nar/gkw377 PMC4987924

[101] Z. Xie, A. Bailey, M. V. Kuleshov, D. J. B. Clarke, J. E. Evangelista, S. L. Jenkins, A. Lachmann, M. L. Wojciechowicz, E. Kropiwnicki, K. M. Jagodnik, M. Jeon, A. Ma’ayan, Gene set knowledge discovery with Enrichr. Curr. Protoc. 1, e90 (2021). 33780170 10.1002/cpz1.90 PMC8152575

[102] D. Wessel, U. I. Flugge, A method for the quantitative recovery of protein in dilute solution in the presence of detergents and lipids. Anal. Biochem. 138, 141–143 (1984). 6731838 10.1016/0003-2697(84)90782-6

[103] Y. Wang, F. Yang, M. A. Gritsenko, Y. Wang, T. Clauss, T. Liu, Y. Shen, M. E. Monroe, D. Lopez-Ferrer, T. Reno, R. J. Moore, R. L. Klemke, D. G. Camp II, R. D. Smith, Reversed-phase chromatography with multiple fraction concatenation strategy for proteome profiling of human MCF10A cells. Proteomics 11, 2019–2026 (2011). 21500348 10.1002/pmic.201000722 PMC3120047

[104] X. Zhang, A. H. Smits, G. B. van Tilburg, H. Ovaa, W. Huber, M. Vermeulen, Proteome-wide identification of ubiquitin interactions using UbIA-MS. Nat. Protoc. 13, 530–550 (2018). 29446774 10.1038/nprot.2017.147

[105] S. Purcell, B. Neale, K. Todd-Brown, L. Thomas, M. A. Ferreira, D. Bender, J. Maller, P. Sklar, P. I. de Bakker, M. J. Daly, P. C. Sham, PLINK: A tool set for whole-genome association and population-based linkage analyses. Am. J. Hum. Genet. 81, 559–575 (2007). 17701901 10.1086/519795 PMC1950838

[106] J. Yang, T. Ferreira, A. P. Morris, S. E. Medland, P. A. F. Madden, A. C. Heath, N. G. Martin, G. W. Montgomery, M. N. Weedon, R. J. F. Loos, T. M. Frayling, M. I. McCarthy, J. N. Hirschhorn, M. E. Goddard, P. M. V. Visscher, Conditional and joint multiple-SNP analysis of GWAS summary statistics identifies additional variants influencing complex traits. Nat. Genet. 44, 369–375 (2012). 22426310 10.1038/ng.2213 PMC3593158

[107] L. Jiang, Z. Zheng, H. Fang, J. Yang, A generalized linear mixed model association tool for biobank-scale data. Nat. Genet. 53, 1616–1621 (2021). 34737426 10.1038/s41588-021-00954-4

[108] L. Jiang, Z. Zheng, T. Qi, K. E. Kemper, N. R. Wray, P. M. Visscher, J. Yang, A resource-efficient tool for mixed model association analysis of large-scale data. Nat. Genet. 51, 1749–1755 (2019). 31768069 10.1038/s41588-019-0530-8

