# Supplementary material for: Plasma GDF15 affects long-term dementia risk and alters neuroimmune signaling
Source: Sci Adv. 2026 Jun 26;12(26):eaec7614. doi: 10.1126/sciadv.aec7614 (PMC13308606; doi:10.1126/sciadv.aec7614)
Supplement: Supplementary file 1 — Figs. S1 to S16 Legends for tables S1 to S24 [file sciadv.aec7614_sm.pdf]

Science Advances
AAAS

Supplementary Materials for

Plasma GDF15 affects long-term dementia risk and alters neuroimmune signaling

Cassandra O. Blew *et al.*

Corresponding author: Keenan A. Walker, keenan.walker@nih.gov

Sci. Adv. **12**, eaec7614 (2026)
DOI: 10.1126/sciadv.aec7614

The PDF file includes:

Figs. S1 to S16
Legends for tables S1 to S24

Other Supplementary Material for this manuscript includes the following:

Tables S1 to S24

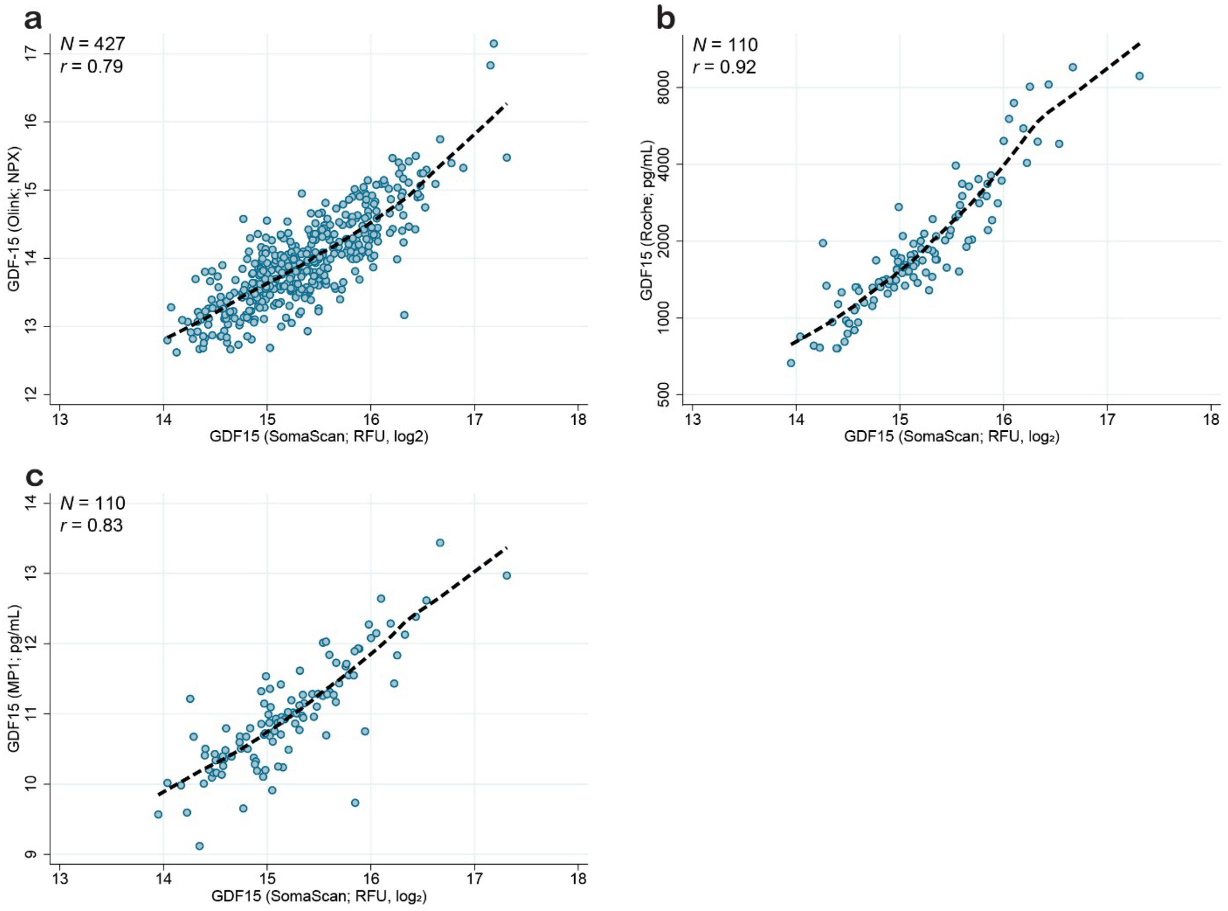

Fig. S1. Plasma GDF15 measurement validation.

External validation of plasma GDF15 measurements on the SomaScan platform in ARIC using **a.** the Olink platform, **b.** the Roche platform, and **c.** the Luminex platform. Results are unadjusted Spearman correlations; lines shown are LOESS curves.

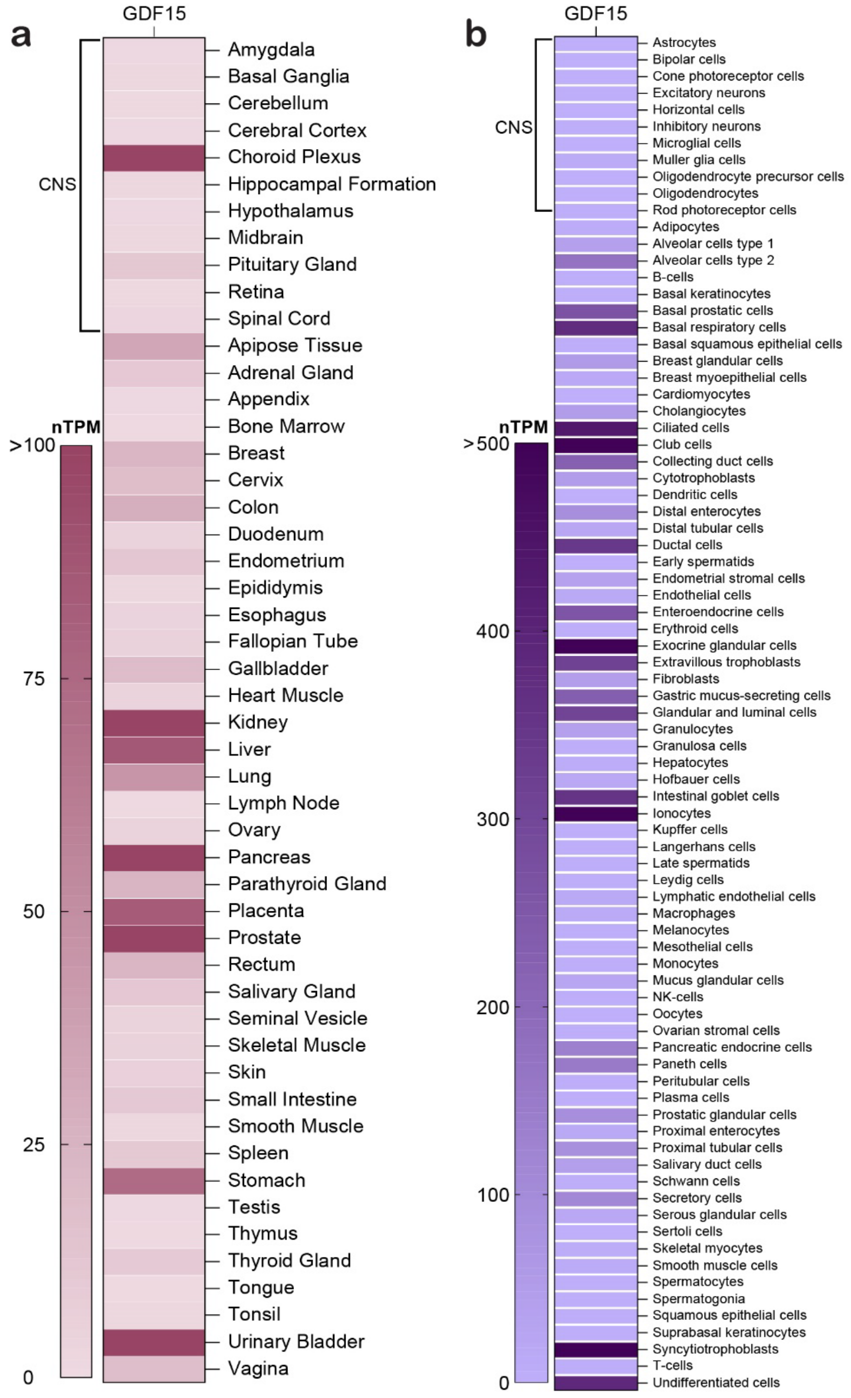

Fig. S2. GDF15 expression in cell and tissue types.

a. Expression of GDF15 in 50 different tissue types; data obtained from the Human Protein Atlas (*15*, *16*). Values greater than 500 normalized transcripts per million (nTPM) were capped for illustrative purposes. **b.** Expression of GDF15 in 76 different cell types; data obtained from the Human Protein Atlas (*15*, *16*). Values greater than 100 nTPM were capped for illustrative purposes. See **table S3** for raw data.

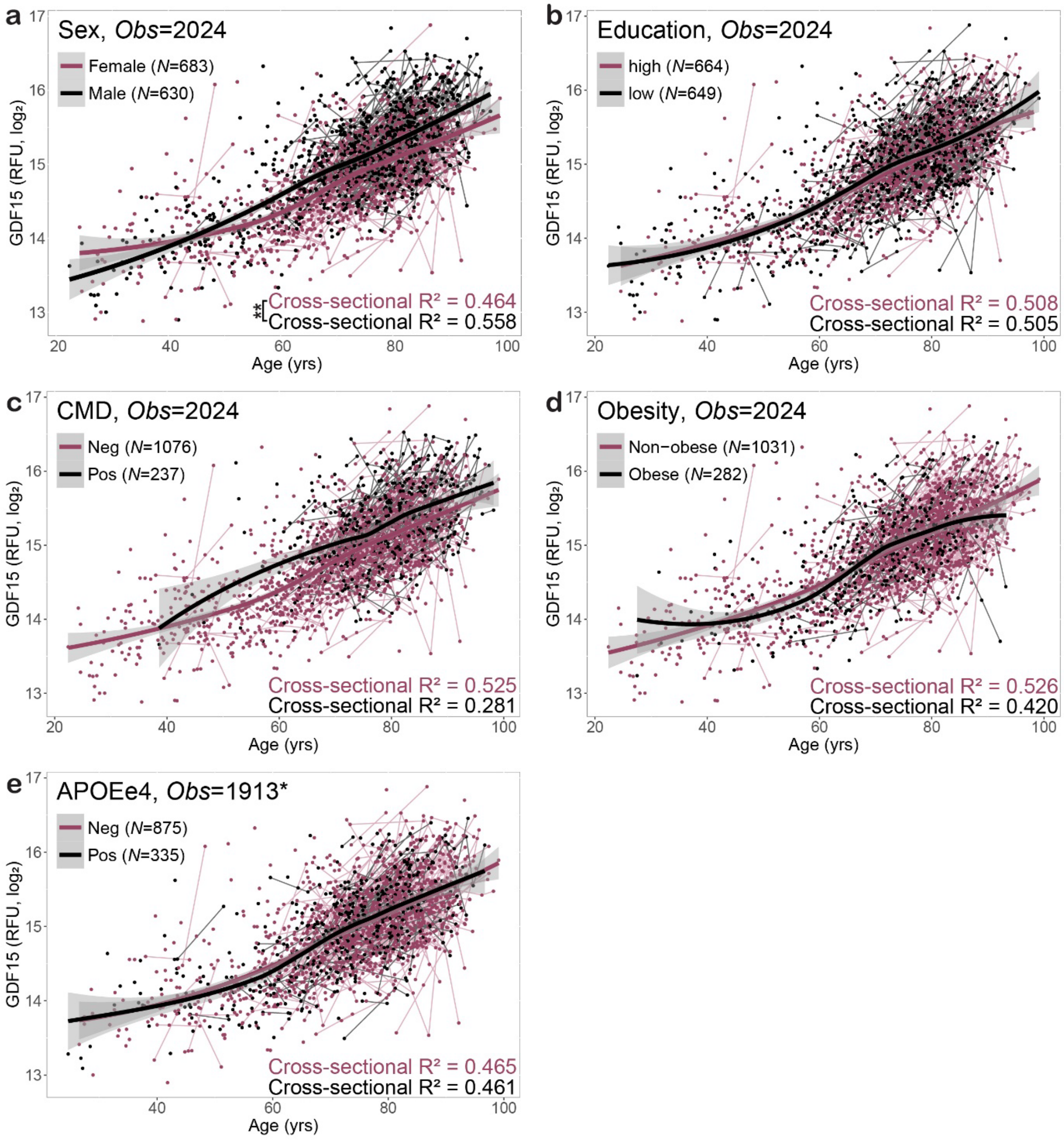

Fig. S3. Plasma GDF15 associations with age among all participants (ages 22-99) in the Baltimore Longitudinal Study of Aging (BLSA).

Associations of plasma GDF15 with age stratified by **a.** sex, **b.** educational attainment, **c.** cardiometabolic disease, **d.** obesity, and **e.** AD genetic risk (APOEε4 status; *Includes fewer observations due to unknown genotype). Scatter plots show multiple visits from the same participants, if applicable, connected by small lines; larger lines depict stratum-specific trends fitted using locally estimated scatterplot smoothing (LOESS), a nonparametric regression method that fits low-degree polynomials to localized subsets of the data. Cross-sectional statistics were obtained from unadjusted linear regression models which used the earliest available blood sample per participant.

Abbreviations: AD, Alzheimer's disease; CMD, cardiometabolic disease; Obs, observations

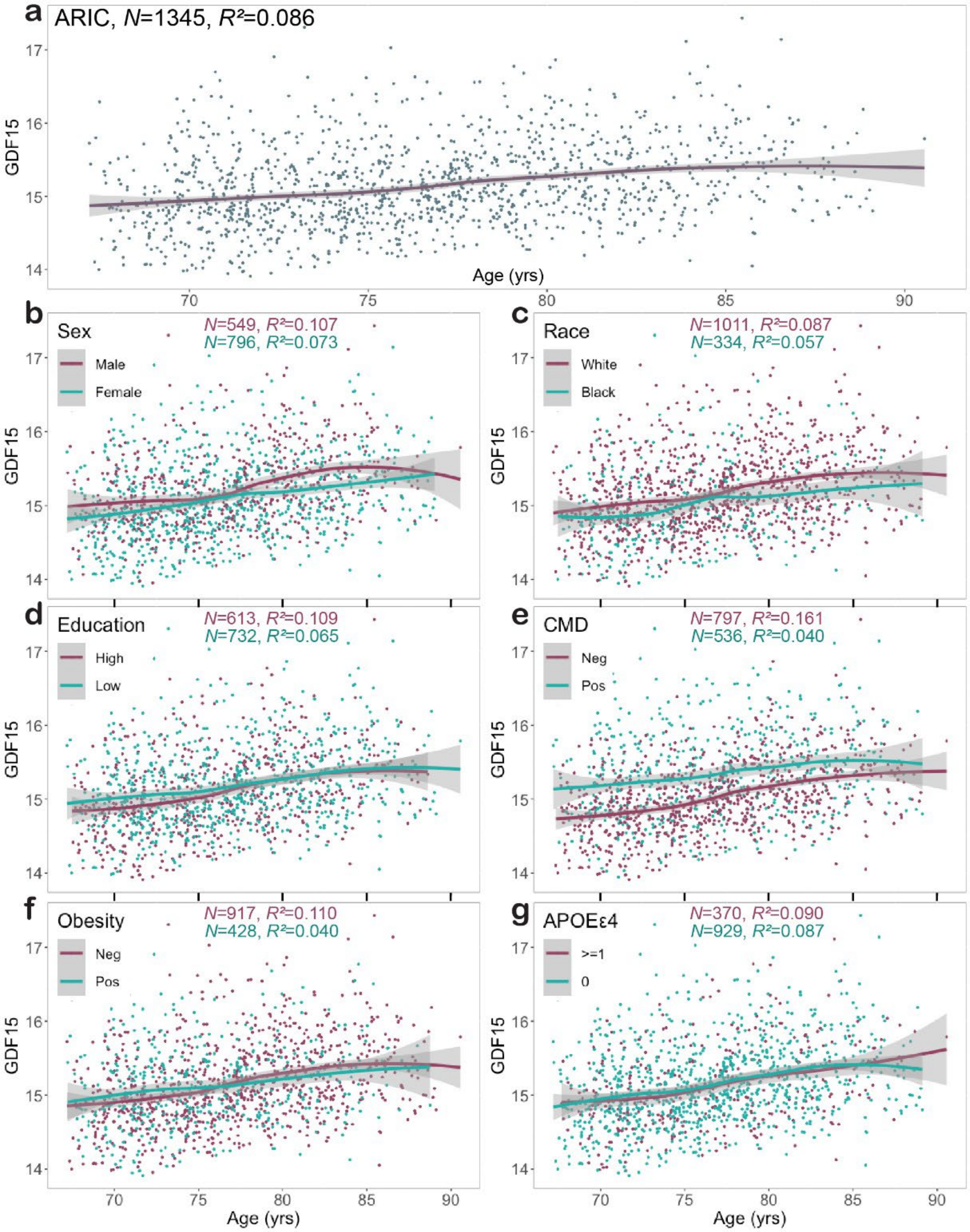

Fig. S4. Plasma GDF15 associations with age among individuals 65 and older from the Atherosclerosis Risk in Communities (ARIC) study.

Associations of **a.** plasma GDF15 with age, and associations of plasma GDF15 with age stratified by **b.** sex, **c.** race, **d.** educational attainment, **e.** cardiometabolic disease, **f.** obesity, and **g.** AD genetic risk (*APOEε4* status). Units of GDF15 are $\log_2$-transformed relative fluorescence units (RFU). Scatter plots show multiple visits from the same participants, if applicable, connected by small lines; larger lines depict stratum-specific trends fitted using locally estimated scatterplot smoothing (LOESS), a nonparametric regression method that fits low-degree polynomials to localized subsets of the data. Results derived from unadjusted linear regression models.

Abbreviations: CMD, cardiometabolic disease.

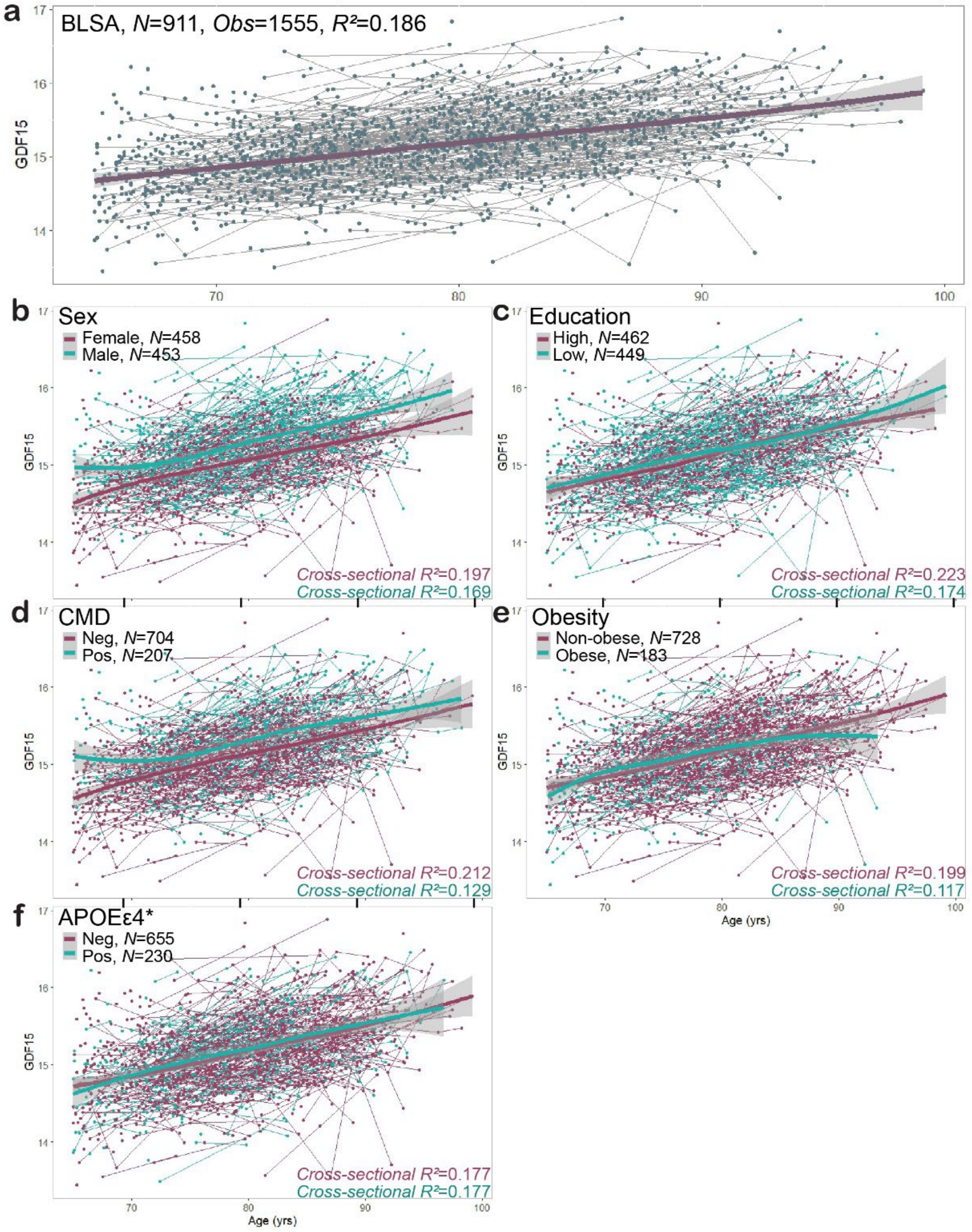

Fig. S5. Plasma GDF15 associations with age among individuals 65 and older from the Baltimore Longitudinal Study of Aging (BLSA).

Associations of **a.** plasma GDF15 with age, and associations of plasma GDF15 with age stratified by **b.** sex, **c.** educational attainment, **d.** cardiometabolic disease, **e.** obesity, and **f.** AD genetic risk (APOEε4 status); *Includes fewer participants due to unknown genotype. Units of GDF15 are log2-transformed relative fluorescence units (RFU). Scatter plots show multiple visits from the same participants, if applicable, connected by small lines; larger lines depict stratum-specific trends fitted using locally estimated scatterplot smoothing (LOESS), a nonparametric regression method that fits low-degree polynomials to localized subsets of the data. Cross-sectional statistics were obtained from unadjusted linear regression models which used the earliest available blood sample per participant.

Abbreviations: CMD, cardiometabolic disease.

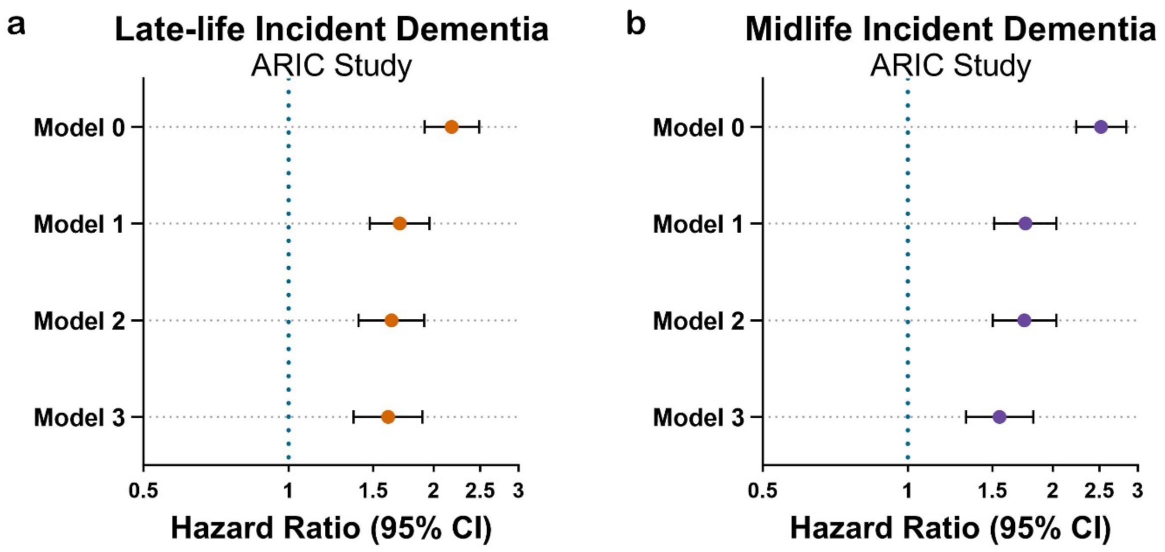

Fig. S6. Plasma GDF15 associations with all-cause dementia (ACD) risk among individuals from the Atherosclerosis Risk in Communities (ARIC) study.

Associations of **a.** plasma GDF15 with near term (7-year) ACD risk using plasma collected in late-life, and **b.** plasma GDF15 with long term (20-year) ACD risk using plasma collected in midlife. Results derived from Cox proportional hazards regression models. Model 0 was unadjusted.

Model 1 adjusted for age, race-center, sex, education, and *APOEε4* status.

Model 2 adjusted for Model 1 covariates plus eGFR.

Model 3 adjusted for Model 2 covariates plus BMI, diabetes, hypertension, and smoking status.

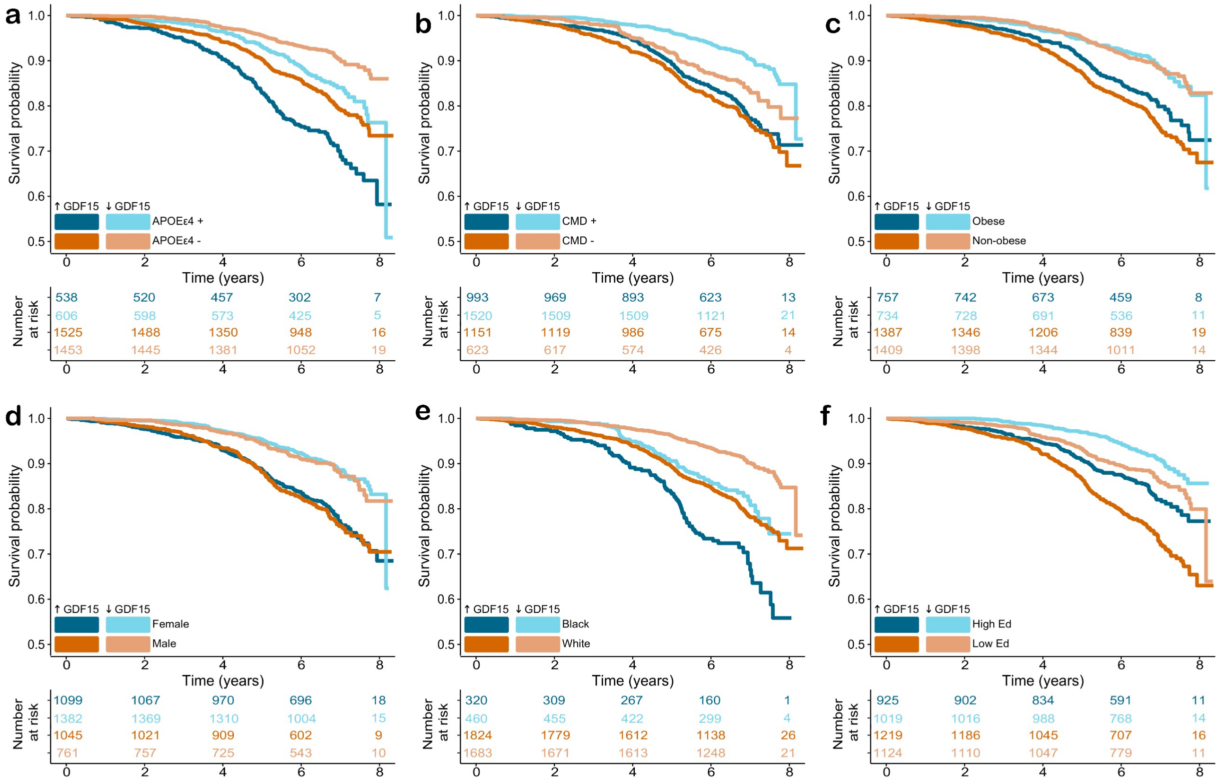

Fig. S7. Kaplan-Meier curves showing plasma GDF15's relationship to the probability of remaining free of ACD across late-life (7-year) in the Atherosclerosis Risk in Communities (ARIC) study.

Associations of plasma GDF15 (high/low; median split) with late-life (7-year) ACD risk stratified by **a.** *APOEε4* status, **b.** cardiometabolic disease, **c.** obesity, **d.** sex, **e.** race, and **f.** educational attainment.

Abbreviations: ACD; all-cause dementia; CMD, cardiometabolic disease; Ed, education.

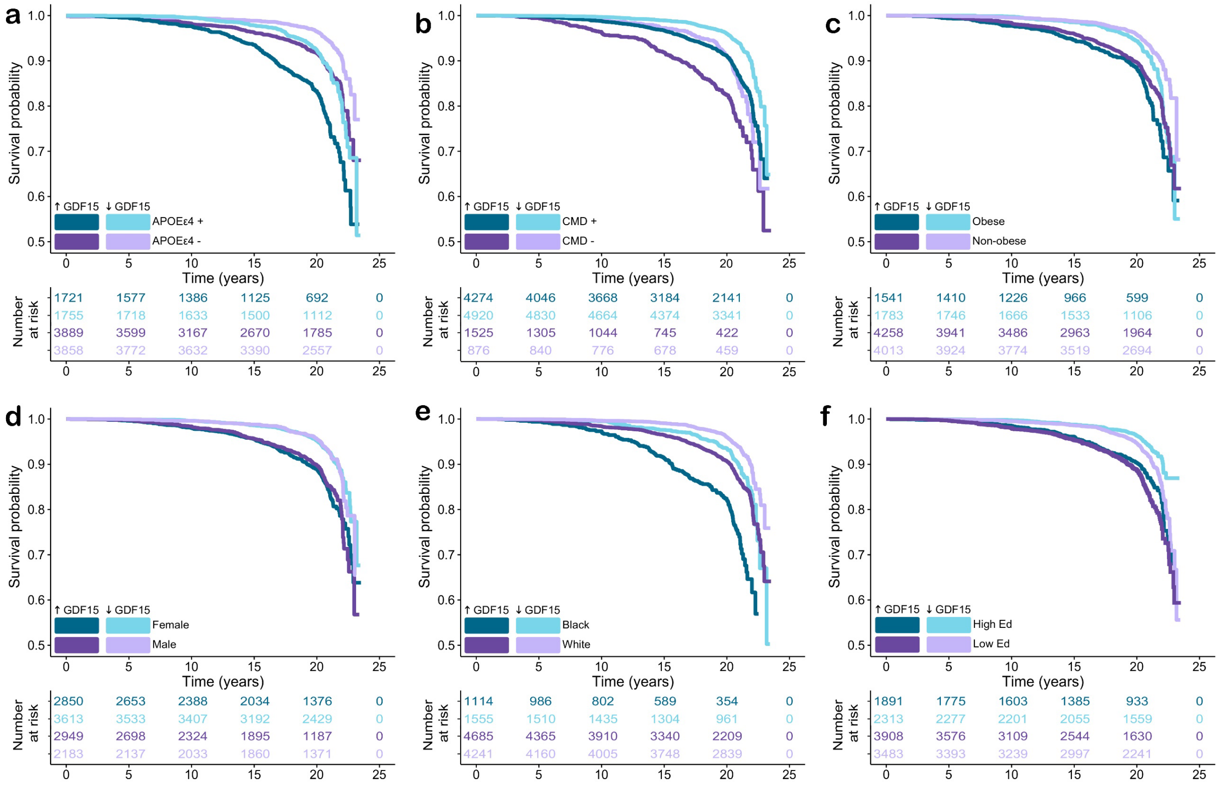

Fig. S8. Kaplan-Meier curves showing plasma GDF15's relationship to the probability of remaining free of ACD across midlife (20-year) in the Atherosclerosis Risk in Communities (ARIC) study.

Associations of plasma GDF15 (high/low; median split) with midlife (20-year) ACD risk stratified by **a.** *APOEε4* status, **b.** cardiometabolic disease, **c.** obesity, **d.** sex, **e.** race, and **f.** educational attainment.

Abbreviations: ACD, all-cause dementia; CMD, cardiometabolic disease; Ed, education.

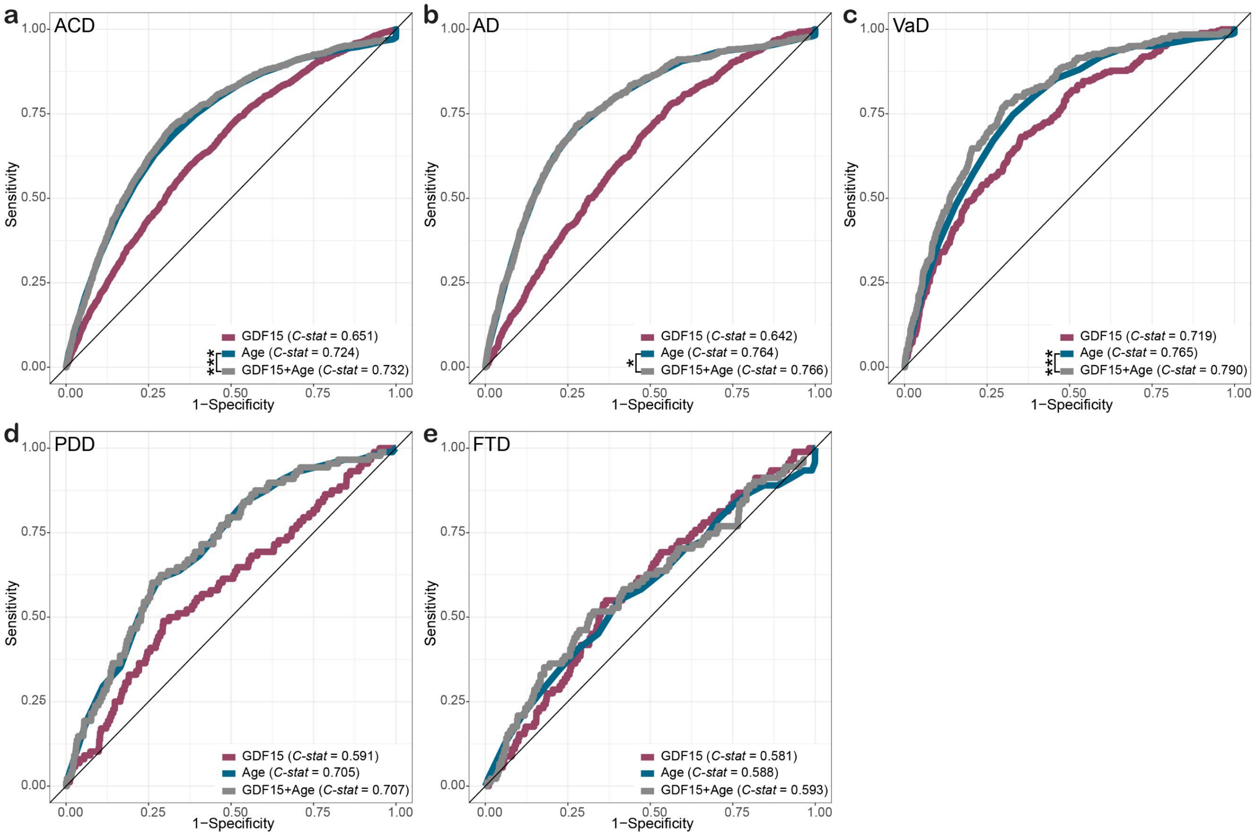

Fig. S9. Predictive performance of plasma GDF15 for dementia risk by etiology in the UK Biobank (14-year follow-up).

ROC curves representing the classification of 14-year incident dementia status by GDF15 levels alone, age alone, and GDF15 combined with age according to dementia subtypes, including **a.** all-cause dementia (ACD), **b.** Alzheimer's disease (AD), **c.** vascular dementia (VaD), **d.** Parkinson's disease dementia (PDD), and **e.** frontotemporal dementia (FTD).
p: ***<0.001; *<0.05

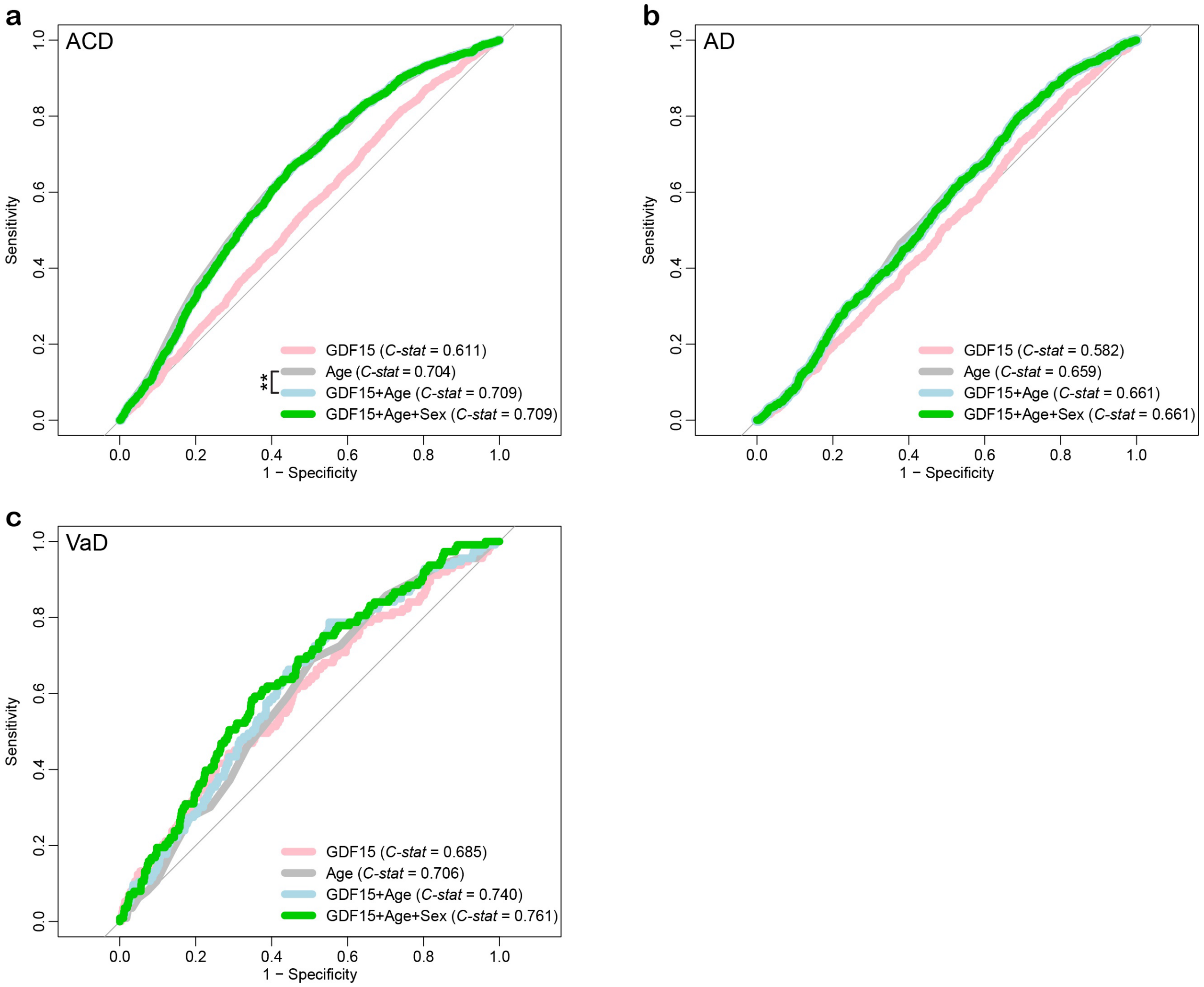

Fig. S10. Predictive performance of plasma GDF15 for dementia risk by etiology in AGES-Reykjavik (17-year follow-up).

ROC curves representing the classification of 17-year incident dementia status by GDF15 levels alone, age alone, GDF15 combined with age, and GDF15 combined with age and sex according to dementia subtypes, including **a.** all-cause dementia (ACD), **b.** Alzheimer's disease (AD), and **c.** vascular dementia (VaD).

p: **<0.01

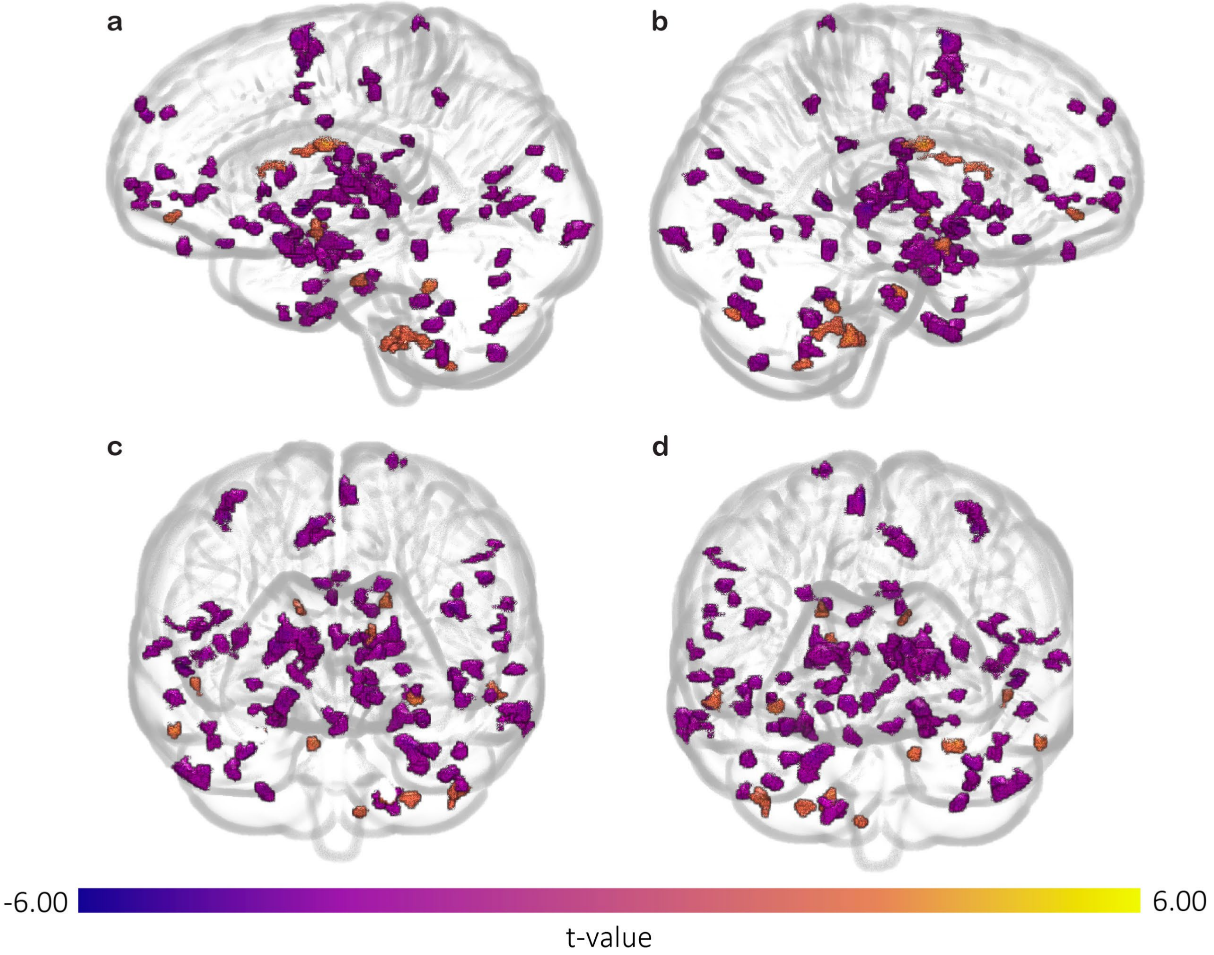

Fig. S11. Voxel-based morphometry associations of plasma GDF15 with gray matter.

Crystal brains depict associations of plasma GDF15 with gray matter as viewed from the **a.** left, **b.** right, **c.** anterior, and **d.** posterior. All clusters shown pass FDR<0.05 correction. Results derived from linear regression models adjusted for age, sex, race, education, *APOEε4*, eGFR, intracranial volume, and a comorbidity index.

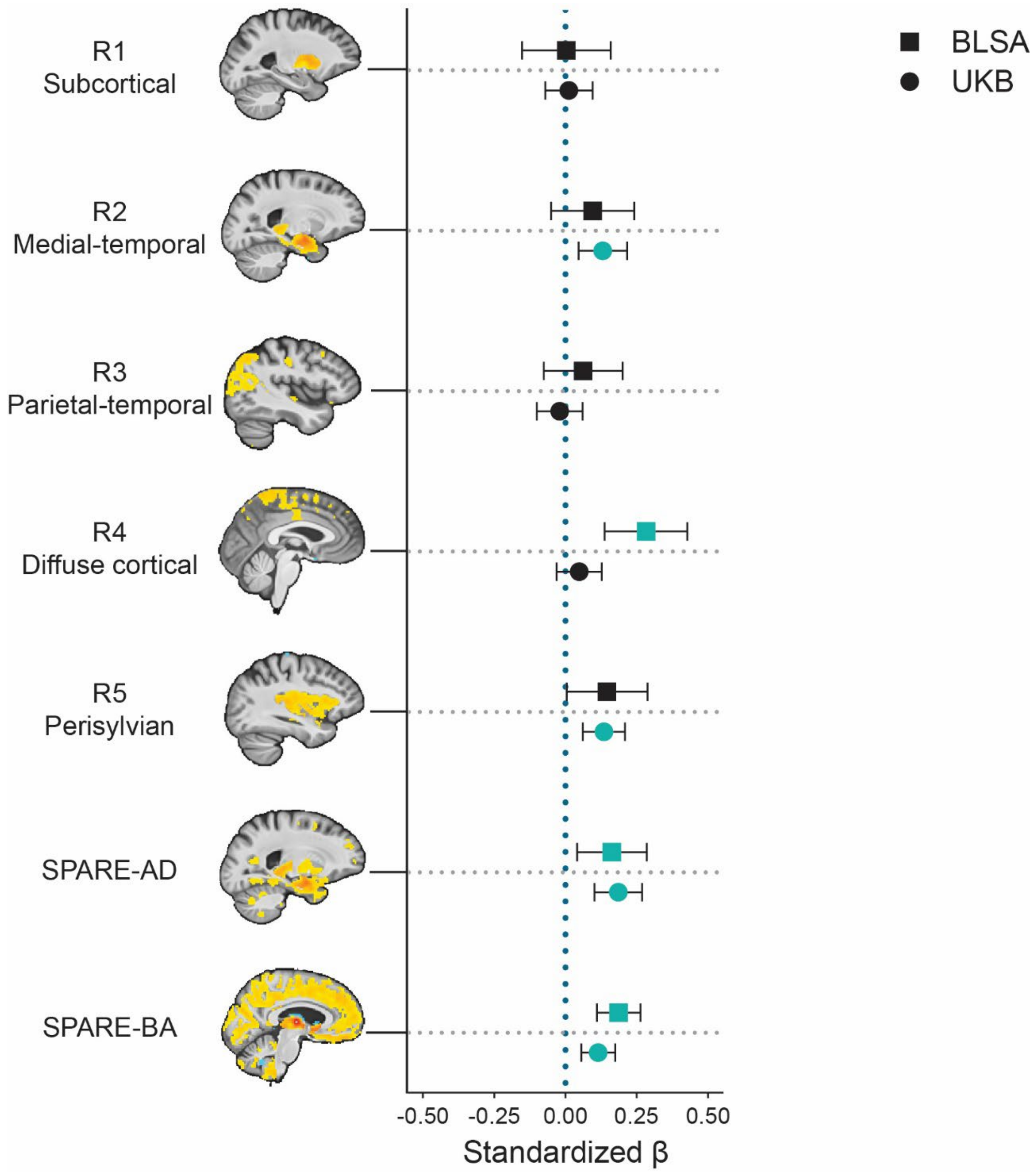

Fig. S12. GDF15 relationship with machine-learning derived patterns of brain atrophy in the BLSA and UKB.

SPARE-AD and SPARE-BA were computed with support vector machine models to distinguish cognitively normal from clinically diagnosed AD individuals and to estimate the biological age of a participant's brain structure, respectively. R index measures were computed with a semi-supervised deep representation learning approach (Surreal-GAN) to capture heterogenous brain volume differences between younger (<50 years old) and older (>50 years old) adults. Images on the left y-axis are representative voxel-wise images from BLSA participants.

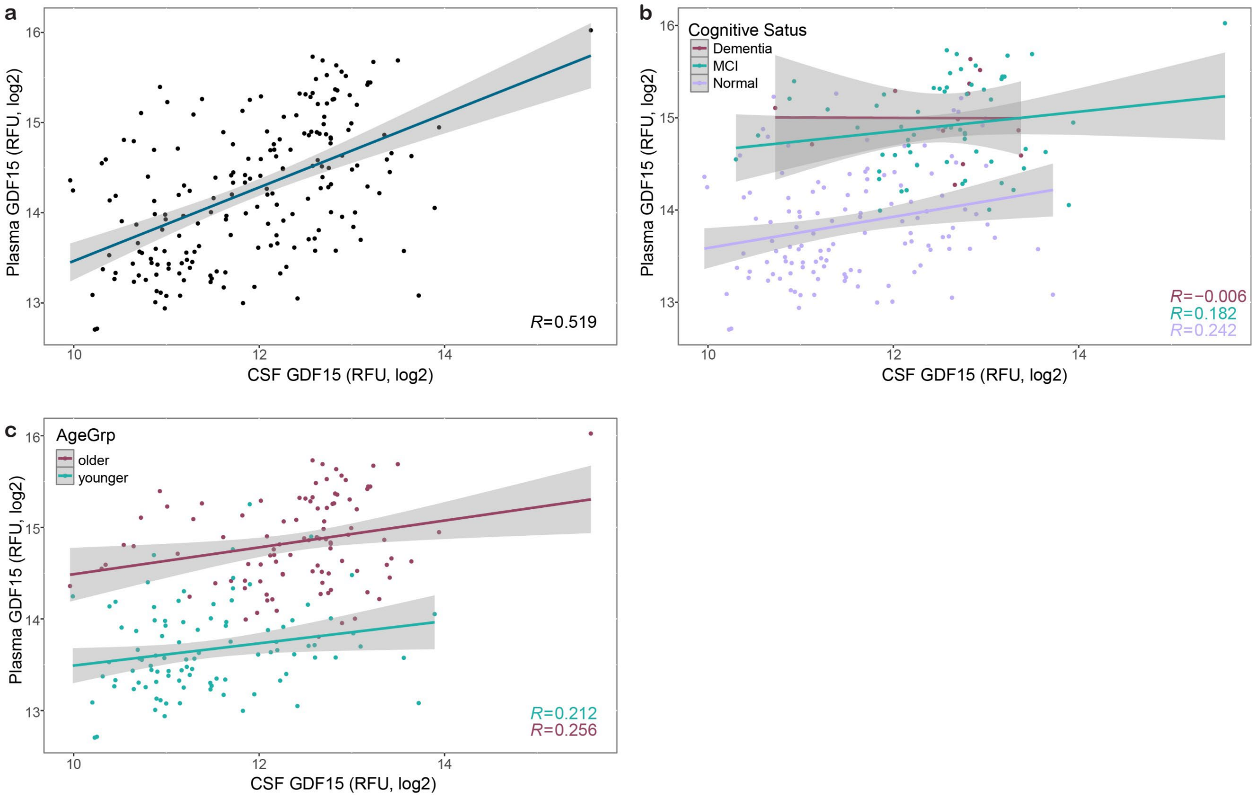

Fig. S13. Correlation of plasma GDF15 and CSF GDF15 in the JHNC Cohort.

Scatter plots depicting CSF GDF15 (x) and plasma GDF15 (y) in the Johns Hopkins Neurology Clinic (JHNC) Cohort **a.** full sample (*N*=194), **b.** stratified by cognitive status: normal (*N*=121), mild cognitive impairment (MCI; *N*=60), and dementia (*N*=13), **c.** stratified by age group (median split at 56.5yo): older (*N*=97) and younger (*N*=97).

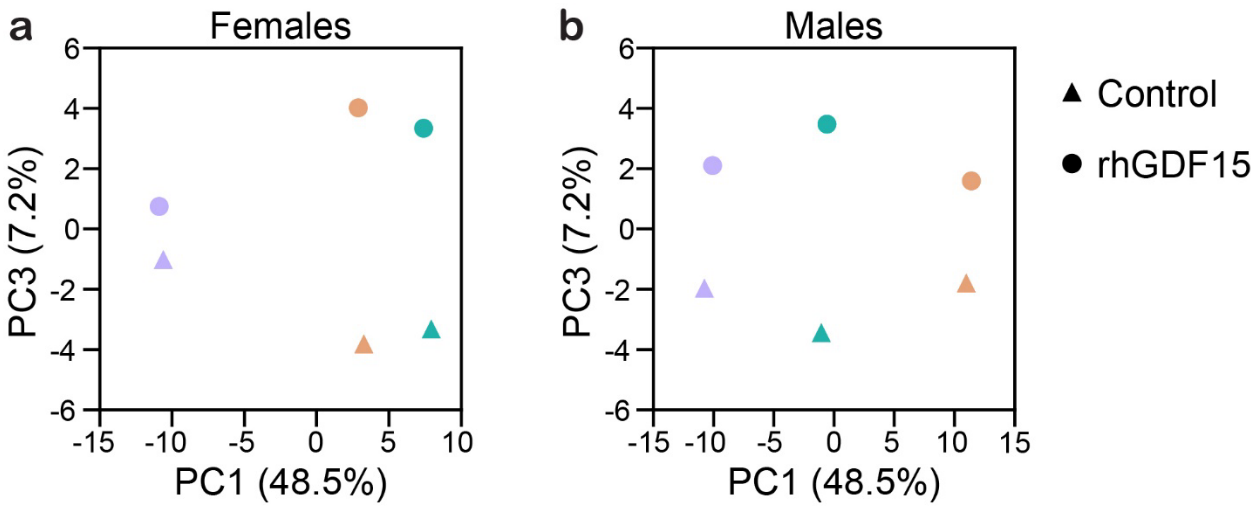

Fig. S14. PCA analyses of mass spectrometry results on macrophages post-exposure to rhGDF15.

Principal component analyses (PCA) results showing the grouping of rhGDF15-treated macrophages compared to control vehicle based on differentially expressed proteins in **a.** females (*N*=3) and **b.** males (*N*=3).

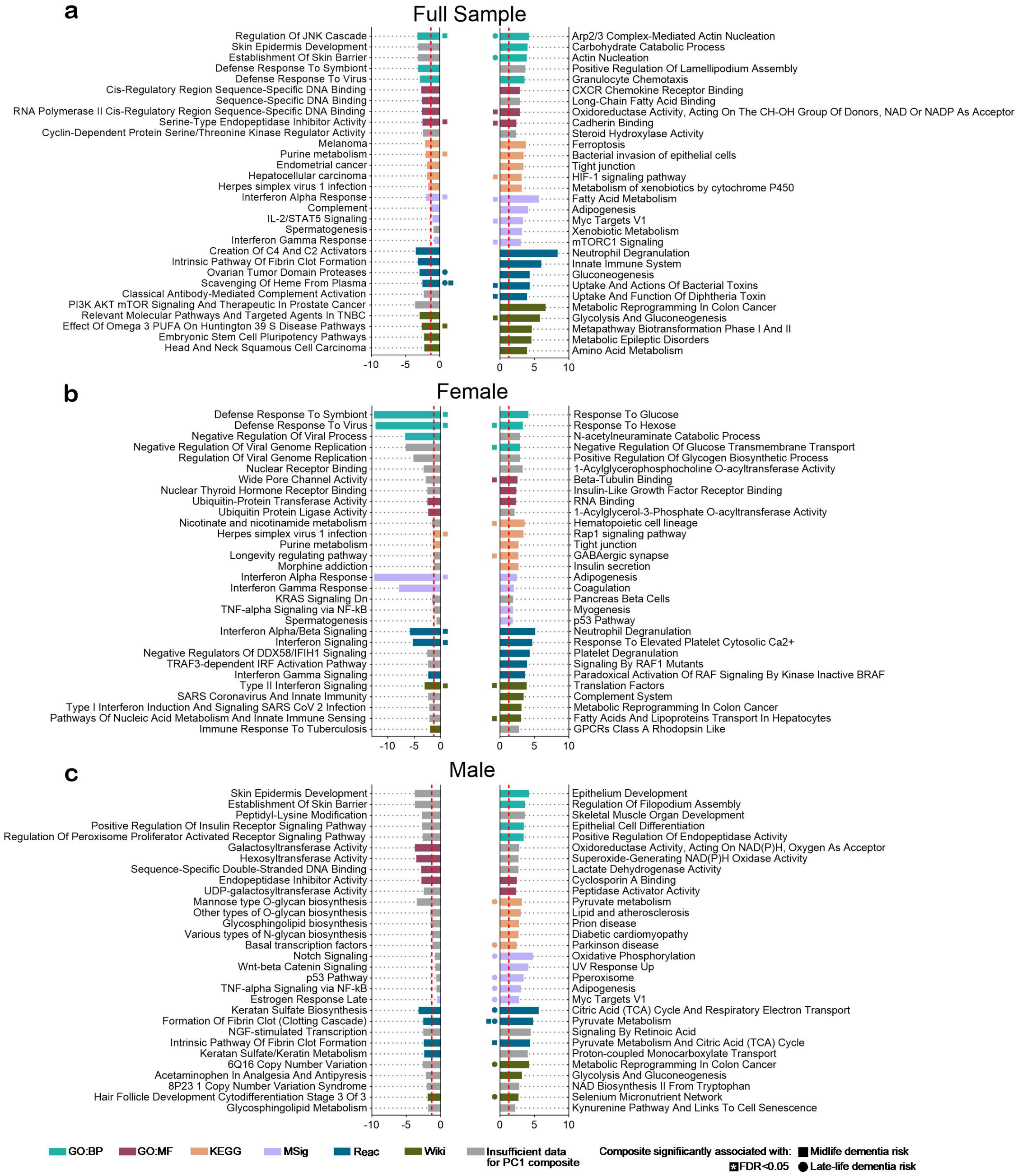

Fig. S15. Pathways enriched following treatment with rhGDF15 compared to controls. Proteins significantly affected by rhGDF15 treatment were analyzed for pathway enrichment using Enrichr (*100-102*) in **a.** the full sample of cell donors (*N*=6), **b.** female donors only (*N*=3), and **c.** male donors only (*N*=3). For each comparison, the top five most annotated biological pathways were identified separately for upregulated and downregulated proteins. Gray bars indicate pathways for which no overlapping proteins were detected between the two measurement modalities—cell lysate mass spectrometry and ARIC participant plasma—preventing the calculation of principal component scores for those pathways. For additional annotation, see **table S20**.

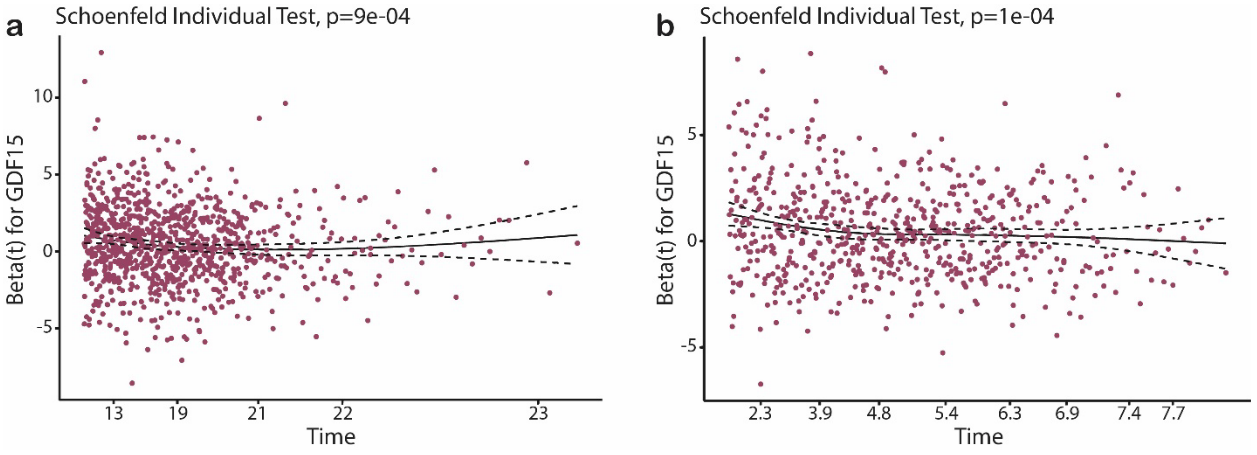

Fig. S16. ARIC Schoenfeld residuals.

Schoenfeld residuals for **a.** midlife and **b.** late-life GDF15 in the Atherosclerosis Risk in Communities (ARIC) study.

Table S1. Baseline demographics for all cohorts

Values are displayed as means (SD) for continuous variables and frequency (column percentages) for categorical variables unless otherwise specified.
See accompanying .xlsx file.

Table S2. External validation of GDF15 measurements

Results derived from previously reported unadjusted spearman correlations
See accompanying .xlsx file.

Table S3. GDF15 expression in cell and tissue types

Cell and tissue expression were taken from the Human Protein Atlas (www.proteinatlas.org).
Abbreviations: nTPM, normalized transcripts per million
See accompanying .xlsx file.

Table S4. Association between incident dementia and plasma GDF15 in ARIC at late-life (7yr) and midlife (20yr)

Late-life analyses related GDF15 measured at ARIC visit 5 to dementia risk occuring through ARIC visit 7. Midlife analyses related GDF15 measured at ARIC visit 2 to dementia risk occurring through ARIC visit 5. Results derived from Cox proportional hazards models were adjusted for baseline age, sex, race-center, education, APOE ε4 status, body mass index, diabetes, hypertension, smoking status, and eGFR-creatinine.
Estimates represent difference in dementia risk per log2 increase (doubling) of SOMAmer level.
Abbreviations: ARIC, Atherosclerosis Risk in Communities; CI, confidence interval; LCI, lower confidence interval; UCI, upper confidence interval; CMD, cardiometabolic disease; Ed, education
See accompanying .xlsx file.

Table S5. Association between incident all-cause dementia (stratified) and etiology-specific dementia with plasma GDF15 in the UK Biobank (14yr)

Results derived from Cox proportional hazards regression models adjusted for age at assessment, sex, highest education level attained, study site, BMI, eGFR (kidney function), prevalent type-2 diabetes, high cholesterol, and APOEε4 status.
Abbreviations: eGFR, estimated glomerular filtration rate; SE, standard error; LCI, lower confidence interval; UCI, upper confidence interval; CMD, cardiometabolic disease; Ed, education; ACD, all-cause dementia; AD, Alzheimer's disease; VaD, vascular dementia; PDD, Parkinson's disease dementia; FTD, frontotemporal dementia
See accompanying .xlsx file.

Table S6. Association between incident all-cause dementia (stratified) and etiology-specific dementia with plasma GDF15 in the AGES-Reykjavik Study (15yr)

Results derived from Cox proportional hazards regression models were adjusted for age at assessment, sex, highest education level attained, APOEε4 status, study site, BMI, eGFR, prevalent type-2 diabetes, and high cholesterol.
Abbreviations: eGFR, estimated glomerular filtration rate; SE, standard error; LCI, lower confidence interval; UCI, upper confidence interval; CMD, cardiometabolic disease; Ed,

education; ACD, all-cause dementia; AD, Alzheimer's disease; VaD, vascular dementia; PDD, Parkinson's disease dementia; FTD, frontotemporal dementia
See accompanying .xlsx file.

Table S7. Multicohort meta-analysis of associations between plasma GDF15 and incident dementia

Associations between plasma GDF15 levels and dementia risk were estimated in individual cohorts and meta-analyses. Individual cohort analyses include ARIC (Visits 2 and 5), UKB, NILS-LSA, and AGES. Meta-analyses were performed for midlife cohorts (ARIC Visit 2, UKB, NILS-LSA), late-life cohorts (ARIC Visit 5, AGES), and all cohorts combined, using both fixed-effects and random-effects models.
See accompanying .xlsx file.

Table S8. Mendelian Randomization - Post-selection Inference (MR-SPI)

Results derived from two-sample Mendelian Randomization Post Selection Inference (Yao et al (*23*).). Instrumental variables were obtained from ARIC for primary analyses (Zhang et al (*34*).) and deCODE for secondary analyses (Ferkingstad et al (*27*).).
See accompanying .xlsx file.

Table S9. GDF15 association with single nucleotide polymorphisms (SNPs).

SNPs were identified based on previous genome-wide association studies (GWASs) from the ARIC cohort, a GDF15 GWAS meta-analysis, or from the OnTime Database (OTDB) (see footnotes); indicates insignificant finding. Closest target genes are in sequential order and were determined using OpenTarget Genetics canonical transcriptional start site distance. Bolded terms are related to dementia. Gray text indicates and insignificant relationship.
See accompanying .xlsx file.

Table S10. GDF15 Associations with MRI Dementia Endophenotypes (ARIC)

Multivariable regression models were adjusted for demographic variables (age, sex, race-center, education), cardiovascular risk factors (body mass index, diabetes, hypertension, smoking status), kidney function (eGFR-creatinine), and APOEε4 alleles. Models including brain volume outcomes were additionally adjusted for estimated intracranial volume.
Abbreviations: CMD, cardiometabolic disease; Ed, education; eGFR, estimated glomerular filtration rate; LCI, lower confidence interval; OR, odds ratio; UCI, upper confidence interval
See accompanying .xlsx file.

Table S11. GDF15 Associations with MRI Dementia Endophenotypes (BLSA)

Association of plasma candidate proteins with MRI-defined dementia endophenotypes in BLSA participants. SPARE-AD is a measure of the extent to which the pattern of brain volume loss is consistent with that observed in Alzheimer’s disease; SPARE-BA is a measure of typical brain changes associated with age. Results were derived from linear regression models adjusted for age, sex, race, education, APOEε4 status, eGFR, and comorbidity index. Total brain volume analyses adjusted for intracranial volume.
Abbreviations: eGFR, estimated glomerular filtration rate; SE, standard error; OR, odds ratio; CMD, cardiometabolic disease; Ed, education
See accompanying .xlsx file.

Table S12. Voxel-based morphometry analysis of the effects of GDF15 on gray matter volume

Results derived from linear regression analysis were adjusted for age, sex, race, education, APOEε4 carrier status, presence of cardiovascular/metabolic disease, eGFR, and total intracranial volume. Only the top 20 largest clusters are presented. Results are corrected for multiple comparisons (FDR<0.05). Results are presented using Montreal Neurological Institute (MNI) coordinates.
Abbreviations: BLSA, Baltimore Longitudinal Study of Aging; dmPFC, dorsomedial prefrontal cortex; ITG, Inferior temporal gyrus; MTG, Middle temporal gyrus.
See accompanying .xlsx file.

Table S13. GDF15 associations with machine learning-derived patterns of brain atrophy in the BLSA and UKB

Results were derived from multiple linear regression models adjusting for age, sex, race, APOEε4, education, eGFR and a comorbidity index (i.e., obesity, hypertension, diabetes, cancer, ischemic heart disease, chronic heart failure, chronic kidney disease and chronic obstructive pulmonary disease).
Abbreviations: BLSA, Baltimore Longitudinal Study on Aging; UKB, UK Biobank
See accompanying .xlsx file.

Table S14. GDF15 Associations with ADRD Biomarkers (ARIC)

Associations of plasma GDF15 with Alzheimer's disease and neurodegenerative plasma biomarkers in BLSA participants. Estimates derived from linear regression models that were adjusted for age, sex, race, education, APOEε4 status, eGFR, and comorbidity index.
Abbreviations: ARIC, Atherosclerosis Risk in Communities; STD, standard deviation; CMD, cardiometabolic disease; Ed, education; Aβ42/40, amyloid-beta 42 to 40 ratio; GFAP, glial fibrillary acidic protein; NfL, neurofilament light; pTau, phosphorylated-tau
See accompanying .xlsx file.

Table S15. GDF15 Associations with ADRD Biomarkers (BLSA)

Associations of plasma GDF15 with Alzheimer's disease and neurodegenerative plasma biomarkers in BLSA participants. Estimates derived from linear regression models were adjusted for age, sex, race, education, APOEε4 status, eGFR, and comorbidity index.
Abbreviations: BLSA, Baltimore Longitudinal Study on Aging; SE, standard error; FDR, false discovery rate; Aβ42/40, amyloid-beta 42 to 40 ratio; GFAP, glial fibrillary acidic protein; NfL, neurofilament light; pTau, phospho-tau; CMD, cardiometabolic disease; Ed, education
See accompanying .xlsx file.

Table S16. Association of GDF15 with PET-defined amyloid-positive status

Association of plasma GDF15 with PET-defined amyloid-positive status. Results derived from logistic regression models were adjusted for age, sex, race, education, APOEε4 status, and eGFR, hypertension, diabetes, and BMI at the time of protein measurement. Odds ratios (ORs) and 95% confidence intervals (CIs) represent the odds of amyloid-positive status per each log2 increase (doubling) in protein abundance.
See accompanying .xlsx file.

Table S17. GDF15 Correlations Across Biofluids (JHNC cohort)

Spearman correlations were calculated for GDF15 measured in either plasma or CSF with the full plasma or CSF proteome (7000+ aptamers) using the Johns Hopkins Neurology Clinic cohort (N=194). N/As induced by missing protein measurements between biofluids (i.e., protein that is measured in CSF but not plasma).
Abbreviations: CSF, cerebrospinal fluid
See accompanying .xlsx file.

Table S18. Pathways Enrichment for GDF15 Correlations Across Biofluids (JHNC cohort)

Enrichment was done using Enrichr, a public resources that pulls information from other publically available bioinformatic databases based on proteomic input. Databases utilized were Gene Ontology: Biological Processes (GO:BP), Gene Ontology: Molecular Function (GO:MF), KEGG, MSig Database (MSig DB), Reactome, and WikiPathways (Wiki). Cutoffs for enrichment were determined based Spearman correlation coefficients rather than p-values due to vast amounts of significance (see table S17). Repeated protein names were removed and concatonated terms were seperated prior to enrichment. Each analysis was ran with a background dataset that represented all proteins measured by SomaScan v4.1 after duplicated Entrez Gene Names were removed (6380 proteins).
See accompanying .xlsx file.

Table S19. Plasma GDF15 immune-specific phenome-wide association study (IPheWAS)

The GDF15 PheWAS integrated cis-protein quantitative trait loci for plasma GDF15 defined in the Atherosclerosis Risk in Communities (ARIC) study using data from n=7213 European-American participants. Cis-regualted protein abundance was assocaited with 133 immune traits (outcomes) defined using their respective genome-wide association study (GWAS) summary statistics. Cis-pQTL models were derived and defined in Zhang et al (*34*). GDF15's aptomer ID in SomaScan is SeqId_4374.45, and the pQTL of interest here was rs16982345, which had an R^2 of 0.177 and z-value of 35.78. The gene for GDF15 is located on chromosome 19 with gene start at 18374731 and end at 18389176. Invariant statistics for GDF15 across all phenotypes are as follows: Hypothesis sum of squares (HSQ)=0.282, number of SNPs=46, Model CV R^2=0.27, Model CV p-value=0.
See accompanying .xlsx file.

Table S20. Protein mass spectrometry results of macrophage secretome following rhGDF15 exposure compared to control

PBMC-derived macrophages from 6 donors (50% female) were exposed 0.5ug/mL of either rhGDF15 or PBS; 24 hours later, secretome protein changes were quantified via mass spectrometry. Only proteins detected in at least two out of three replicates in at least one experimental condition were included for analysis. Data were normalized using the variance stabilization normalization (VSN) method with function “normalize_vsn”, and missing values were imputed using random draws from a Gaussian distribution centered around a minimal value with function “MinProb”. The differential protein expression analysis was performed based on linear models and empirical Bayes statistics using limma via function ”test_diff” in DEP package, with adjustment for variability associated with the donor. Analyses were conducted in the full sample (N=6), females only (N=3), and males only (N=3).

Abbreviations: PBMC, peripheral blood mononuclear cells; rhGDF15, recombinant human GDF15
See accompanying .xlsx file.

Table S21. Pathway enrichment following exposure to rhGDF15 compared to controls

Enrichment was done using Enrichr (maayanlab.cloud/Enrichr/), a public resources that pulls information from other publically available bioinformatic databases based on proteomic input. Databases utilized were Gene Ontology: Biological Processes (GO:BP), Gene Ontology: Molecular Function (GO:MF), KEGG, MSig Database (MSig DB), Reactome, and WikiPathways (Wiki). Cutoffs for enrichment were determined based on p-value<0.05. Repeated protein names were removed and concatonated terms were seperated prior to enrichment. Each analysis was ran with a background dataset that represented all proteins measured by SomaScan v4.1 after duplicated Entrez Gene Names were removed (6380 proteins). Grayed out terms are insignificant.
See accompanying .xlsx file.

Table S22. Proteins associated with dementia risk at both mid- and late-life and correlated with plasma GDF15 across biofluids: Mediation affects

Of the 610 proteins that were significantly influenced by exposure to rhGDF15 in at least one of these three experimental groups (full sample, females only, males only), 29 proteins were associated with future dementia risk when measured during midlife and during late-life (ARIC), and 6 of these proteins (CD300A, CD14, UBE2E1, HERC5, CTSV, MAN1C1) also maintained a significant correlation with plasma GDF15 when measured in both CSF and plasma (JHNC). The extent to which these proteins mediate the effect of GDF15 on incident dementia risk is depicted below. Percent mediation was derived from the difference in hazard ratio from the association of GDF15 and dementia risk with and without individual proteins included as a covariate.
Abbreviations: ARIC, Arthrosclerosis Risk in Communities; JHNC, Johns Hopkins Neurology Clinic; HR, hazard ratio; SE, standard error; LCI, lower confidence interval; UCI, upper confidence interval
See accompanying .xlsx file.

Table S23. Experimentally enriched pathway principal component score associations with ARIC incident dementia

Late-life analyses related 120 first principal component scores (representing biological pathways/processes) of proteins measured at ARIC visit 5 to dementia risk occuring through ARIC visit 7 (7yr follow-up). Midlife analyses related the same 120 first principal component scores of proteins measured at ARIC visit 2 to dementia risk occuring through ARIC visit 5 (20yr follow-up). Results were derived from Cox proportional hazards models were adjusted for baseline age, sex, race-center, education, APOE ε4 status, body mass index, diabetes, hypertension, smoking status, and eGFR-creatinine. Sex specific pathways were associated with sex-stratified dementia risk.
Estimates represent difference in dementia risk per log2 increase (doubling) of SOMAmer level. Abbreviations: ARIC, Atherosclerosis Risk in Communities; CI, confidence interval; LCI, lower confidence interval; UCI, upper confidence interval

Source naming convention: sample.direction.database#. Sample: Full sample/combined (C); female (F); male (M). Direction: Upregulated (up) or downregulated (down) post-exposure to rhGDF15. Database: Gene Ontology: Biological Processes (GO:BP), Gene Ontology: Molecular Function (GO:MF), KEGG, MSig Database (MSig DB), Reactome (Reac), and WikiPathways (Wiki). #: Order of enrichment significance (1 most significant, 5 least significant)
See accompanying .xlsx file.

Table S24. Experimentally enriched pathway principal component score associations with ARIC incident dementia

For the pathways principal component scores that were significantly associated with dementia risk in ARIC at midlife and late-life (see table S23 for further detail), we provide the proteins within each pathway and how they load on to the component score, as well as their individual associations with dementia risk in ARIC and their individual correlation with circulating GDF15 (plasma and CSF; derived from the JHNC Cohort [table S17]) as true/false columns with directionality if true. % Mediation represents the extent to which GDF15's association with dementia risk are mediated by each component score.
Abbreviations: ARIC, Atherosclerosis Risk in Communities; CI, confidence interval; LCI, lower confidence interval; UCI, upper confidence interval; pgppCorr, correlation of protein with plasma GDF15 when measured in plasma; pgcpCorr, correlation of protein with plasma GDF15 when measured in CSF
Pathway ID naming convention: sample.direction.database#. Sample: Protien mass spectrometry sample; full sample/combined (C); female (F); male (M). Direction: Upregulated (up) or downregulated (down) post-exposure to rhGDF15. Database: Gene Ontology: Biological Processes (GO:BP), Gene Ontology: Molecular Function (GO:MF), KEGG, MSig Database (MSig DB), Reactome (Reac), and WikiPathways (Wiki). #: Order of enrichment significance (1 most significant, 5 least significant)
See accompanying .xlsx file.
